# Supplementary figures and images for: Oxygen levels at the time of activation determine T cell persistence and immunotherapeutic efficacy (part 1 of 2)
Source: eLife. 2023 May 11;12:e84280. doi: 10.7554/eLife.84280 (PMC10229120; doi:10.7554/eLife.84280)

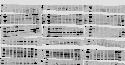

Supplement: Figure 1—source data 1. [file elife-84280-fig1-data1.zip › Figure 1 - Source data 1 - Unedited blots/Fig1C [ABC]/0007876_02 [open in Image Studio Lite ver 5.2]/0007876_02_TH.jpg]

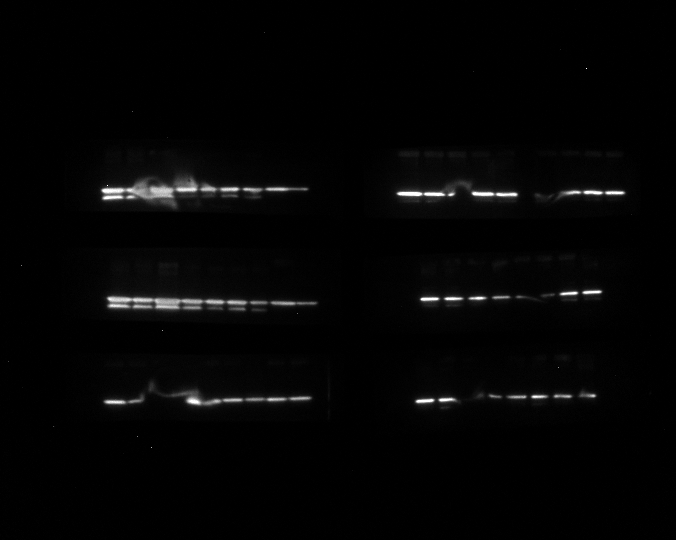

Supplement: Figure 1—source data 1. [file elife-84280-fig1-data1.zip › Figure 1 - Source data 1 - Unedited blots/Fig1E n1 H3 (bottom left)/CHEMI_06292022_191725_(Chemi)_raw.tif]

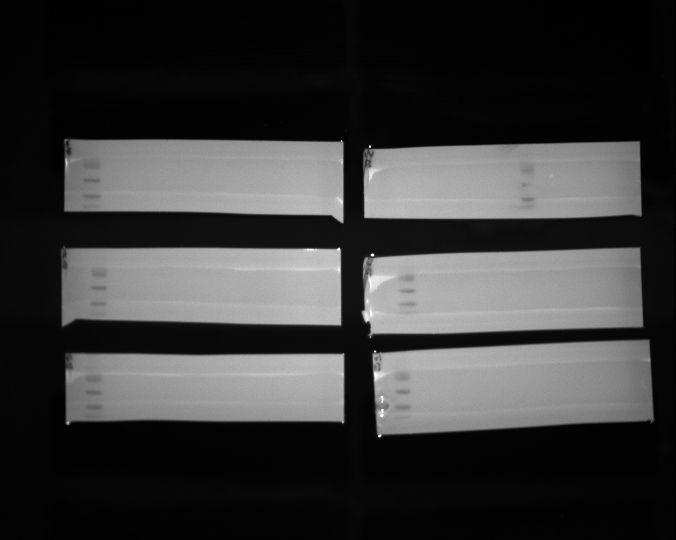

Supplement: Figure 1—source data 1. [file elife-84280-fig1-data1.zip › Figure 1 - Source data 1 - Unedited blots/Fig1E n1 H3 (bottom left)/CHEMI_06292022_191725_(Membrane)_raw.tif]

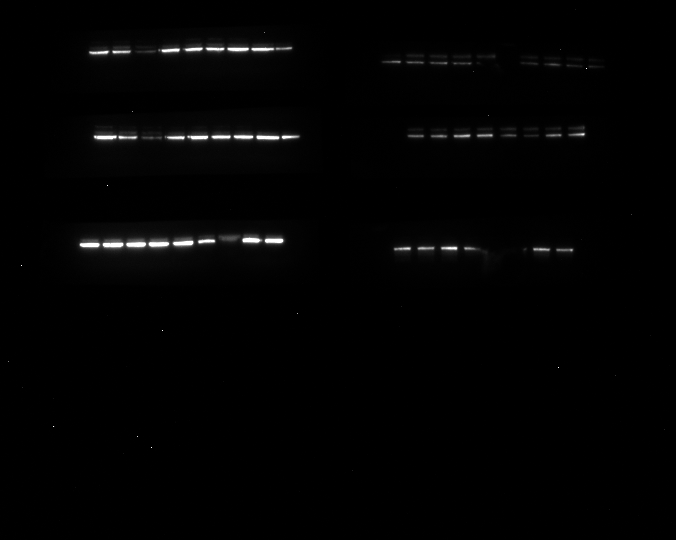

Supplement: Figure 1—source data 1. [file elife-84280-fig1-data1.zip › Figure 1 - Source data 1 - Unedited blots/Fig1E n1 HDAC (bottom left)/CHEMI_07012022_002407_(Chemi)_raw.tif]

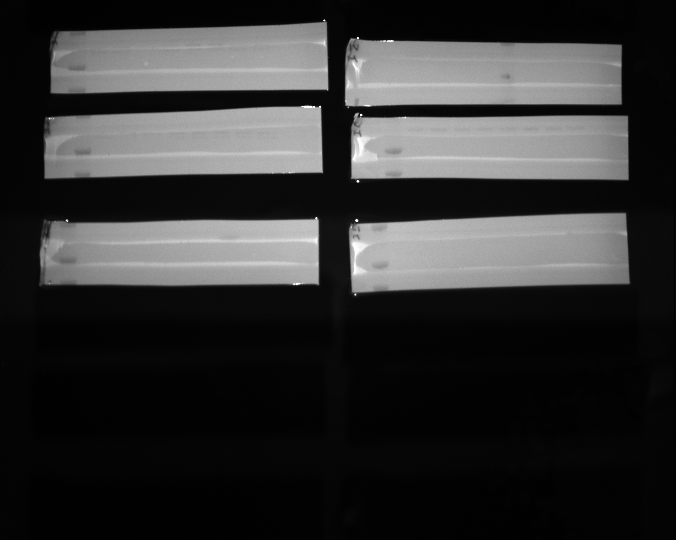

Supplement: Figure 1—source data 1. [file elife-84280-fig1-data1.zip › Figure 1 - Source data 1 - Unedited blots/Fig1E n1 HDAC (bottom left)/CHEMI_07012022_002407_(Membrane)_raw.tif]

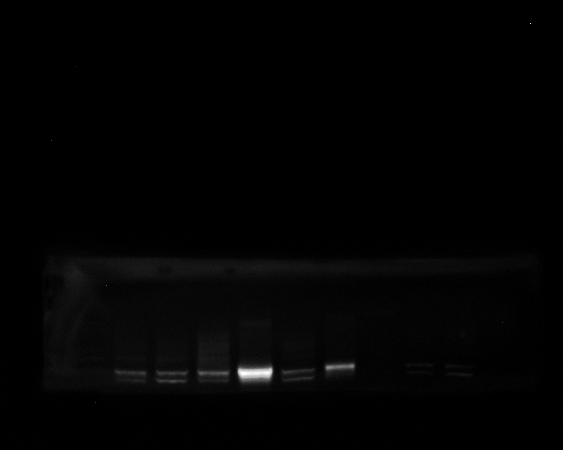

Supplement: Figure 1—source data 1. [file elife-84280-fig1-data1.zip › Figure 1 - Source data 1 - Unedited blots/Fig1E n1 HIF/CHEMI_06302022_184538_(Chemi)_raw.tif]

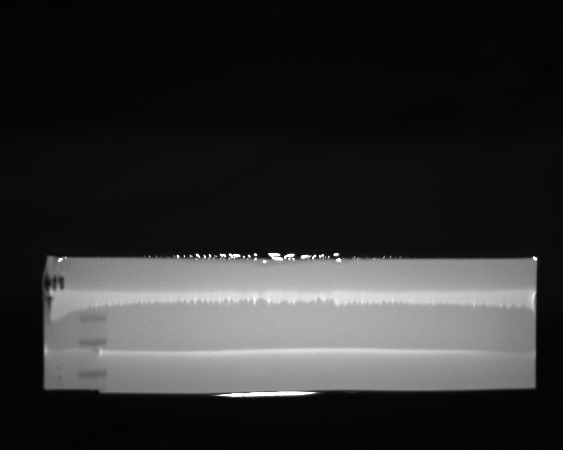

Supplement: Figure 1—source data 1. [file elife-84280-fig1-data1.zip › Figure 1 - Source data 1 - Unedited blots/Fig1E n1 HIF/CHEMI_06302022_184538_(Membrane)_raw.tif]

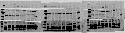

Supplement: Figure 1—source data 1. [file elife-84280-fig1-data1.zip › Figure 1 - Source data 1 - Unedited blots/Fig1E n2n3/0008142_02 [open in Image Studio Lite ver 5.2]/0008142_02_TH.jpg]

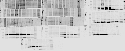

Supplement: Figure 1—figure supplement 1—source data 1. [file elife-84280-fig1-figsupp1-data1.zip › Fig 1 - fig S1 - Source data 1 - Unedited blots/Figure 1 - figure supplement 1B [open in Image Studio Lite version 5.2]/0005344_01_TH.jpg]

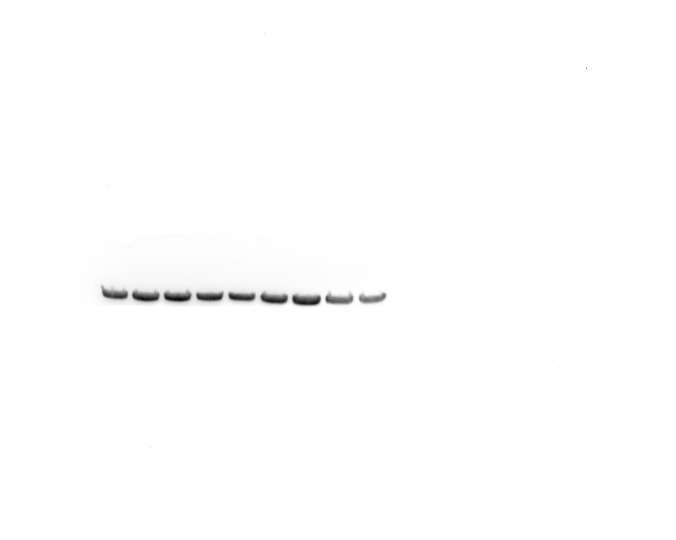

Supplement: Figure 3—figure supplement 1—source data 1. [file elife-84280-fig3-figsupp1-data1.zip › Fig 3 - fig S1 - Source data 1 - Unedited blots/Figure 3 - figure supplement 1D-E/PC070e Actin/pc_21102121_actin_(Chemi)_raw.png]

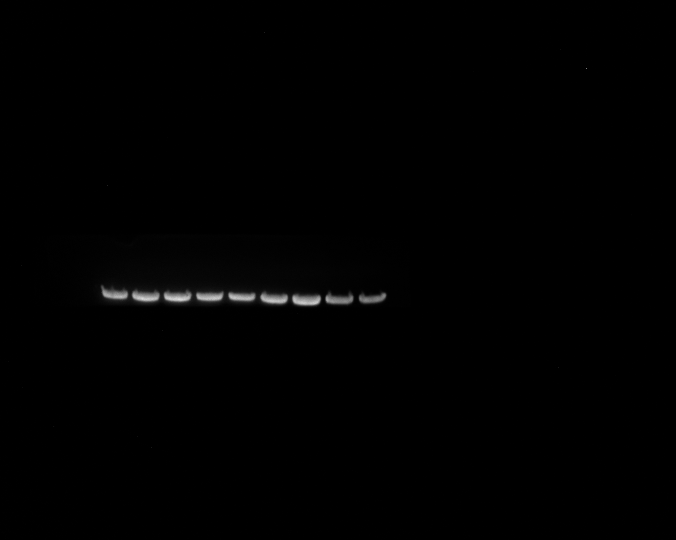

Supplement: Figure 3—figure supplement 1—source data 1. [file elife-84280-fig3-figsupp1-data1.zip › Fig 3 - fig S1 - Source data 1 - Unedited blots/Figure 3 - figure supplement 1D-E/PC070e Actin/pc_21102121_actin_(Chemi)_raw.tif]

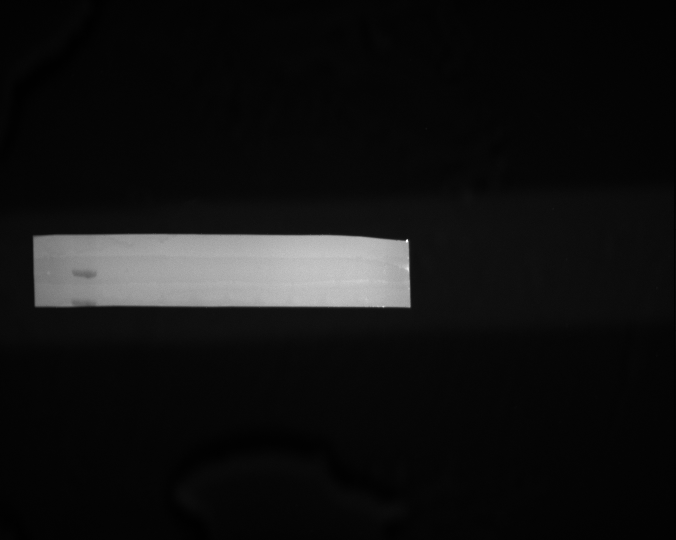

Supplement: Figure 3—figure supplement 1—source data 1. [file elife-84280-fig3-figsupp1-data1.zip › Fig 3 - fig S1 - Source data 1 - Unedited blots/Figure 3 - figure supplement 1D-E/PC070e Actin/pc_21102121_actin_(Membrane)_raw.tif]

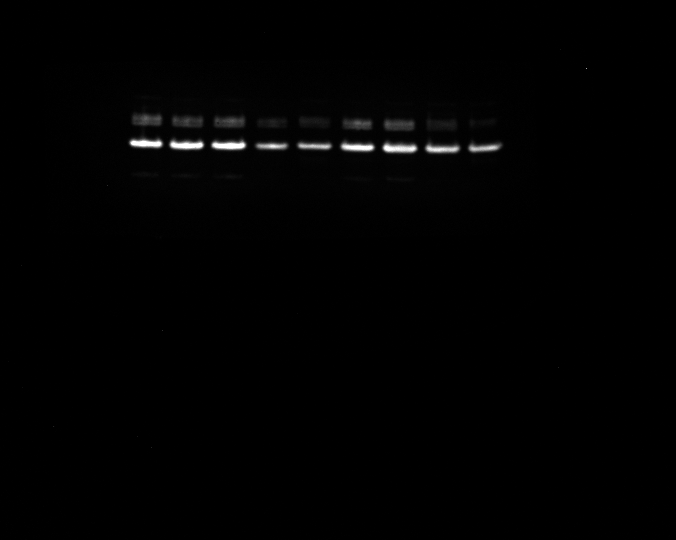

Supplement: Figure 3—figure supplement 1—source data 1. [file elife-84280-fig3-figsupp1-data1.zip › Fig 3 - fig S1 - Source data 1 - Unedited blots/Figure 3 - figure supplement 1D-E/PC070e VHL/pc_21102021_vhl_image2_(Chemi)_raw.tif]

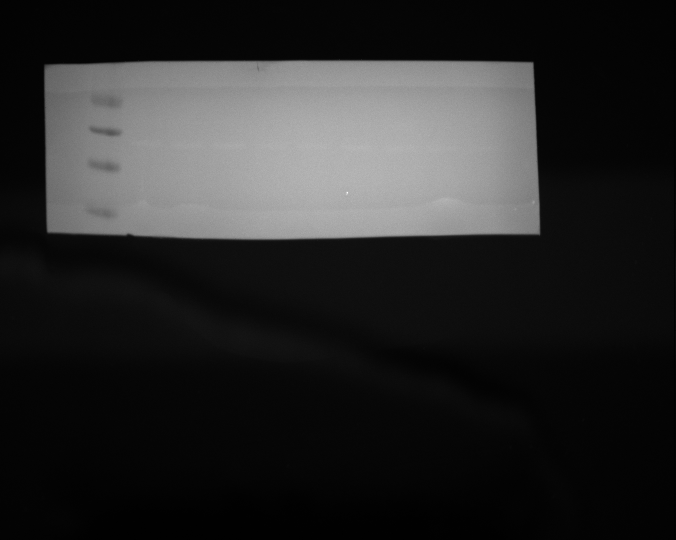

Supplement: Figure 3—figure supplement 1—source data 1. [file elife-84280-fig3-figsupp1-data1.zip › Fig 3 - fig S1 - Source data 1 - Unedited blots/Figure 3 - figure supplement 1D-E/PC070e VHL/pc_21102021_vhl_image2_(Membrane)_raw.tif]

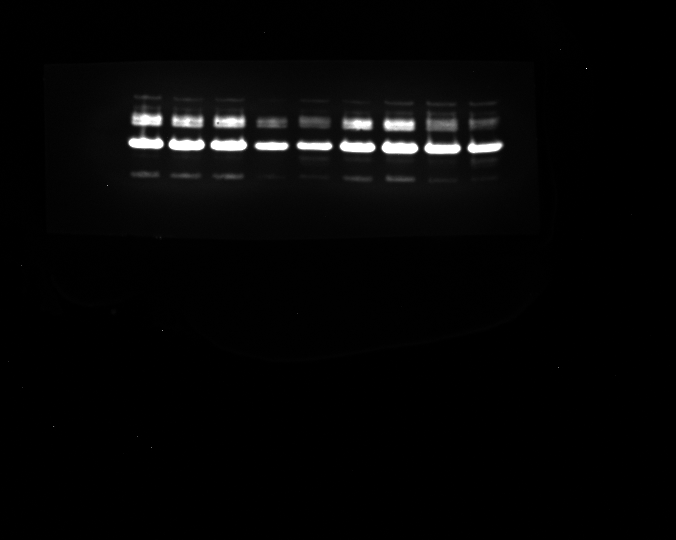

Supplement: Figure 3—figure supplement 1—source data 1. [file elife-84280-fig3-figsupp1-data1.zip › Fig 3 - fig S1 - Source data 1 - Unedited blots/Figure 3 - figure supplement 1D-E/PC070e VHL/pc_21102021_vhl_image3_(Chemi)_raw.tif]

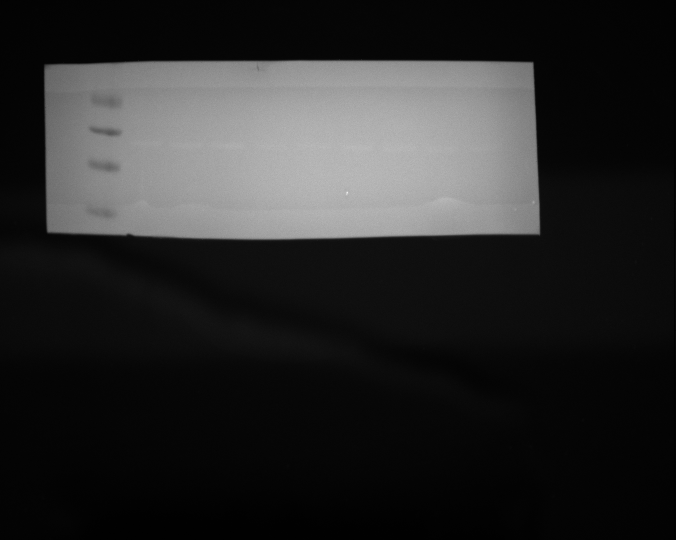

Supplement: Figure 3—figure supplement 1—source data 1. [file elife-84280-fig3-figsupp1-data1.zip › Fig 3 - fig S1 - Source data 1 - Unedited blots/Figure 3 - figure supplement 1D-E/PC070e VHL/pc_21102021_vhl_image3_(Membrane)_raw.tif]

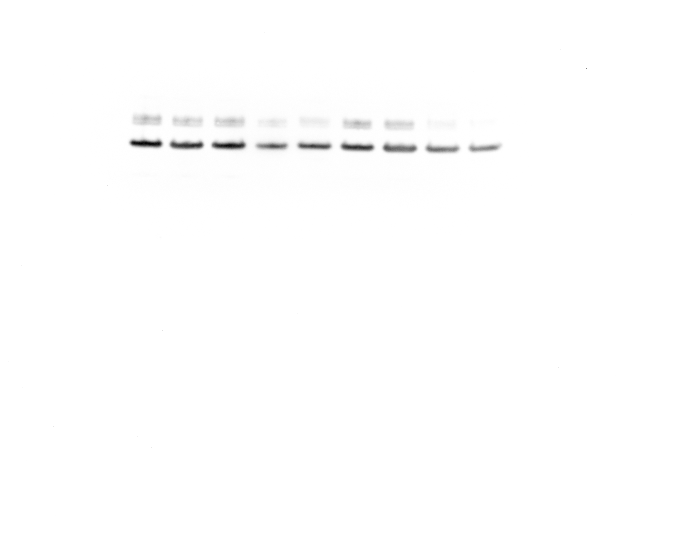

Supplement: Figure 3—figure supplement 1—source data 1. [file elife-84280-fig3-figsupp1-data1.zip › Fig 3 - fig S1 - Source data 1 - Unedited blots/Figure 3 - figure supplement 1D-E/PC070e VHL/pc_211021_vhl_image1_(Chemi)_raw.png]

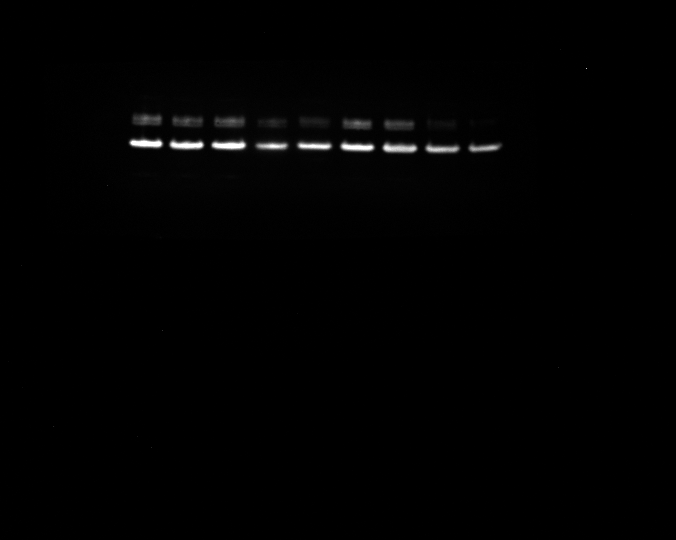

Supplement: Figure 3—figure supplement 1—source data 1. [file elife-84280-fig3-figsupp1-data1.zip › Fig 3 - fig S1 - Source data 1 - Unedited blots/Figure 3 - figure supplement 1D-E/PC070e VHL/pc_211021_vhl_image1_(Chemi)_raw.tif]

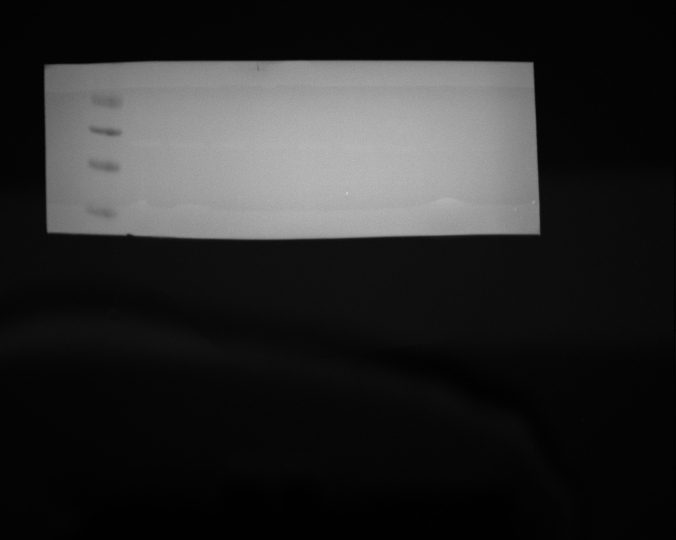

Supplement: Figure 3—figure supplement 1—source data 1. [file elife-84280-fig3-figsupp1-data1.zip › Fig 3 - fig S1 - Source data 1 - Unedited blots/Figure 3 - figure supplement 1D-E/PC070e VHL/pc_211021_vhl_image1_(Membrane)_raw.tif]

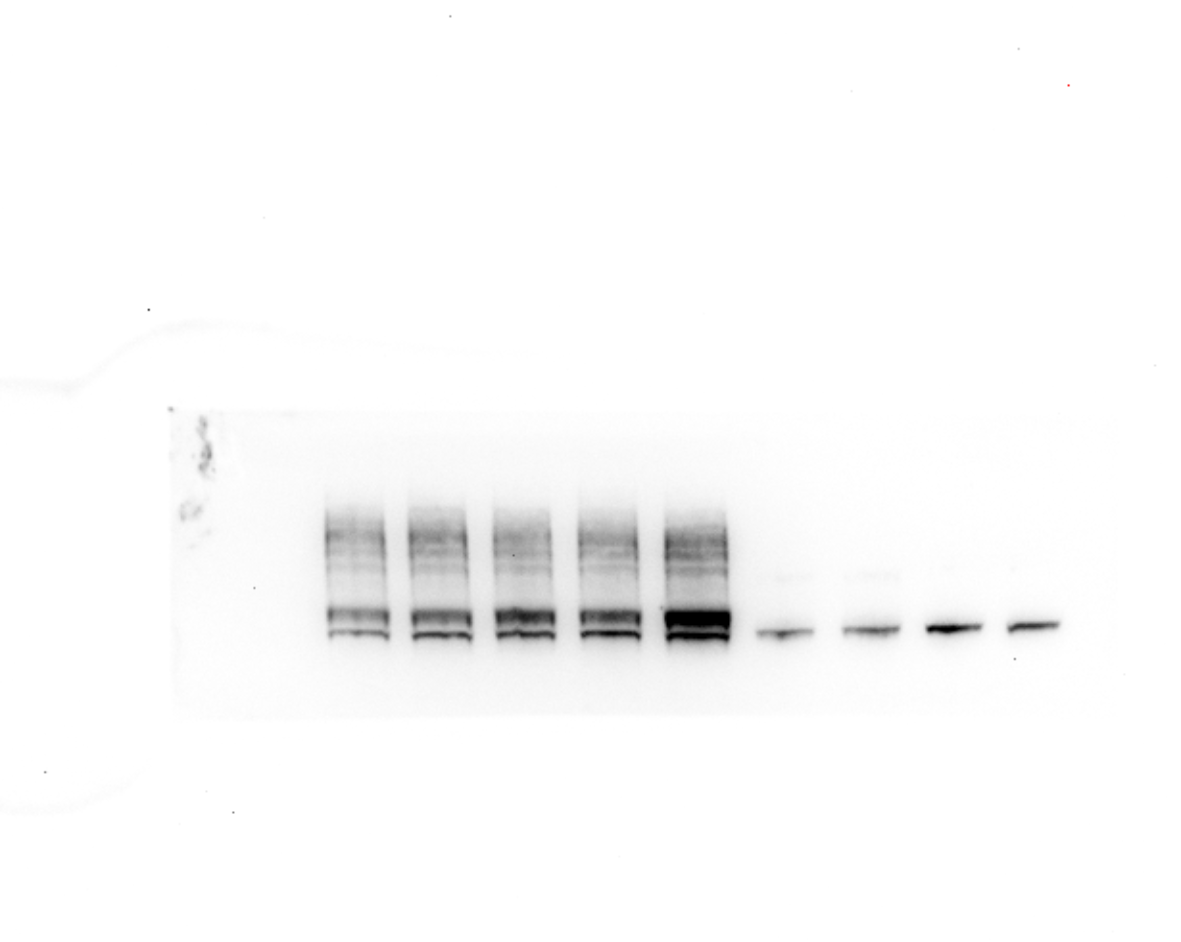

Supplement: Figure 3—figure supplement 1—source data 1. [file elife-84280-fig3-figsupp1-data1.zip › Fig 3 - fig S1 - Source data 1 - Unedited blots/Figure 3 - figure supplement 1D-E/PC070g HIF1a/4topChemi.tif]

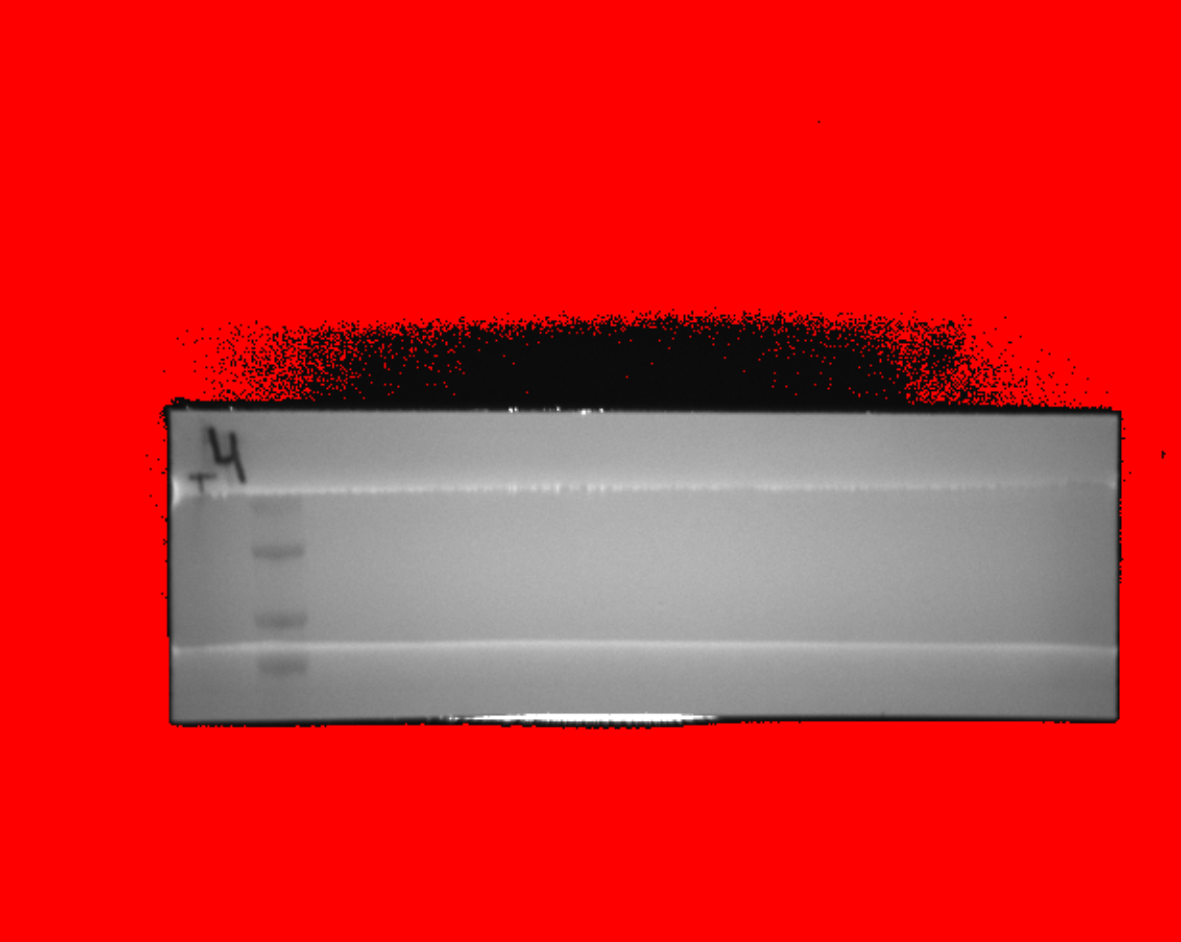

Supplement: Figure 3—figure supplement 1—source data 1. [file elife-84280-fig3-figsupp1-data1.zip › Fig 3 - fig S1 - Source data 1 - Unedited blots/Figure 3 - figure supplement 1D-E/PC070g HIF1a/4topMembrane.tif]

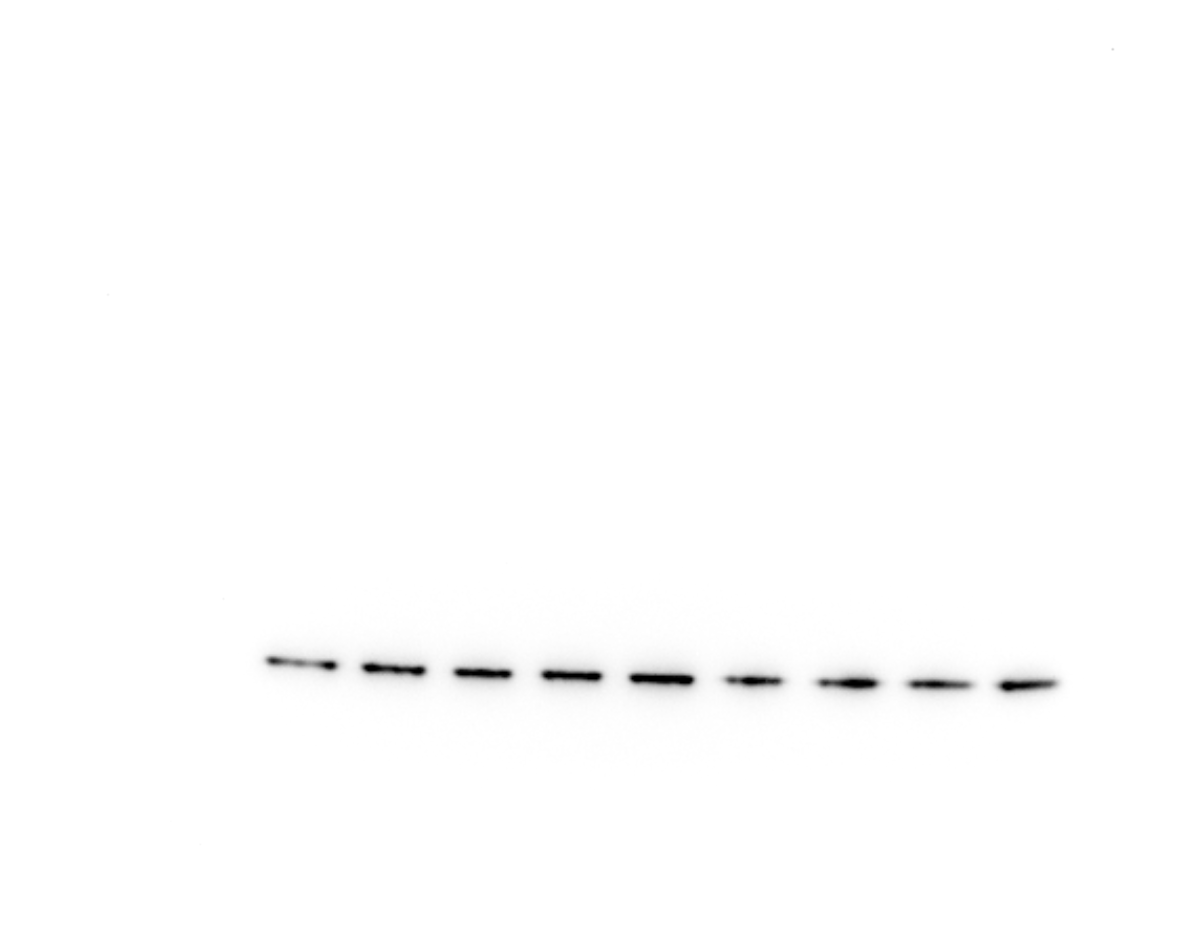

Supplement: Figure 3—figure supplement 1—source data 1. [file elife-84280-fig3-figsupp1-data1.zip › Fig 3 - fig S1 - Source data 1 - Unedited blots/Figure 3 - figure supplement 1D-E/PC070g PPIB/4PPIBChemi.tif]

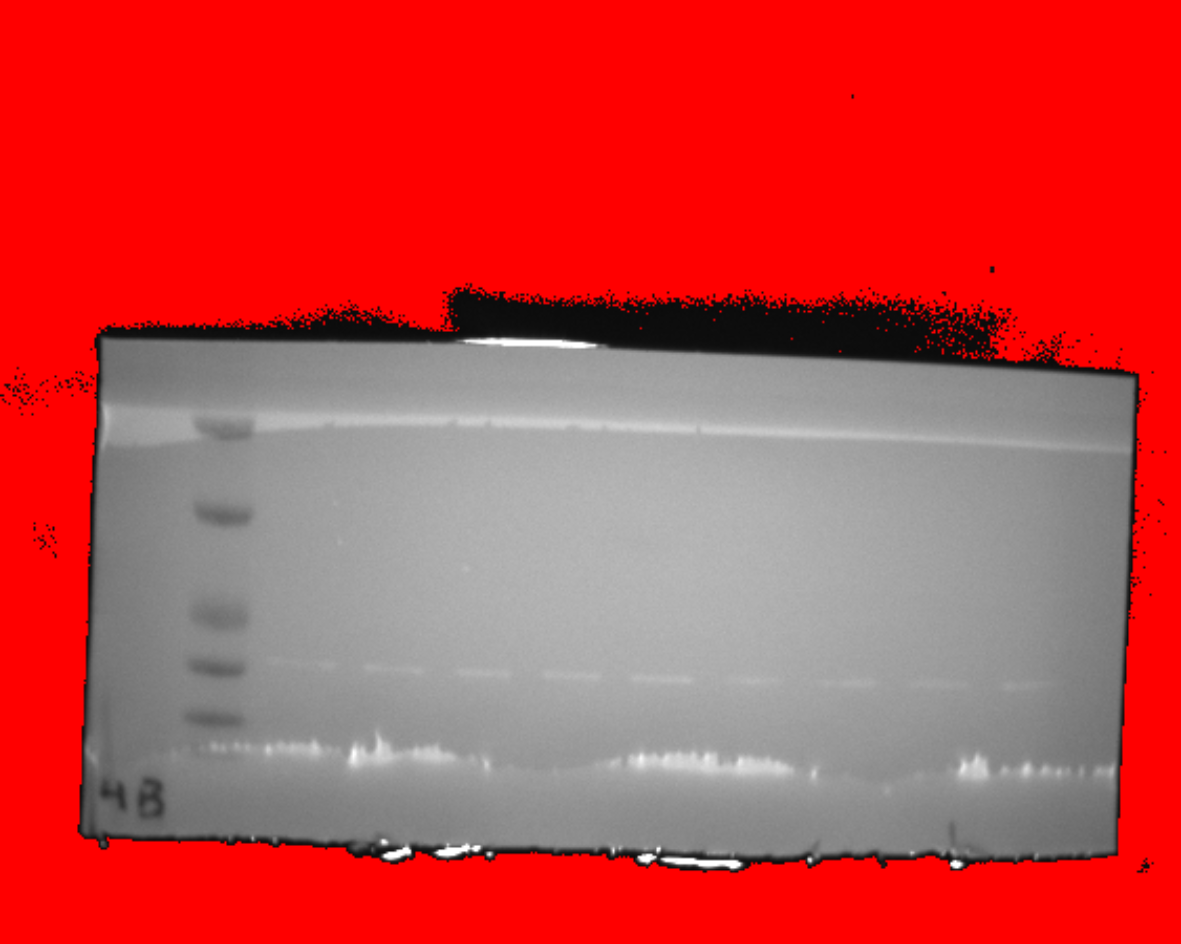

Supplement: Figure 3—figure supplement 1—source data 1. [file elife-84280-fig3-figsupp1-data1.zip › Fig 3 - fig S1 - Source data 1 - Unedited blots/Figure 3 - figure supplement 1D-E/PC070g PPIB/4PPIBMembrane.tif]

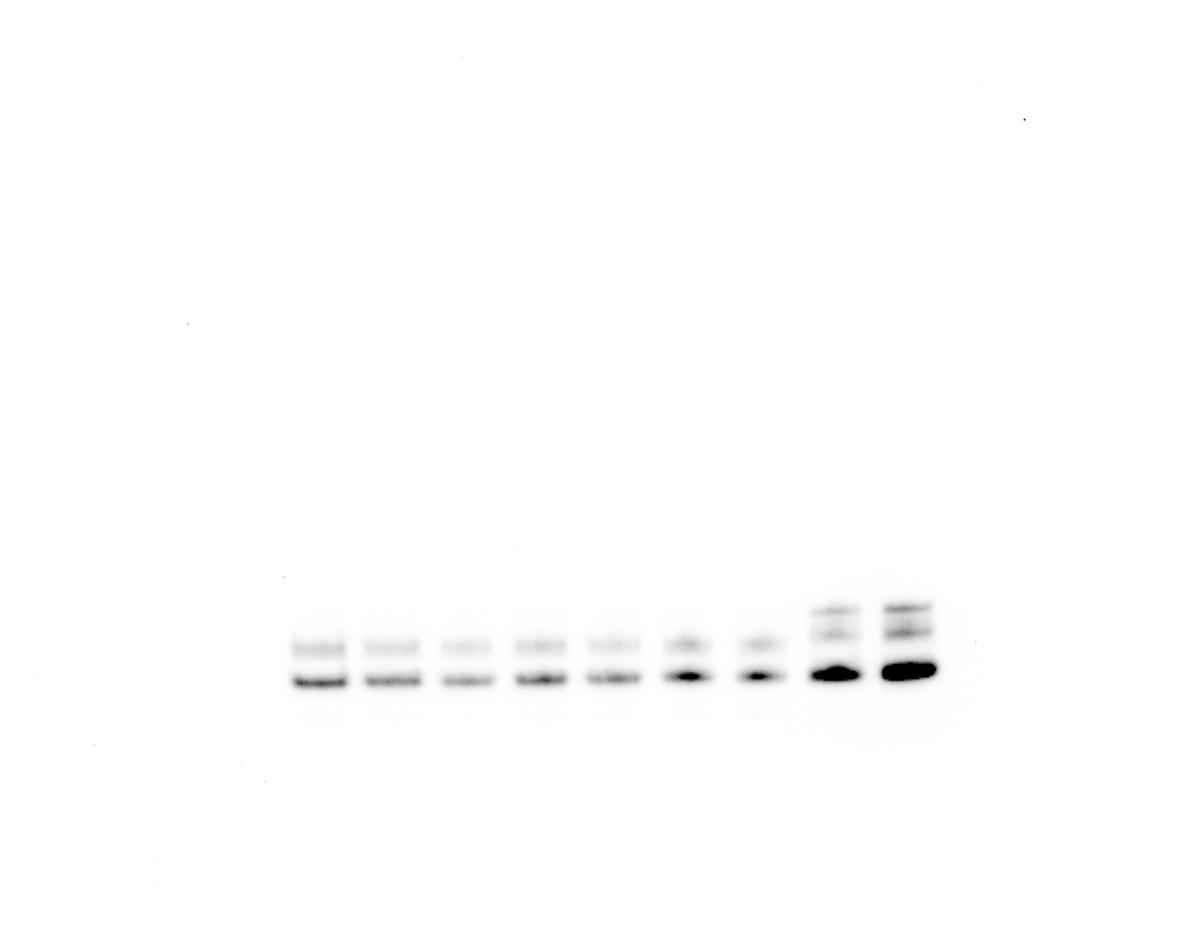

Supplement: Figure 3—figure supplement 1—source data 1. [file elife-84280-fig3-figsupp1-data1.zip › Fig 3 - fig S1 - Source data 1 - Unedited blots/Figure 3 - figure supplement 1D-E/PC070g VHL/3VHLChemi.tif]

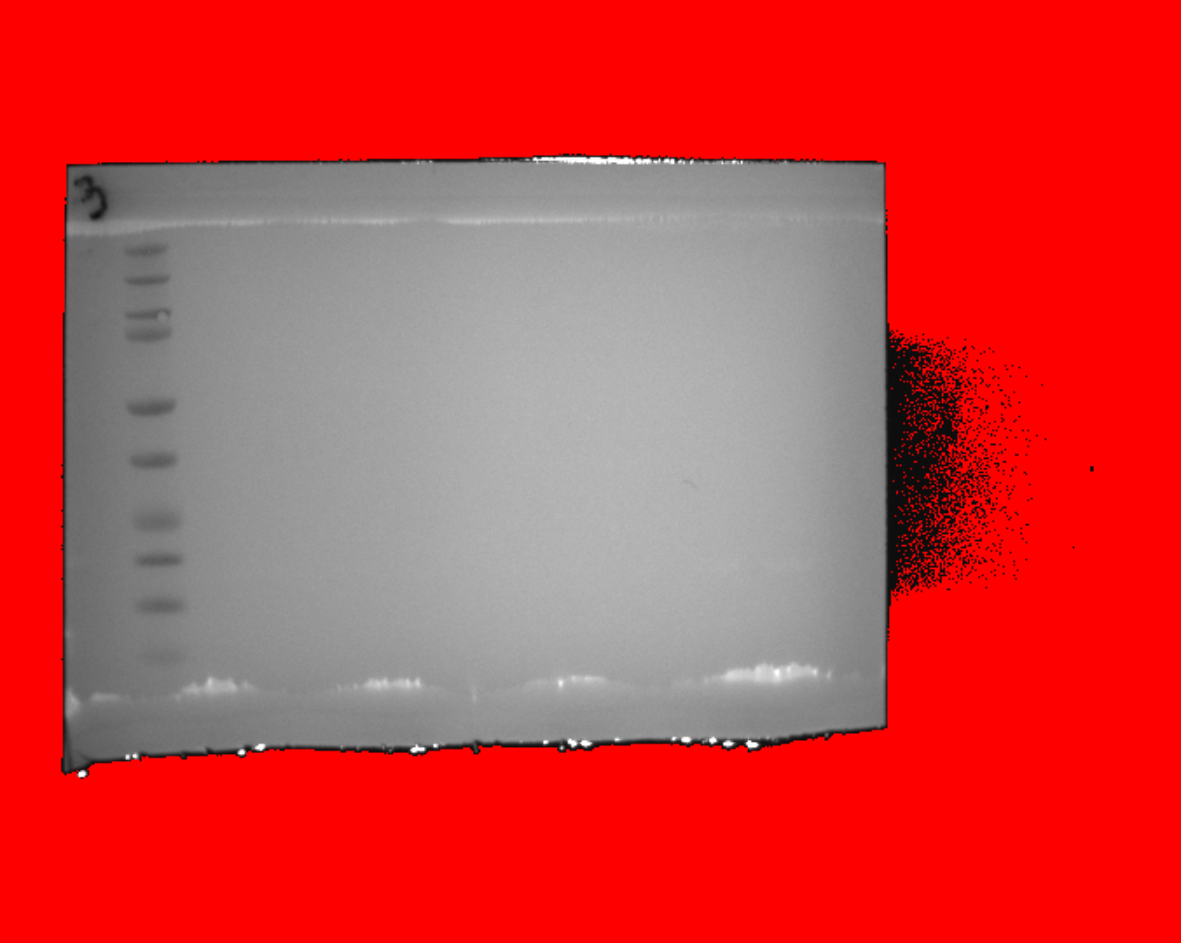

Supplement: Figure 3—figure supplement 1—source data 1. [file elife-84280-fig3-figsupp1-data1.zip › Fig 3 - fig S1 - Source data 1 - Unedited blots/Figure 3 - figure supplement 1D-E/PC070g VHL/3VHLMembrane.tif]

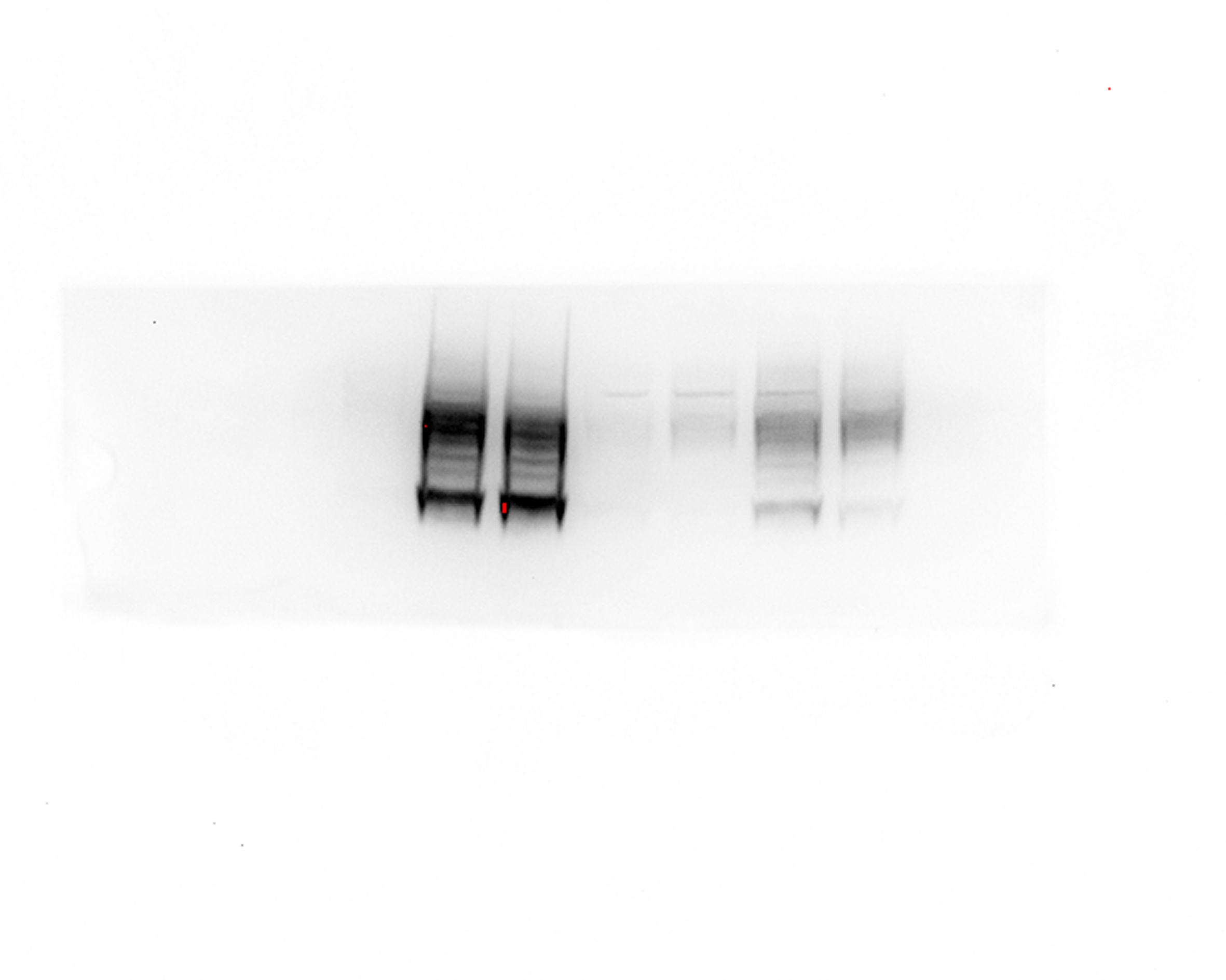

Supplement: Figure 4—figure supplement 1—source data 1. [file elife-84280-fig4-figsupp1-data1.zip › Fig 4 - fig S1 - Source data - Unedited blots/Figure 4 - figure supplement 1G/n1/HIF_Dn1_1/HIF_Dn1_1.jpg]

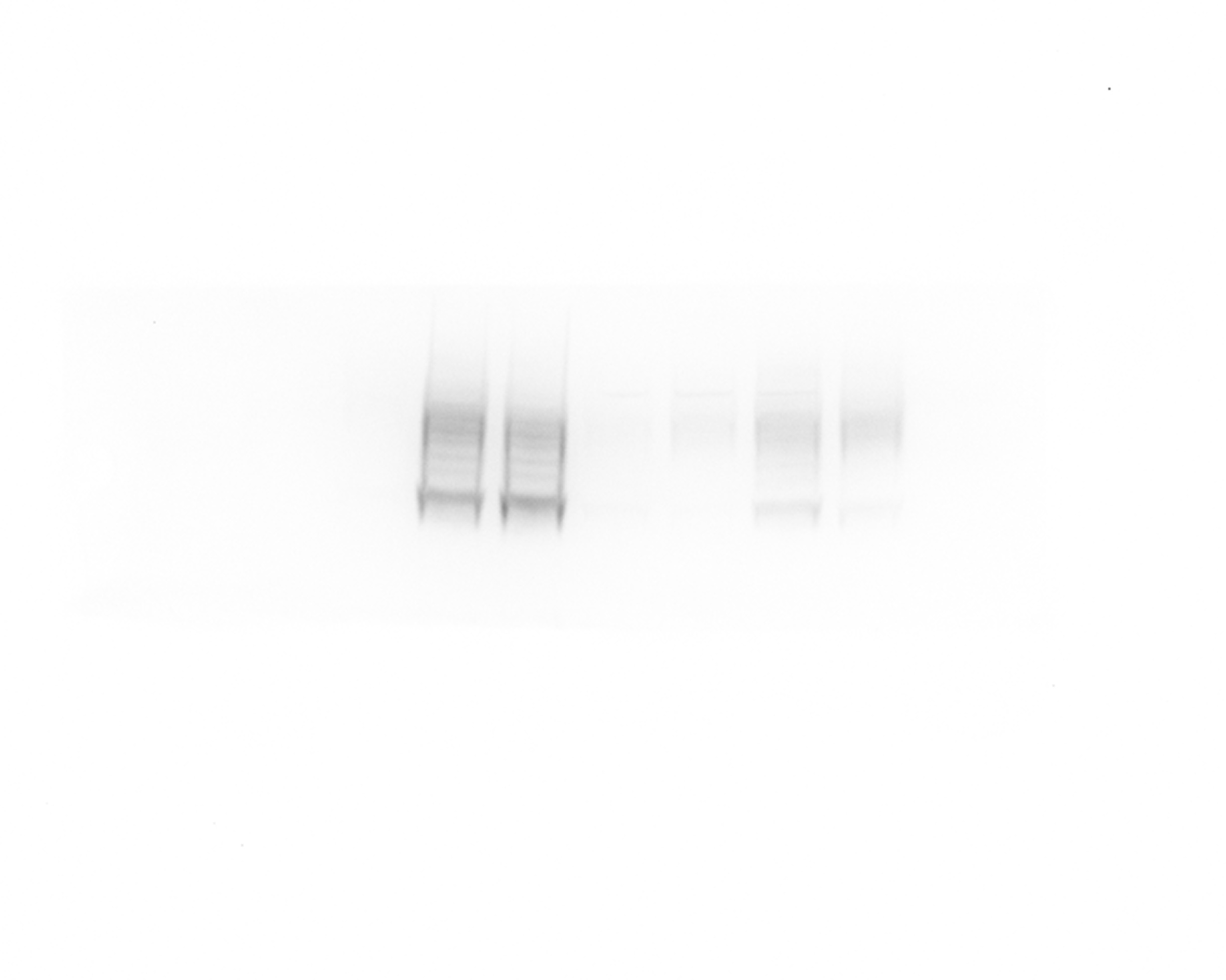

Supplement: Figure 4—figure supplement 1—source data 1. [file elife-84280-fig4-figsupp1-data1.zip › Fig 4 - fig S1 - Source data - Unedited blots/Figure 4 - figure supplement 1G/n1/HIF_Dn1_2/HIF_Dn1_2.jpg]

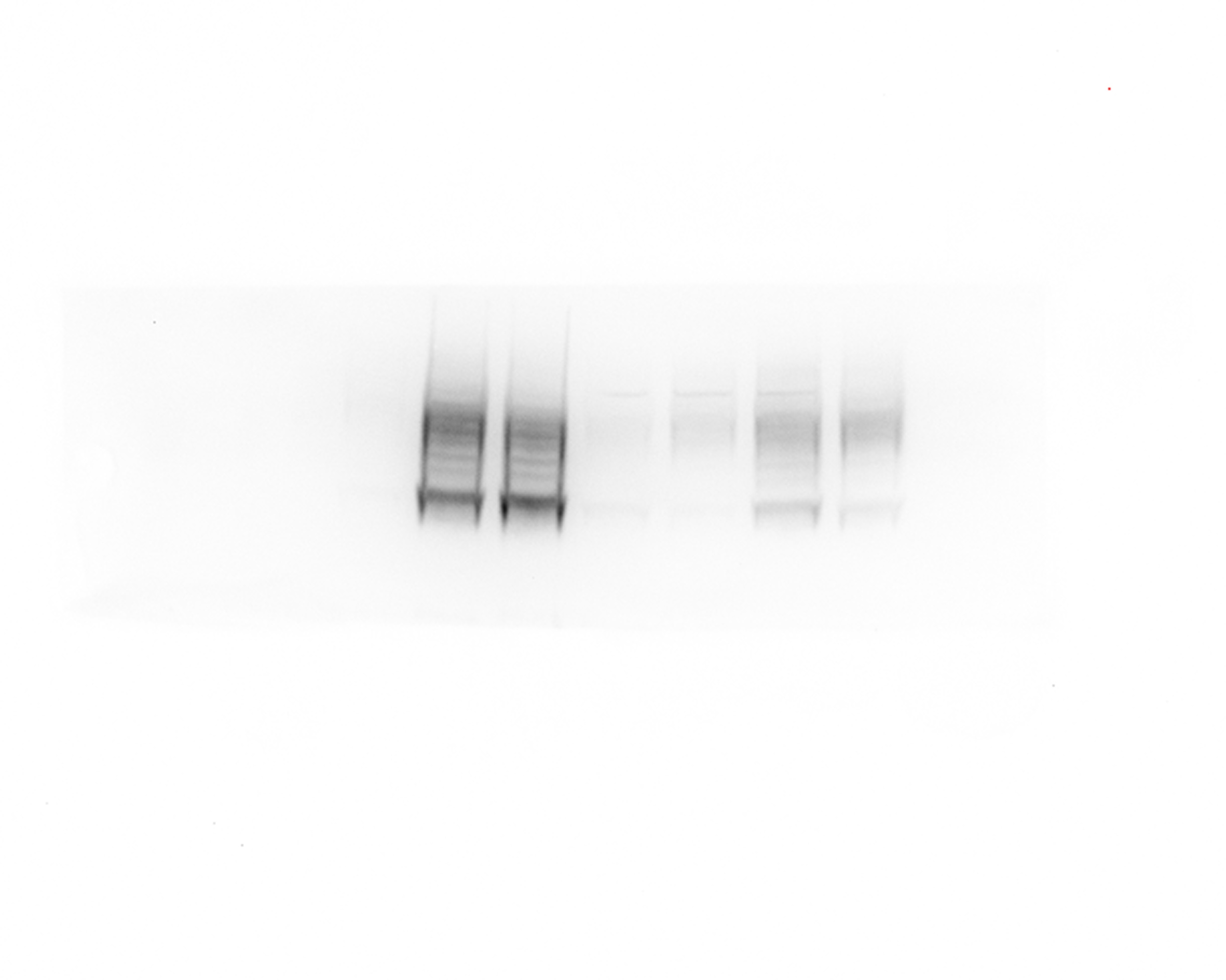

Supplement: Figure 4—figure supplement 1—source data 1. [file elife-84280-fig4-figsupp1-data1.zip › Fig 4 - fig S1 - Source data - Unedited blots/Figure 4 - figure supplement 1G/n1/HIF_Dn1_3/HIF_Dn1_3.jpg]

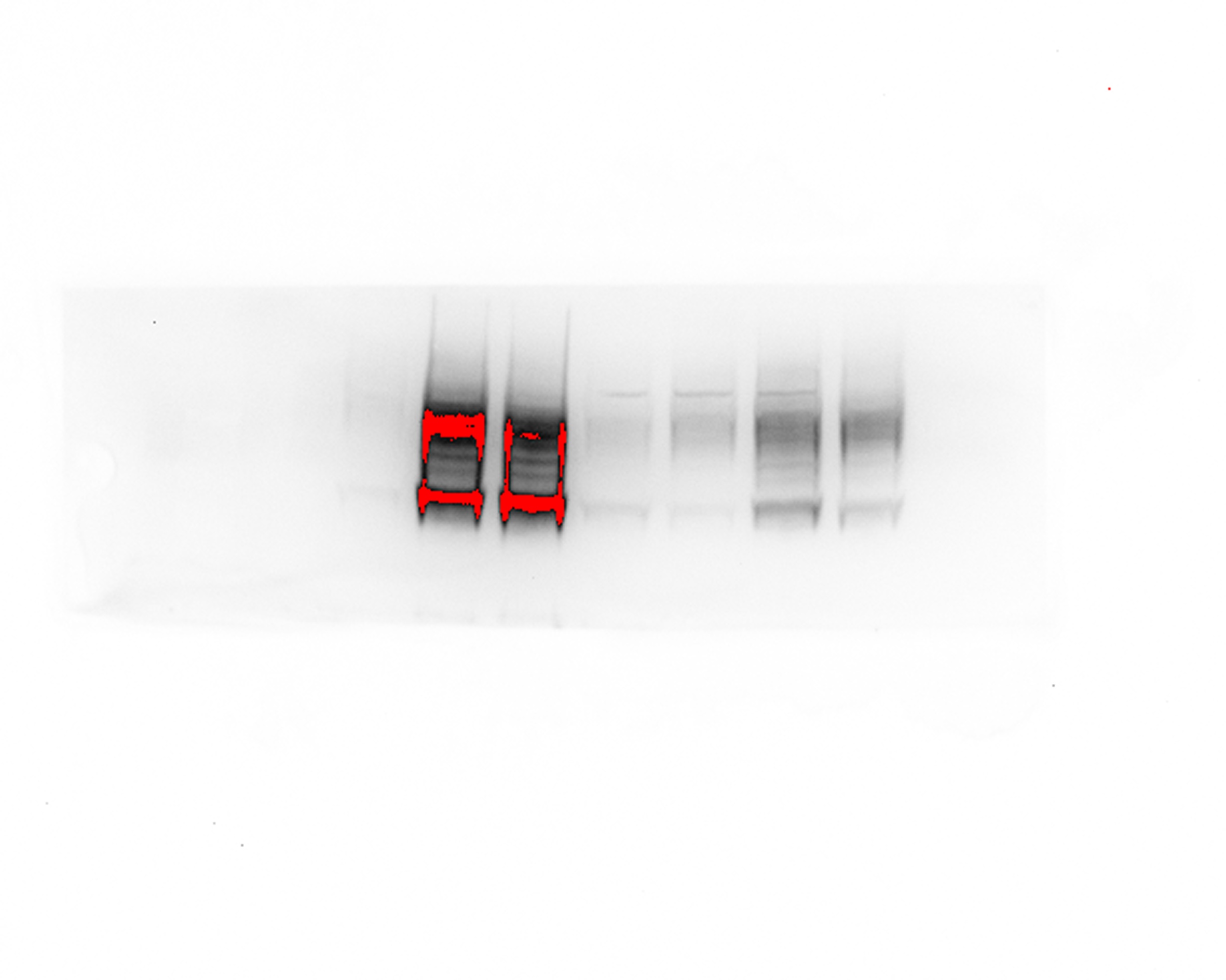

Supplement: Figure 4—figure supplement 1—source data 1. [file elife-84280-fig4-figsupp1-data1.zip › Fig 4 - fig S1 - Source data - Unedited blots/Figure 4 - figure supplement 1G/n1/HIF_Dn1_4/HIF_Dn1_4.jpg]

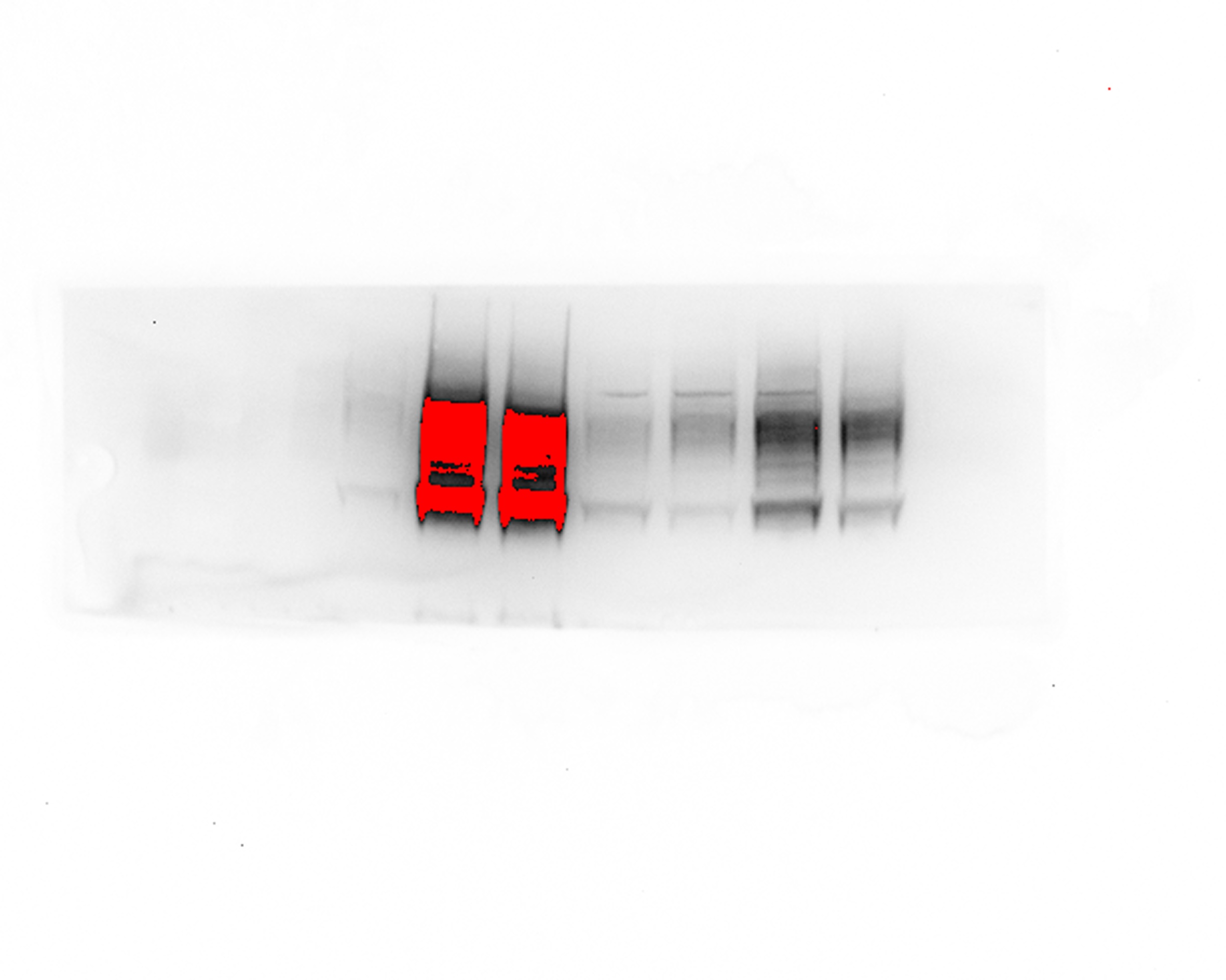

Supplement: Figure 4—figure supplement 1—source data 1. [file elife-84280-fig4-figsupp1-data1.zip › Fig 4 - fig S1 - Source data - Unedited blots/Figure 4 - figure supplement 1G/n1/HIF_Dn1_5/HIF_Dn1_5.jpg]

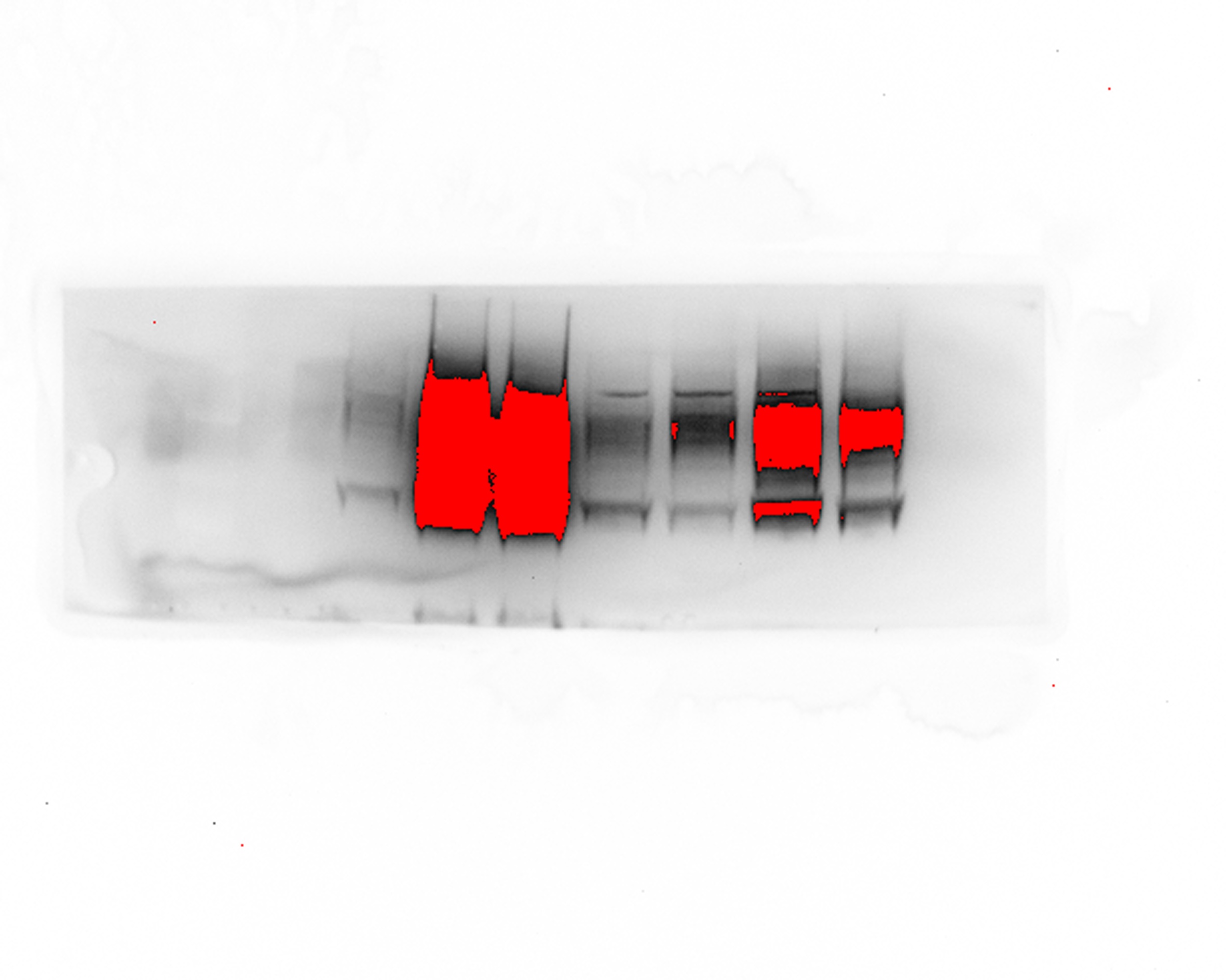

Supplement: Figure 4—figure supplement 1—source data 1. [file elife-84280-fig4-figsupp1-data1.zip › Fig 4 - fig S1 - Source data - Unedited blots/Figure 4 - figure supplement 1G/n1/HIF_DN1_6/HIF_DN1_6.jpg]

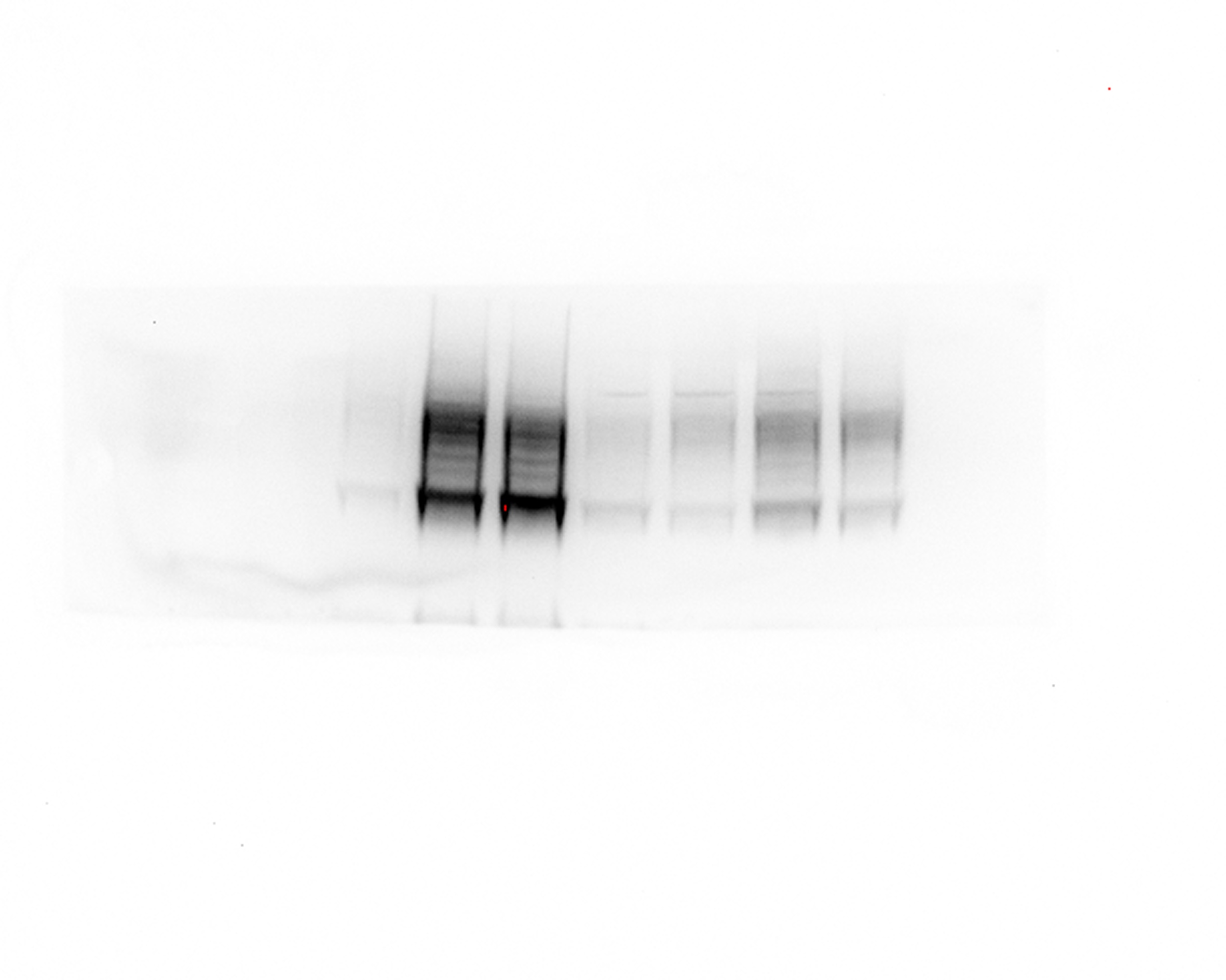

Supplement: Figure 4—figure supplement 1—source data 1. [file elife-84280-fig4-figsupp1-data1.zip › Fig 4 - fig S1 - Source data - Unedited blots/Figure 4 - figure supplement 1G/n1/HIF_Dn1_7/HIF_Dn1_7.jpg]

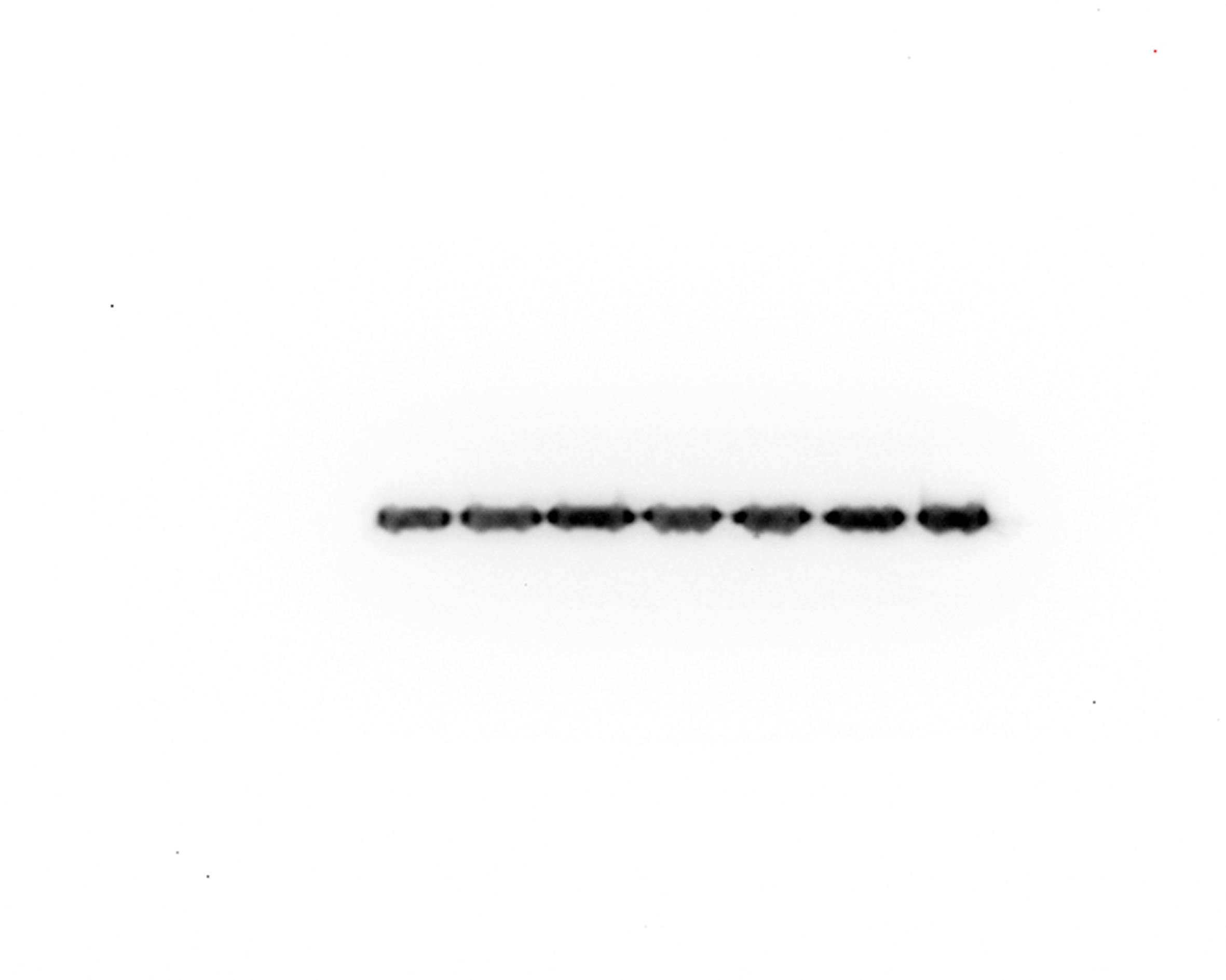

Supplement: Figure 4—figure supplement 1—source data 1. [file elife-84280-fig4-figsupp1-data1.zip › Fig 4 - fig S1 - Source data - Unedited blots/Figure 4 - figure supplement 1G/n1/PPIB_Dn1/CYCLO_Dn1.jpg]

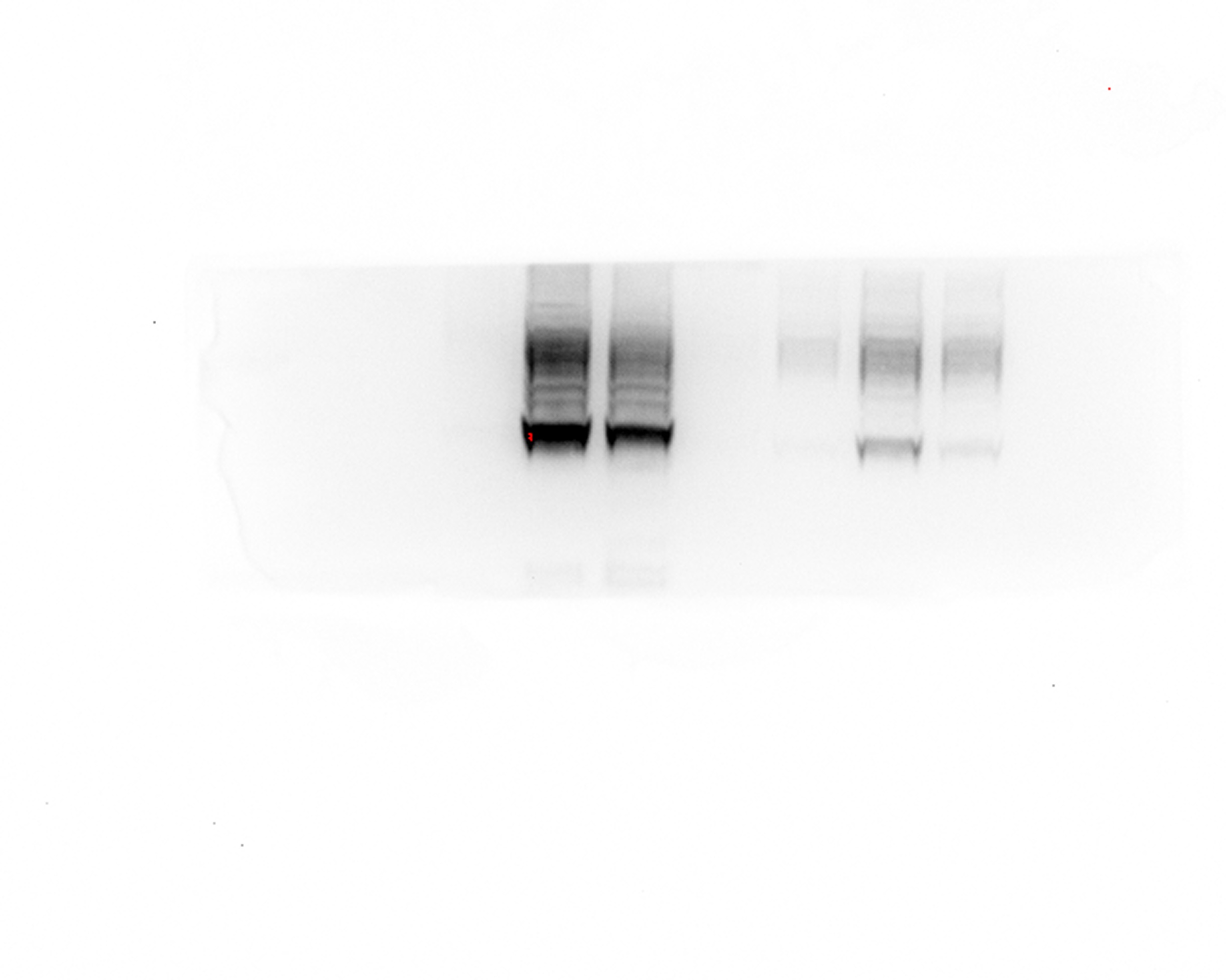

Supplement: Figure 4—figure supplement 1—source data 1. [file elife-84280-fig4-figsupp1-data1.zip › Fig 4 - fig S1 - Source data - Unedited blots/Figure 4 - figure supplement 1G/n2/HIF_Dn2_1/HIF_Dn2_1.jpg]

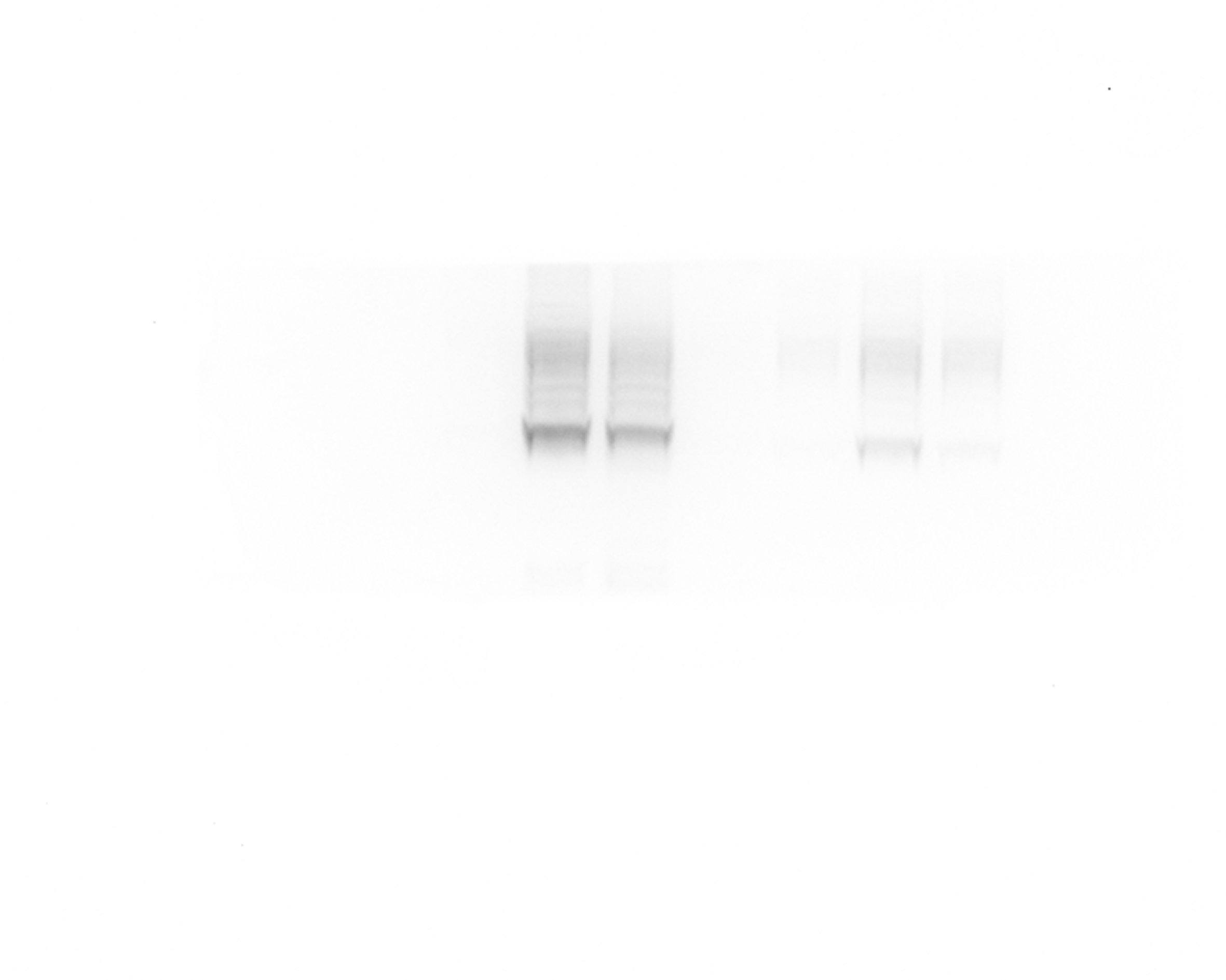

Supplement: Figure 4—figure supplement 1—source data 1. [file elife-84280-fig4-figsupp1-data1.zip › Fig 4 - fig S1 - Source data - Unedited blots/Figure 4 - figure supplement 1G/n2/HIF_Dn2_2/HIF_Dn2_2.jpg]

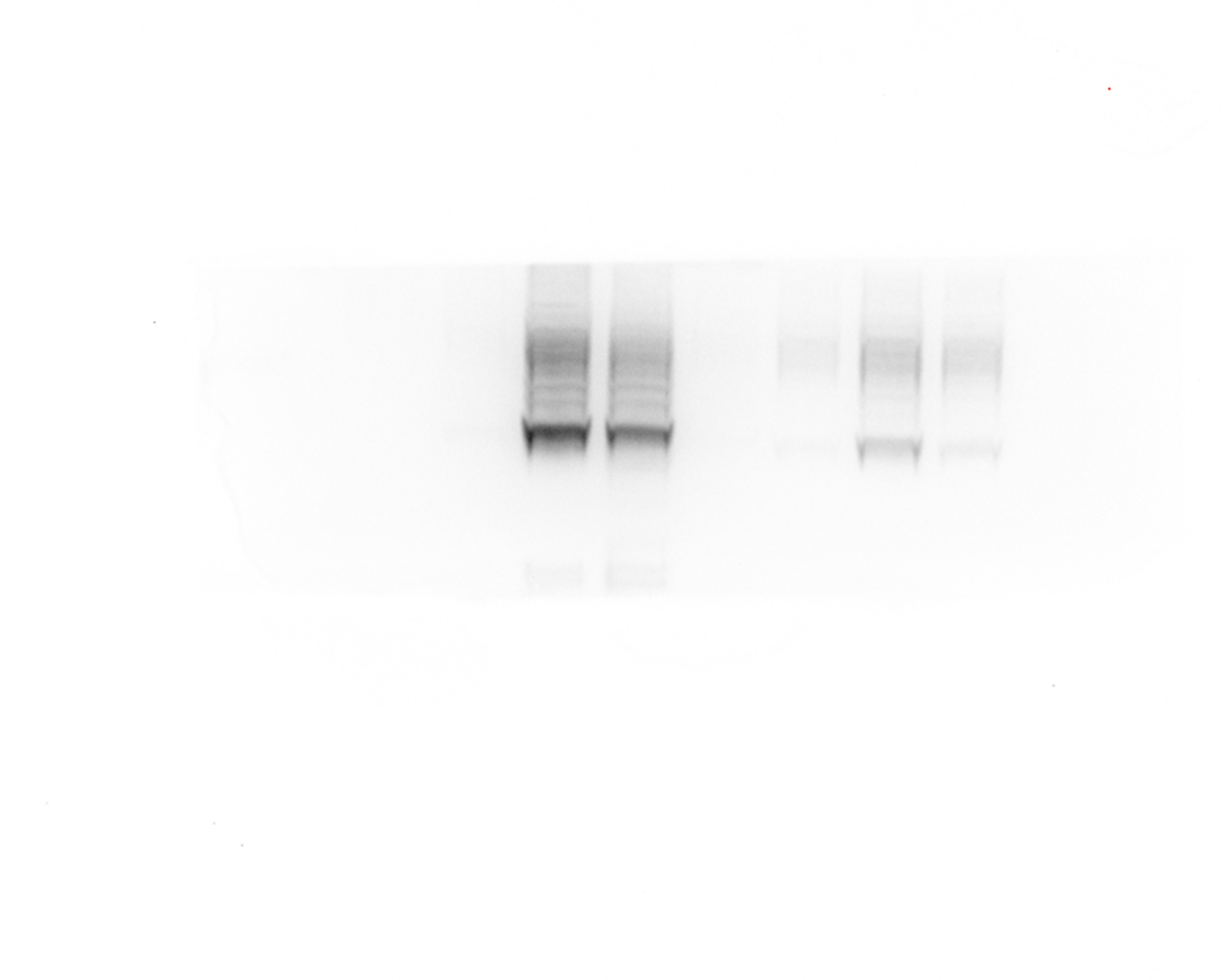

Supplement: Figure 4—figure supplement 1—source data 1. [file elife-84280-fig4-figsupp1-data1.zip › Fig 4 - fig S1 - Source data - Unedited blots/Figure 4 - figure supplement 1G/n2/HIF_Dn2_3/HIF_Dn2_3.jpg]

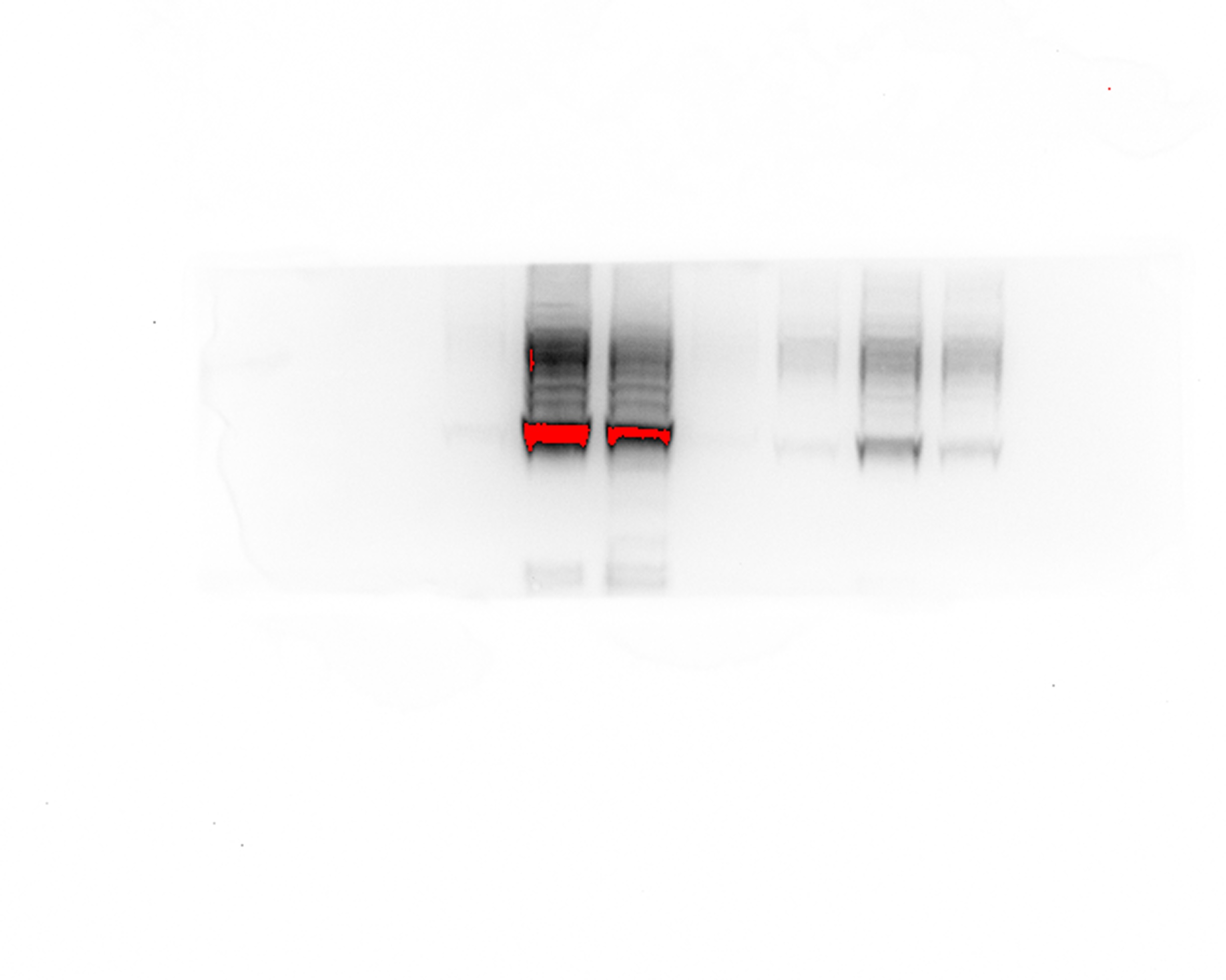

Supplement: Figure 4—figure supplement 1—source data 1. [file elife-84280-fig4-figsupp1-data1.zip › Fig 4 - fig S1 - Source data - Unedited blots/Figure 4 - figure supplement 1G/n2/HIF_Dn2_4/HIF_Dn2_4.jpg]

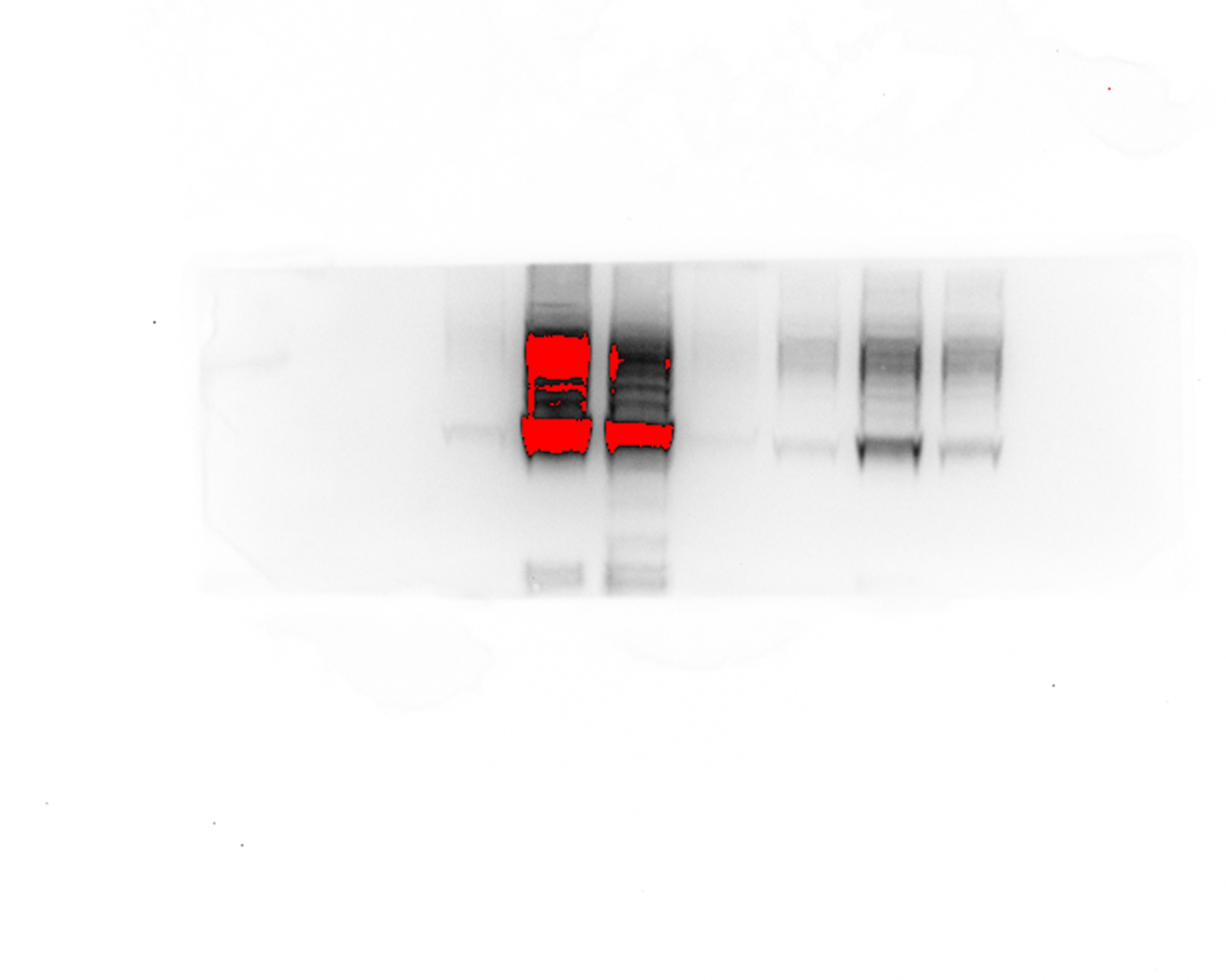

Supplement: Figure 4—figure supplement 1—source data 1. [file elife-84280-fig4-figsupp1-data1.zip › Fig 4 - fig S1 - Source data - Unedited blots/Figure 4 - figure supplement 1G/n2/HIF_Dn2_5/HIF_Dn2_5.jpg]

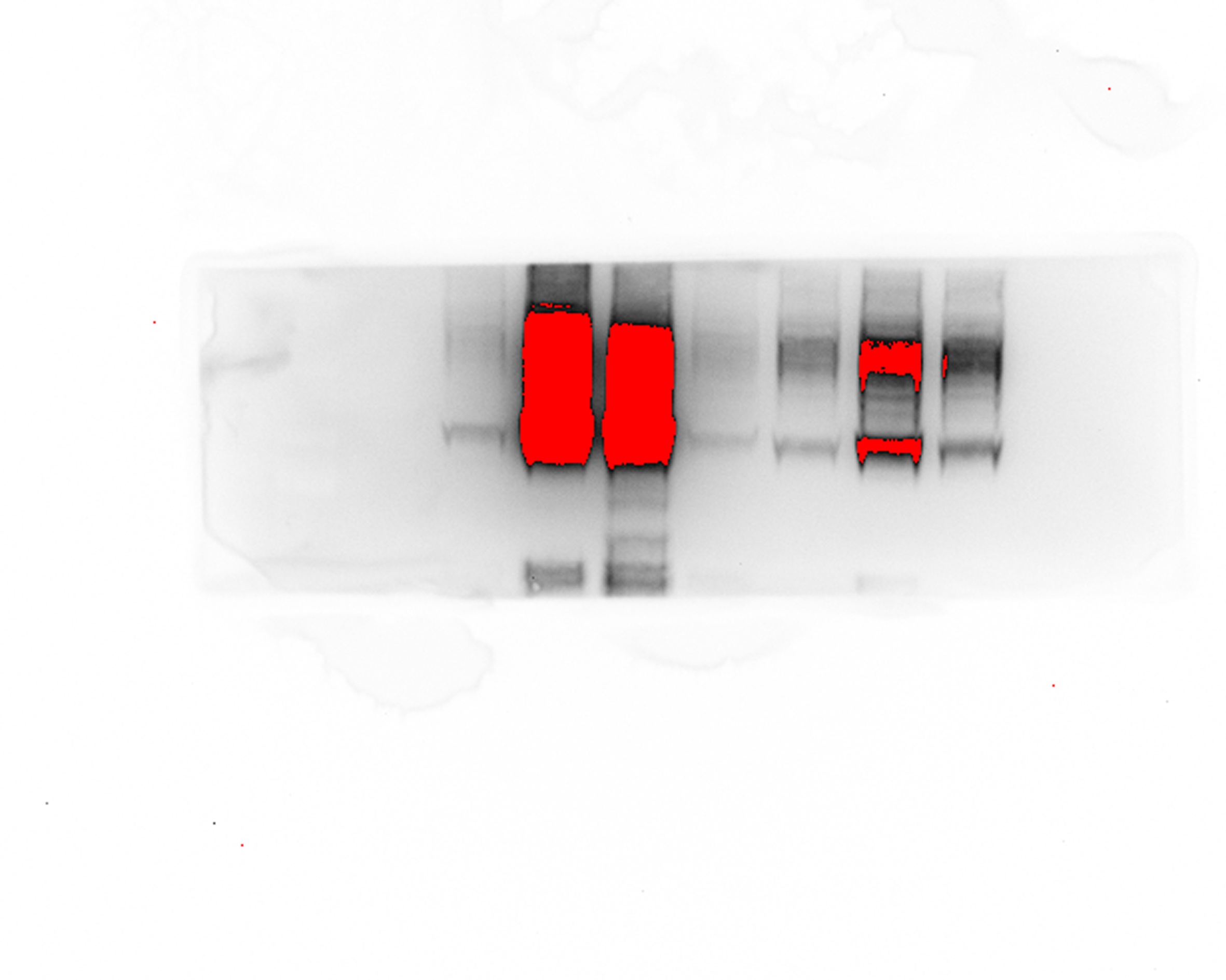

Supplement: Figure 4—figure supplement 1—source data 1. [file elife-84280-fig4-figsupp1-data1.zip › Fig 4 - fig S1 - Source data - Unedited blots/Figure 4 - figure supplement 1G/n2/HIF_Dn2_6/HIF_Dn2_6.jpg]

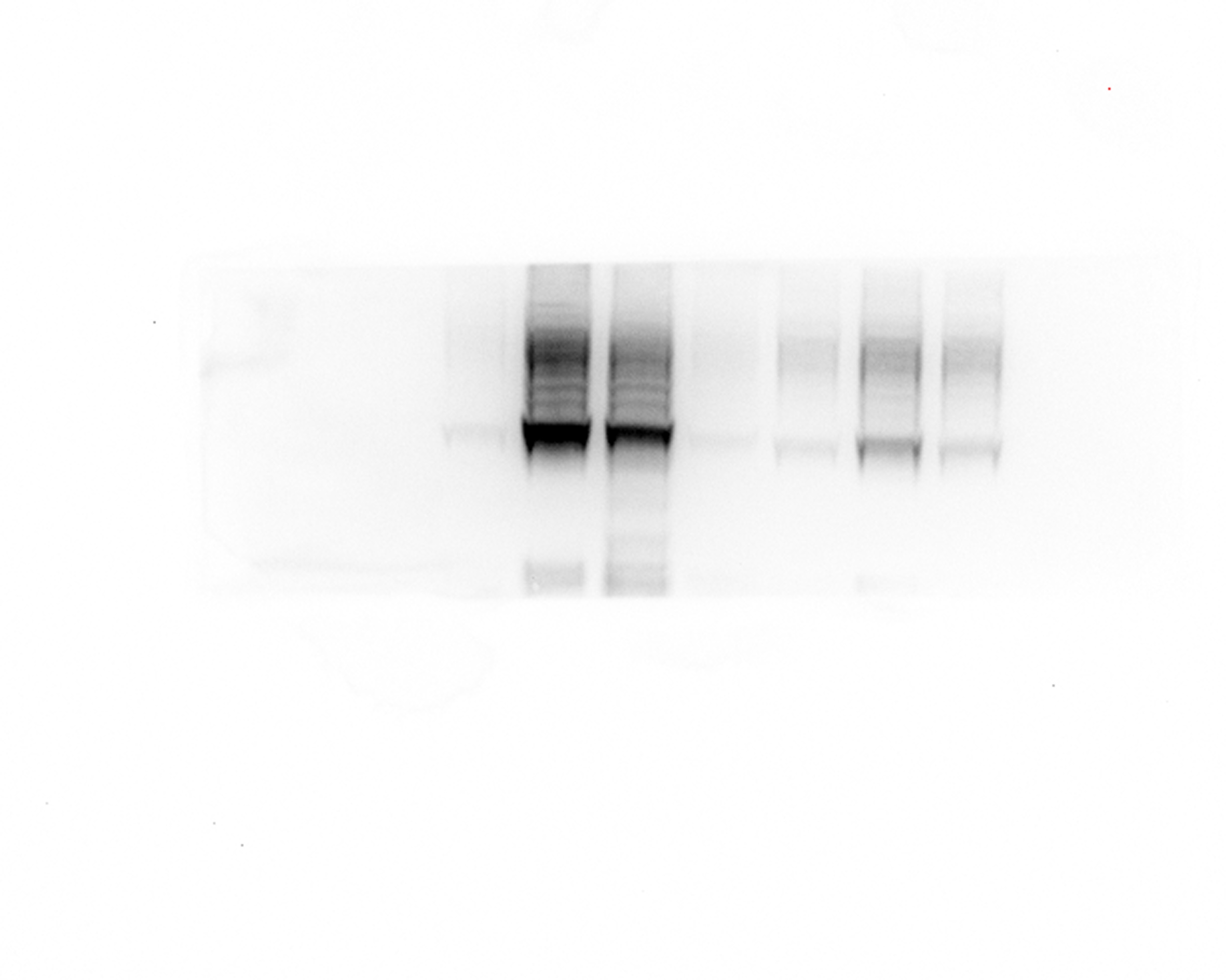

Supplement: Figure 4—figure supplement 1—source data 1. [file elife-84280-fig4-figsupp1-data1.zip › Fig 4 - fig S1 - Source data - Unedited blots/Figure 4 - figure supplement 1G/n2/HIF_Dn2_7/HIF_Dn2_7.jpg]

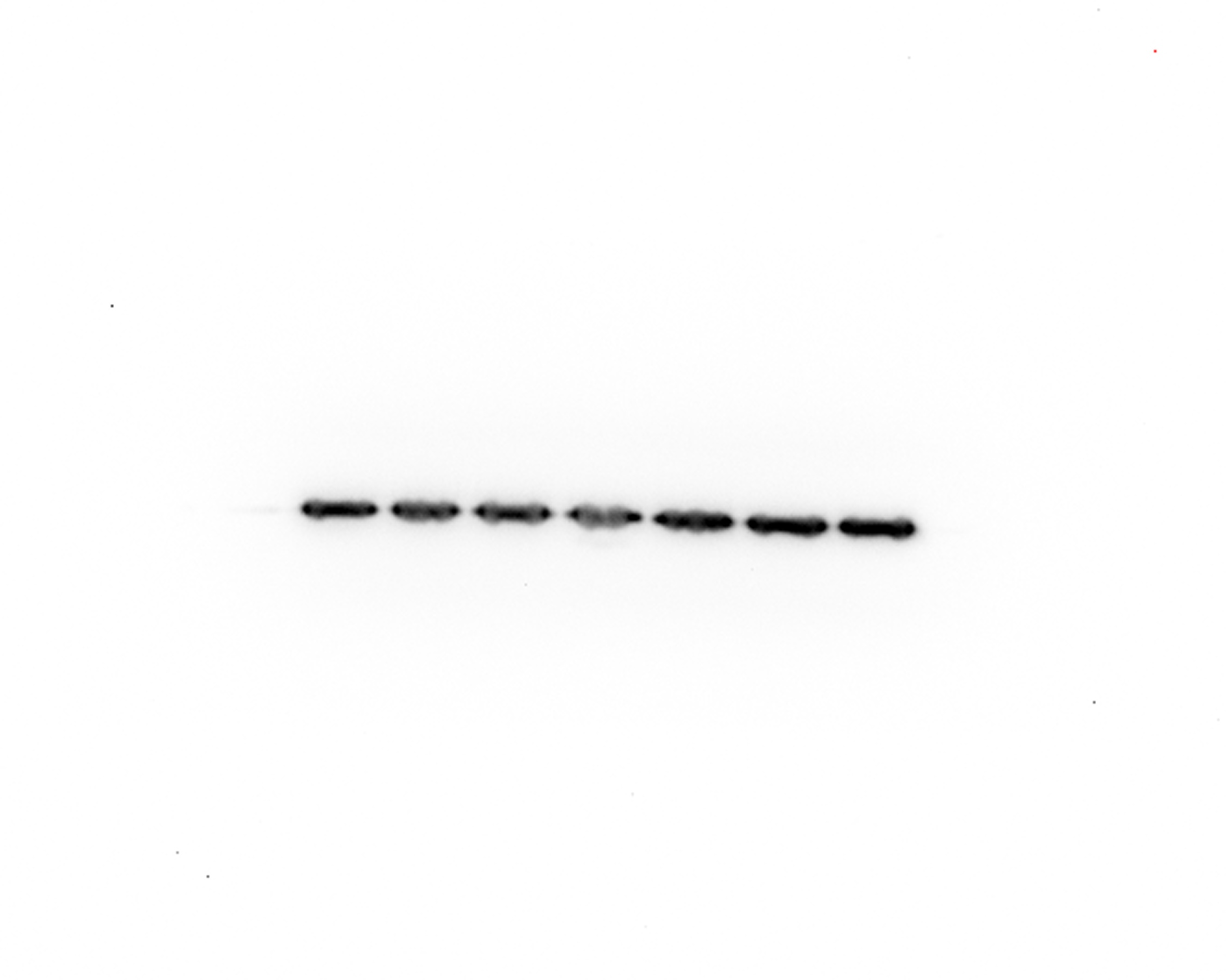

Supplement: Figure 4—figure supplement 1—source data 1. [file elife-84280-fig4-figsupp1-data1.zip › Fig 4 - fig S1 - Source data - Unedited blots/Figure 4 - figure supplement 1G/n2/PPIB_Dn2/CYCLO_Dn2.jpg]

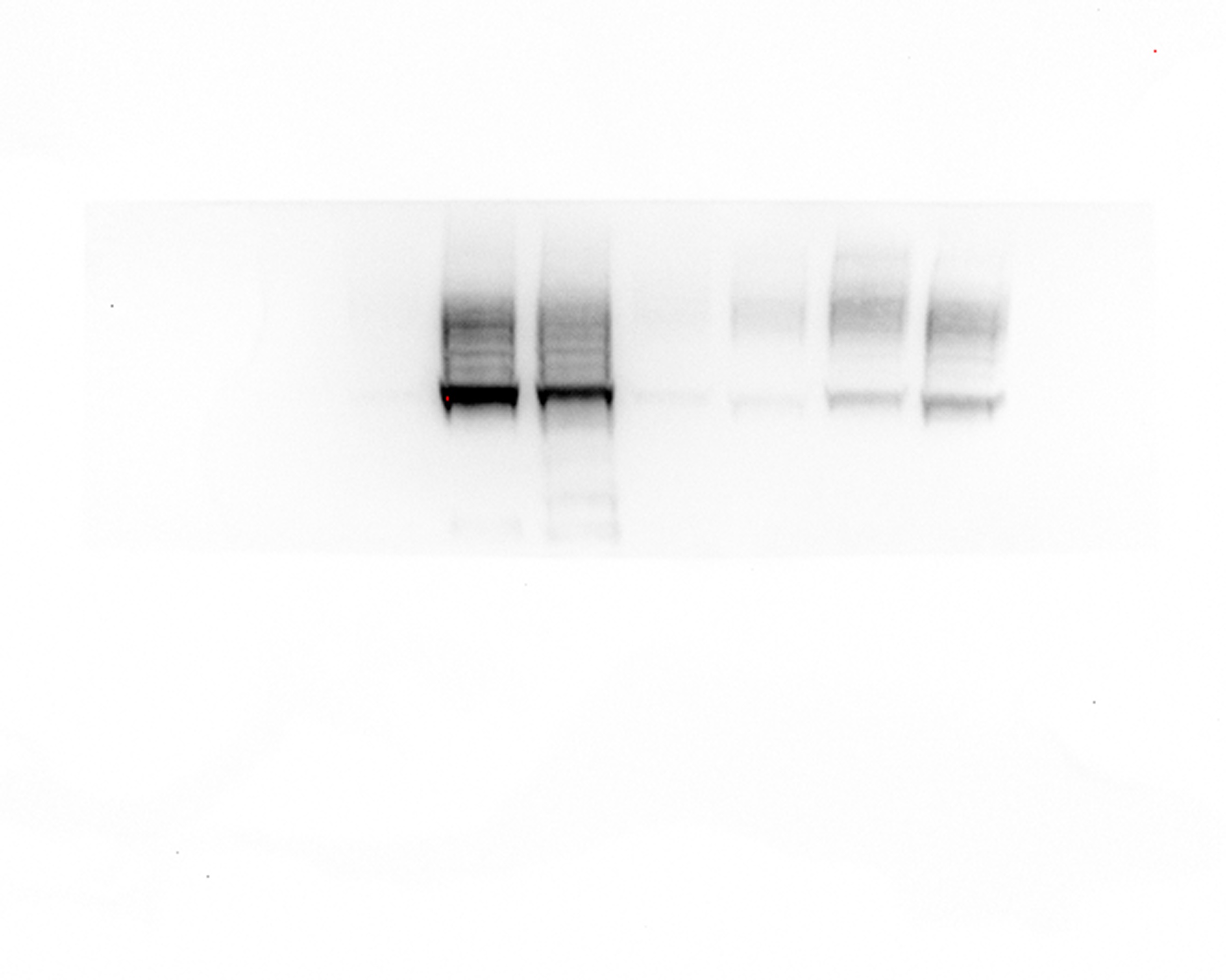

Supplement: Figure 4—figure supplement 1—source data 1. [file elife-84280-fig4-figsupp1-data1.zip › Fig 4 - fig S1 - Source data - Unedited blots/Figure 4 - figure supplement 1G/n3/HIF_Dn3_1/HIF_Dn3_1.jpg]

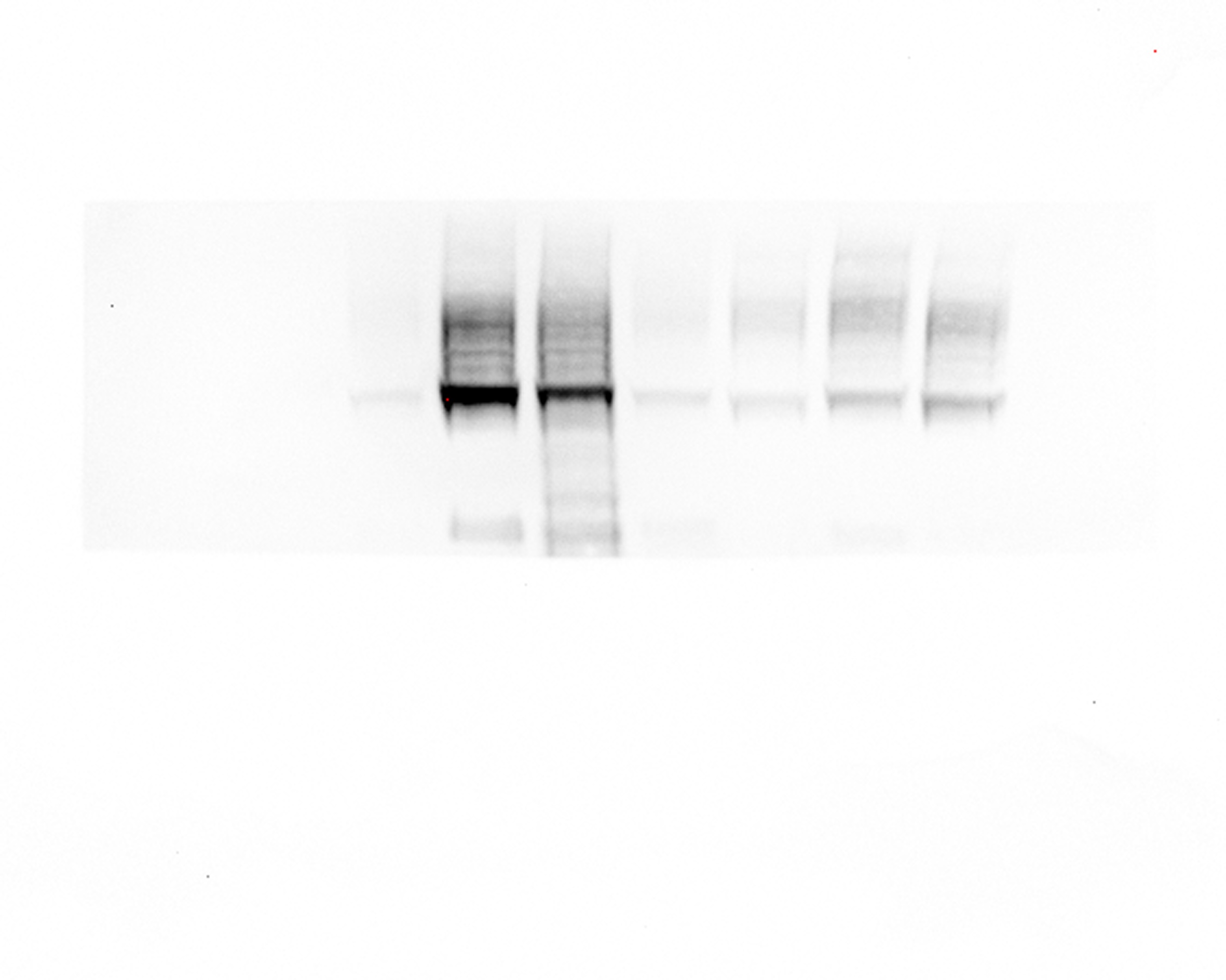

Supplement: Figure 4—figure supplement 1—source data 1. [file elife-84280-fig4-figsupp1-data1.zip › Fig 4 - fig S1 - Source data - Unedited blots/Figure 4 - figure supplement 1G/n3/HIF_Dn3_7/HIF_Dn3_7.jpg]

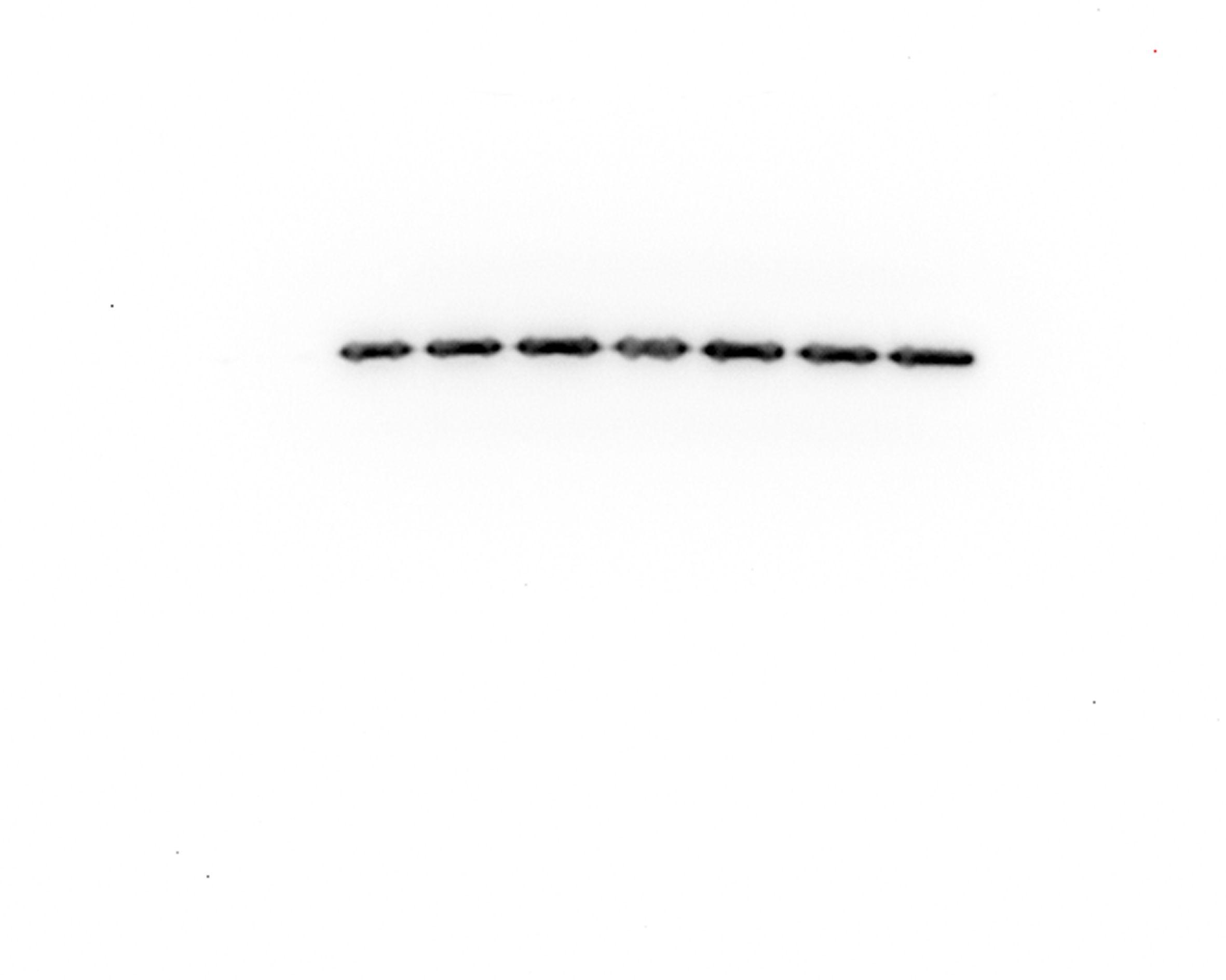

Supplement: Figure 4—figure supplement 1—source data 1. [file elife-84280-fig4-figsupp1-data1.zip › Fig 4 - fig S1 - Source data - Unedited blots/Figure 4 - figure supplement 1G/n3/PPIB_Dn3/CYCLO_Dn3.jpg]

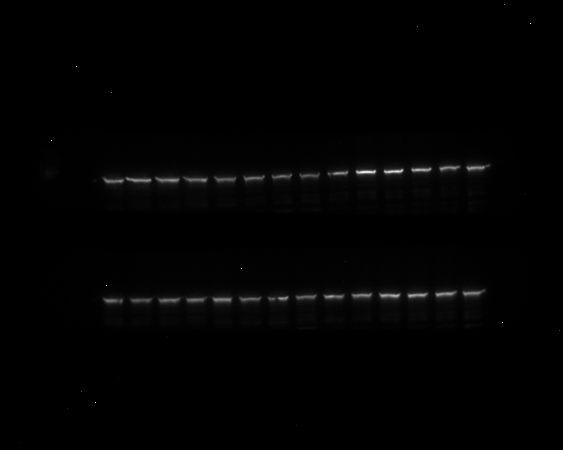

Supplement: Figure 5—source data 1. [file elife-84280-fig5-data1.zip › Figure 5 - Source data 1 - Unedited blots/n1-4 Vinculin (top)/CHEMI_07022022_020808_(Chemi)_raw.tif]

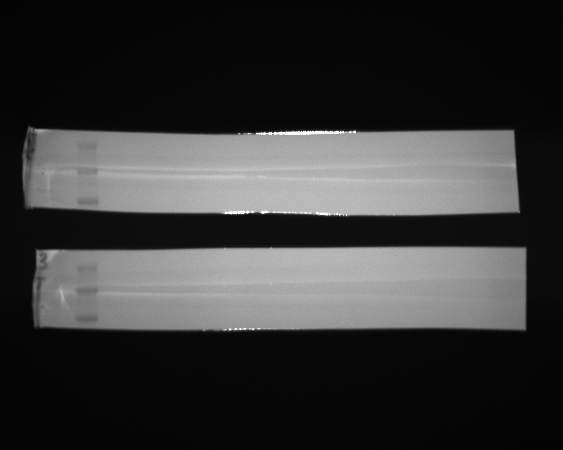

Supplement: Figure 5—source data 1. [file elife-84280-fig5-data1.zip › Figure 5 - Source data 1 - Unedited blots/n1-4 Vinculin (top)/CHEMI_07022022_020808_(Membrane)_raw.tif]

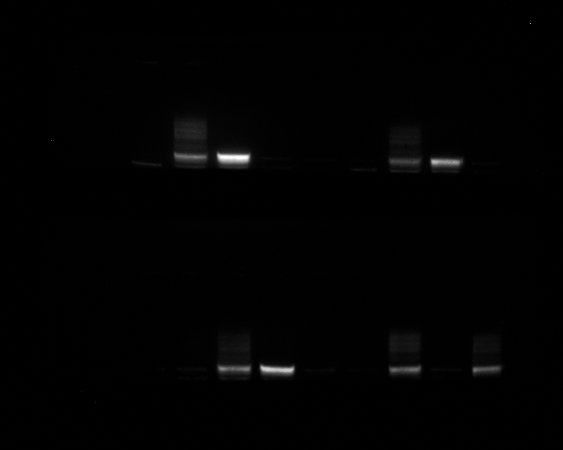

Supplement: Figure 5—source data 1. [file elife-84280-fig5-data1.zip › Figure 5 - Source data 1 - Unedited blots/n1-5 HIF1/CHEMI_07012022_195754_(Chemi)_raw.tif]

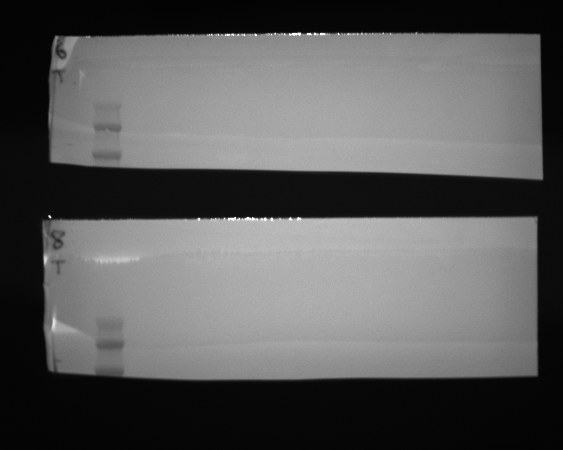

Supplement: Figure 5—source data 1. [file elife-84280-fig5-data1.zip › Figure 5 - Source data 1 - Unedited blots/n1-5 HIF1/CHEMI_07012022_195754_(Membrane)_raw.tif]

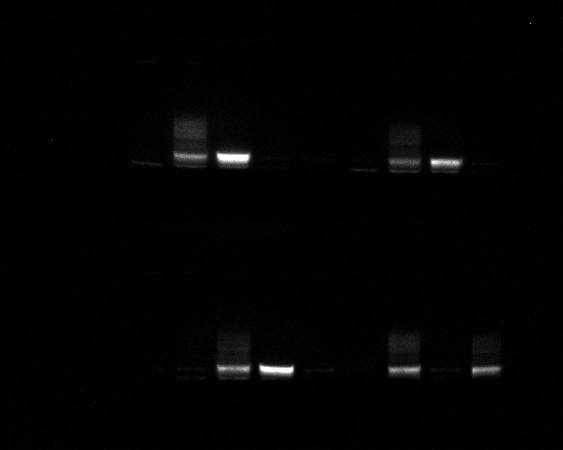

Supplement: Figure 5—source data 1. [file elife-84280-fig5-data1.zip › Figure 5 - Source data 1 - Unedited blots/n1-5 HIF1/CHEMI_07012022_195829_(Chemi)_raw.tif]

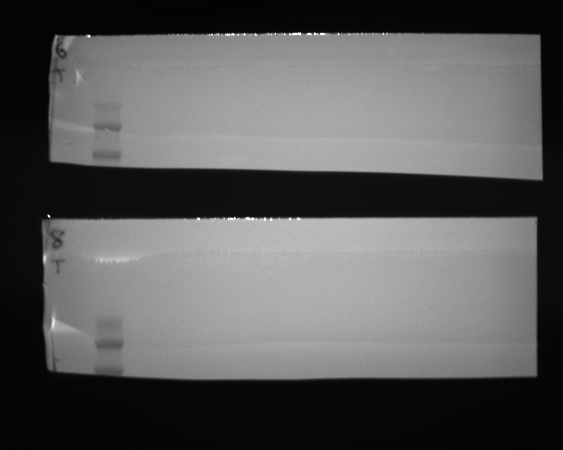

Supplement: Figure 5—source data 1. [file elife-84280-fig5-data1.zip › Figure 5 - Source data 1 - Unedited blots/n1-5 HIF1/CHEMI_07012022_195829_(Membrane)_raw.tif]

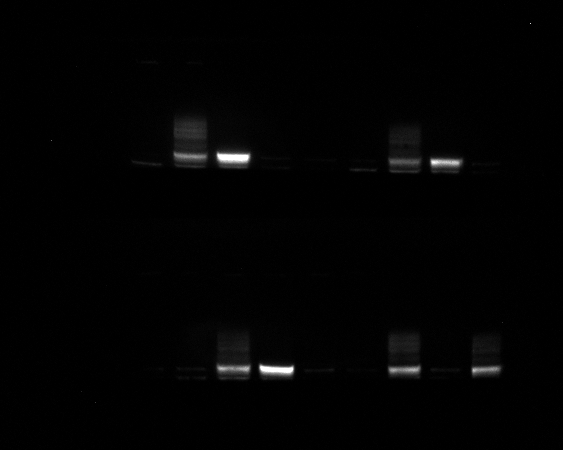

Supplement: Figure 5—source data 1. [file elife-84280-fig5-data1.zip › Figure 5 - Source data 1 - Unedited blots/n1-5 HIF1/CHEMI_07012022_195835_(Chemi)_raw.tif]

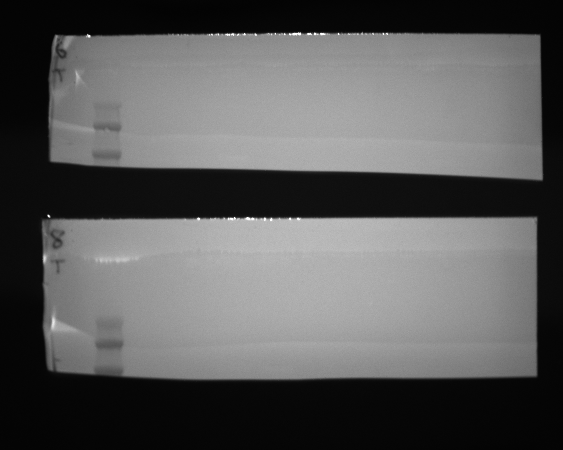

Supplement: Figure 5—source data 1. [file elife-84280-fig5-data1.zip › Figure 5 - Source data 1 - Unedited blots/n1-5 HIF1/CHEMI_07012022_195835_(Membrane)_raw.tif]

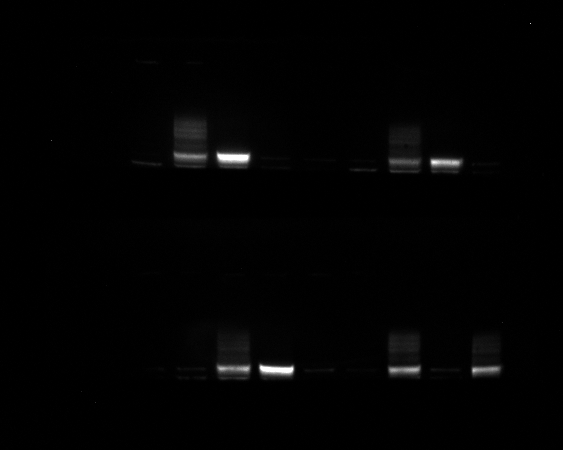

Supplement: Figure 5—source data 1. [file elife-84280-fig5-data1.zip › Figure 5 - Source data 1 - Unedited blots/n1-5 HIF1/CHEMI_07012022_195842_(Chemi)_raw.tif]

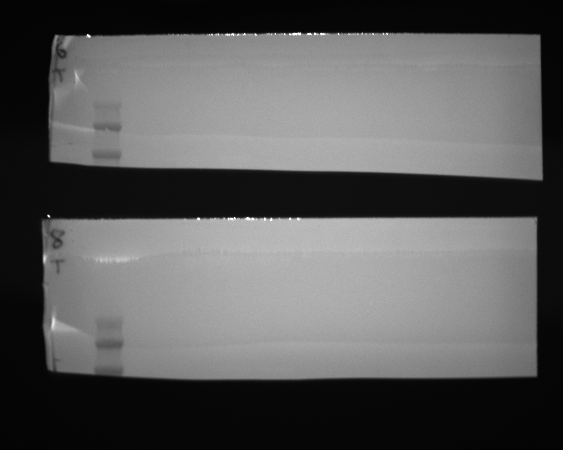

Supplement: Figure 5—source data 1. [file elife-84280-fig5-data1.zip › Figure 5 - Source data 1 - Unedited blots/n1-5 HIF1/CHEMI_07012022_195842_(Membrane)_raw.tif]

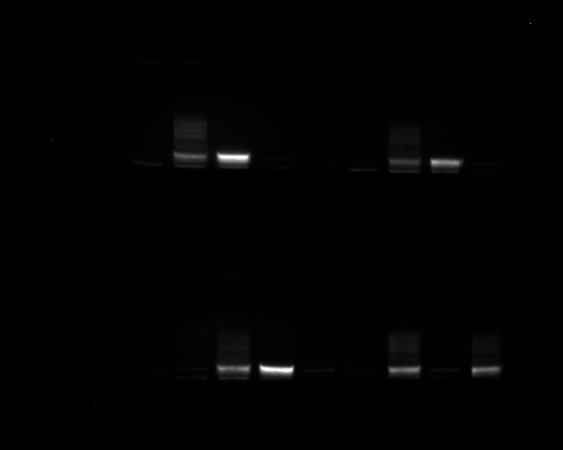

Supplement: Figure 5—source data 1. [file elife-84280-fig5-data1.zip › Figure 5 - Source data 1 - Unedited blots/n1-5 HIF1/CHEMI_07012022_195850_(Chemi)_raw.tif]

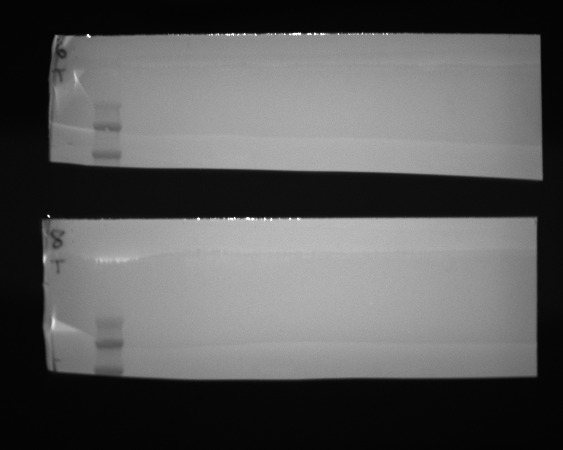

Supplement: Figure 5—source data 1. [file elife-84280-fig5-data1.zip › Figure 5 - Source data 1 - Unedited blots/n1-5 HIF1/CHEMI_07012022_195850_(Membrane)_raw.tif]

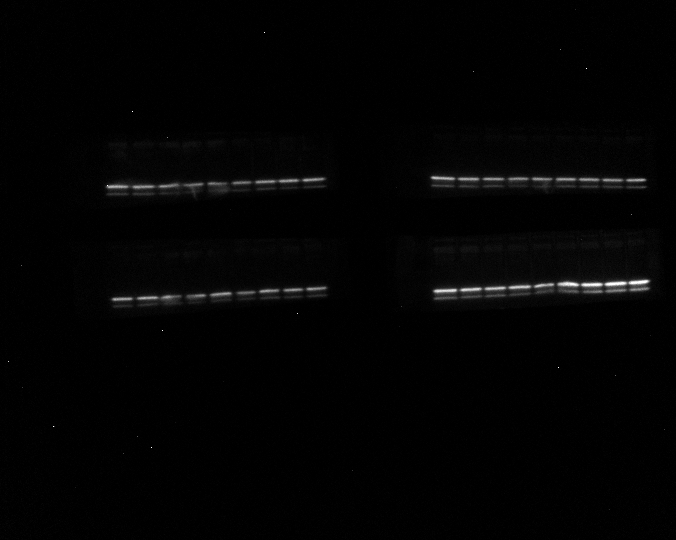

Supplement: Figure 5—source data 1. [file elife-84280-fig5-data1.zip › Figure 5 - Source data 1 - Unedited blots/n1-5 Histone3/CHEMI_07012022_184318/CHEMI_07012022_184318_(Chemi)_raw.tif]

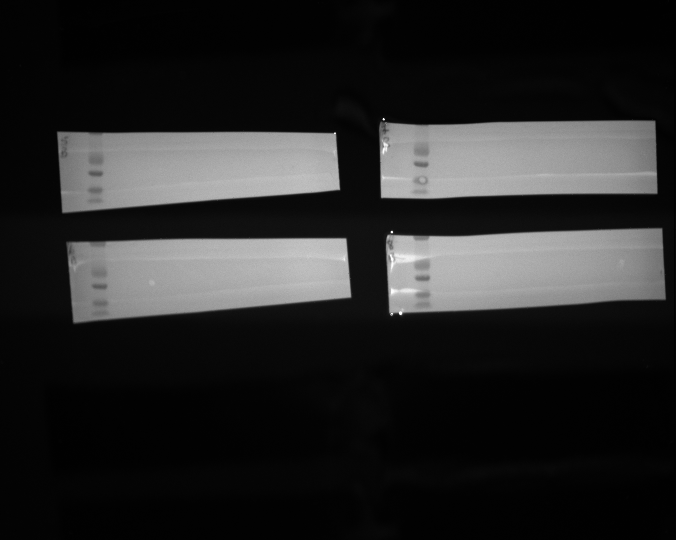

Supplement: Figure 5—source data 1. [file elife-84280-fig5-data1.zip › Figure 5 - Source data 1 - Unedited blots/n1-5 Histone3/CHEMI_07012022_184318/CHEMI_07012022_184318_(Membrane)_raw.tif]

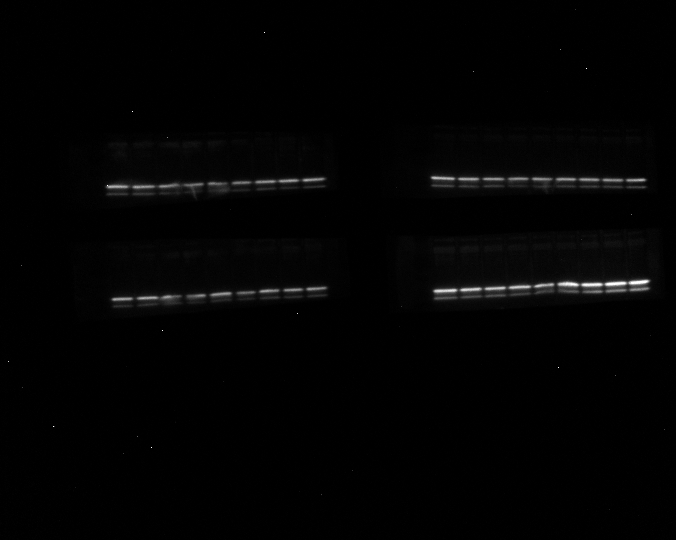

Supplement: Figure 5—source data 1. [file elife-84280-fig5-data1.zip › Figure 5 - Source data 1 - Unedited blots/n1-5 Histone3/CHEMI_07012022_184338/CHEMI_07012022_184338_(Chemi)_raw.tif]

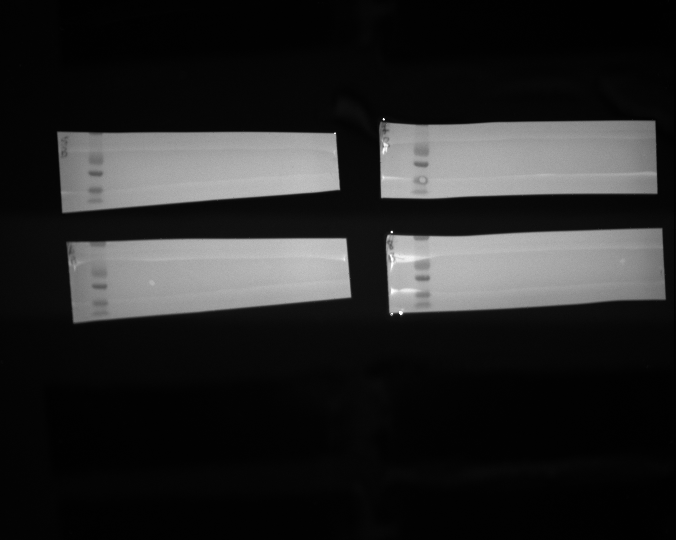

Supplement: Figure 5—source data 1. [file elife-84280-fig5-data1.zip › Figure 5 - Source data 1 - Unedited blots/n1-5 Histone3/CHEMI_07012022_184338/CHEMI_07012022_184338_(Membrane)_raw.tif]

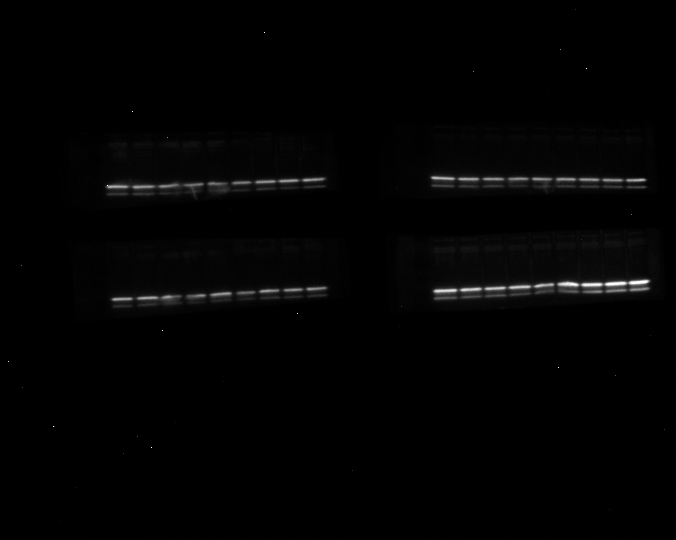

Supplement: Figure 5—source data 1. [file elife-84280-fig5-data1.zip › Figure 5 - Source data 1 - Unedited blots/n1-5 Histone3/CHEMI_07012022_184413/CHEMI_07012022_184413_(Chemi)_raw.tif]

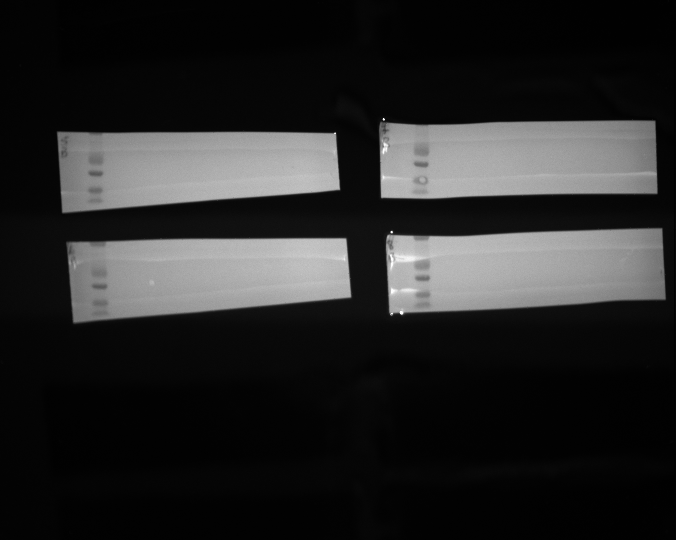

Supplement: Figure 5—source data 1. [file elife-84280-fig5-data1.zip › Figure 5 - Source data 1 - Unedited blots/n1-5 Histone3/CHEMI_07012022_184413/CHEMI_07012022_184413_(Membrane)_raw.tif]

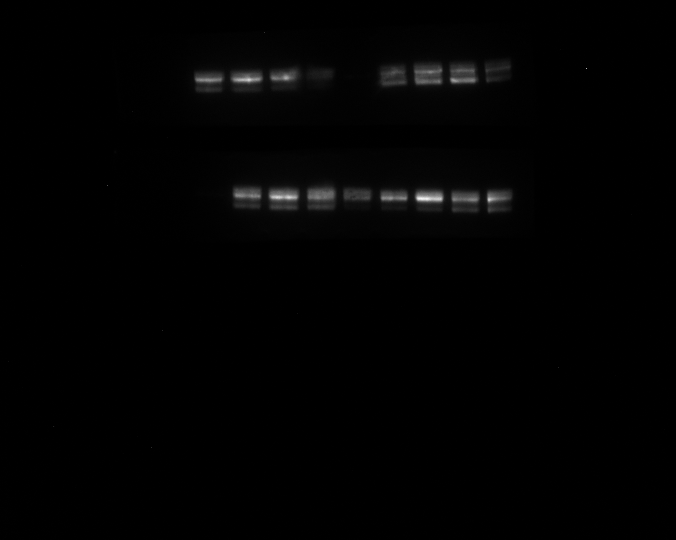

Supplement: Figure 5—source data 1. [file elife-84280-fig5-data1.zip › Figure 5 - Source data 1 - Unedited blots/n1-5 NFIL3/CHEMI_07012022_183530/CHEMI_07012022_183530_(Chemi)_raw.tif]

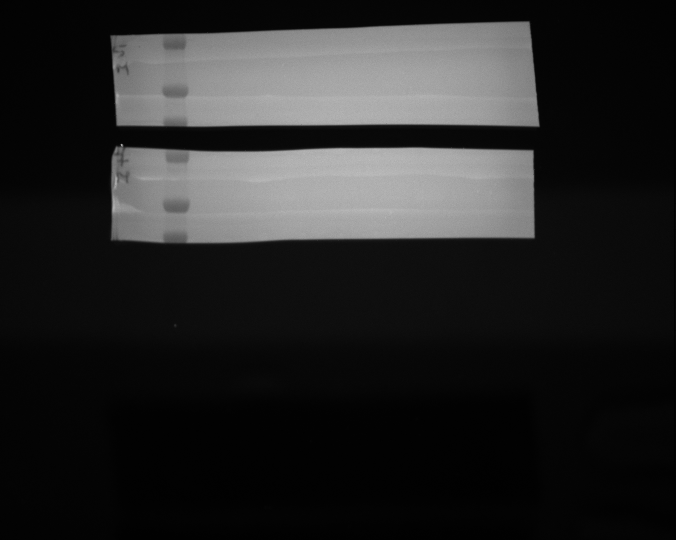

Supplement: Figure 5—source data 1. [file elife-84280-fig5-data1.zip › Figure 5 - Source data 1 - Unedited blots/n1-5 NFIL3/CHEMI_07012022_183530/CHEMI_07012022_183530_(Membrane)_raw.tif]

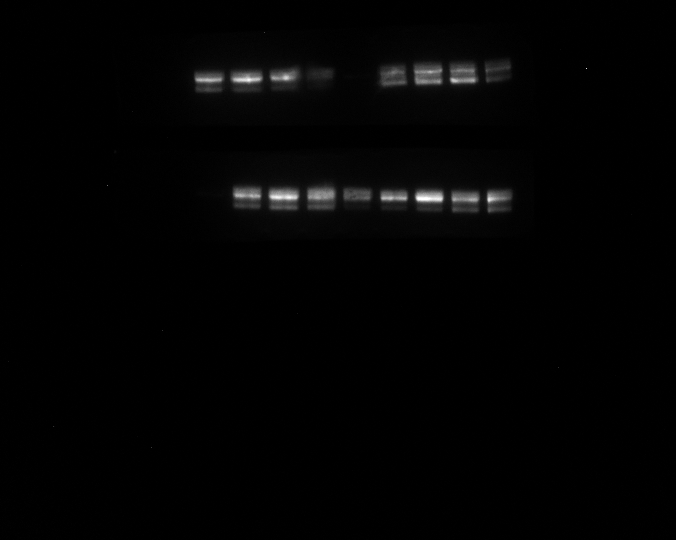

Supplement: Figure 5—source data 1. [file elife-84280-fig5-data1.zip › Figure 5 - Source data 1 - Unedited blots/n1-5 NFIL3/CHEMI_07012022_183546/CHEMI_07012022_183546_(Chemi)_raw.tif]

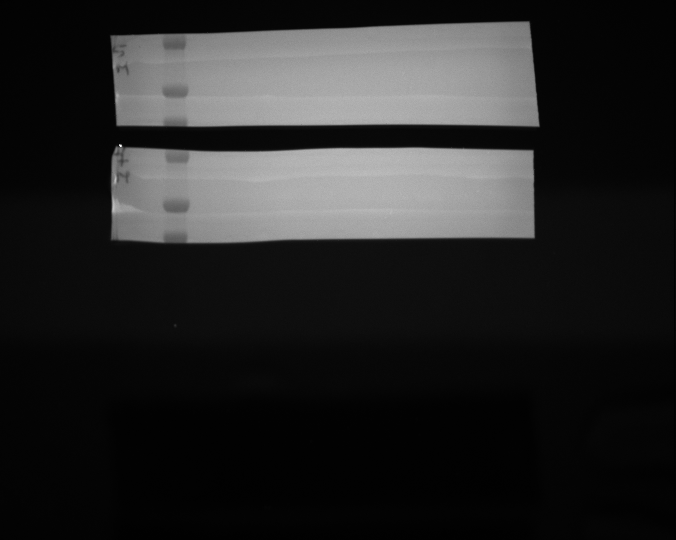

Supplement: Figure 5—source data 1. [file elife-84280-fig5-data1.zip › Figure 5 - Source data 1 - Unedited blots/n1-5 NFIL3/CHEMI_07012022_183546/CHEMI_07012022_183546_(Membrane)_raw.tif]

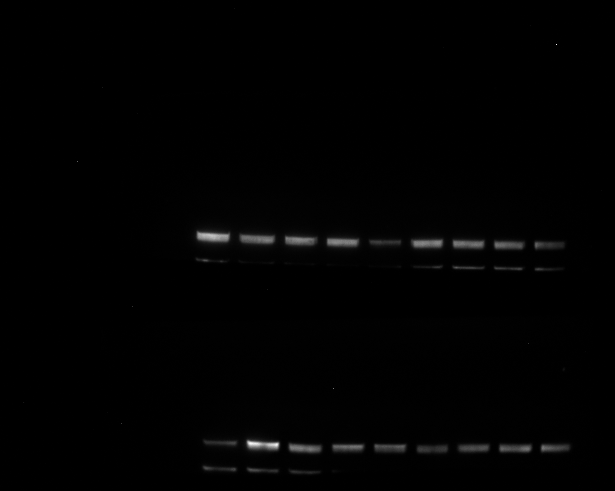

Supplement: Figure 5—source data 1. [file elife-84280-fig5-data1.zip › Figure 5 - Source data 1 - Unedited blots/n1-5 nuclear NFAT/CHEMI_07022022_004239/CHEMI_07022022_004239_(Chemi)_raw.tif]

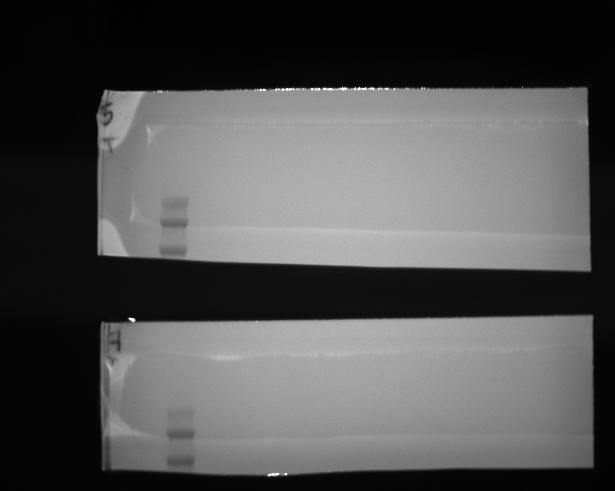

Supplement: Figure 5—source data 1. [file elife-84280-fig5-data1.zip › Figure 5 - Source data 1 - Unedited blots/n1-5 nuclear NFAT/CHEMI_07022022_004239/CHEMI_07022022_004239_(Membrane)_raw.tif]

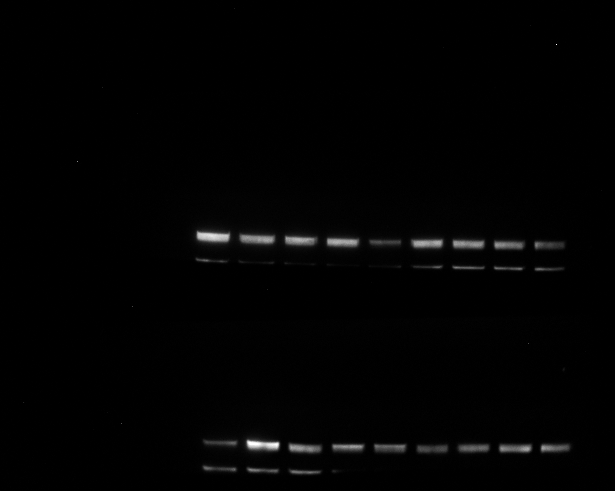

Supplement: Figure 5—source data 1. [file elife-84280-fig5-data1.zip › Figure 5 - Source data 1 - Unedited blots/n1-5 nuclear NFAT/CHEMI_07022022_004300/CHEMI_07022022_004300_(Chemi)_raw.tif]

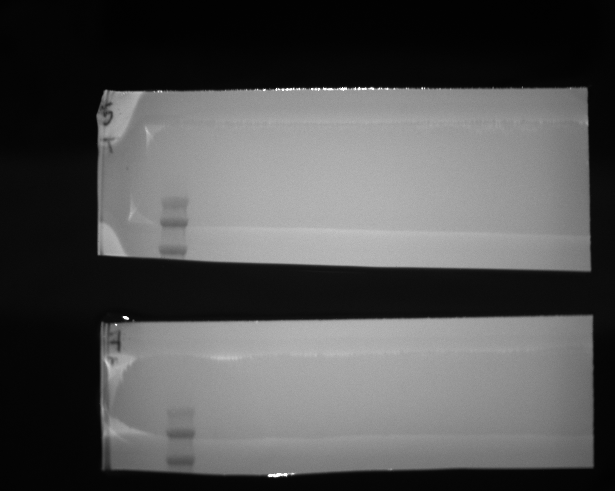

Supplement: Figure 5—source data 1. [file elife-84280-fig5-data1.zip › Figure 5 - Source data 1 - Unedited blots/n1-5 nuclear NFAT/CHEMI_07022022_004300/CHEMI_07022022_004300_(Membrane)_raw.tif]

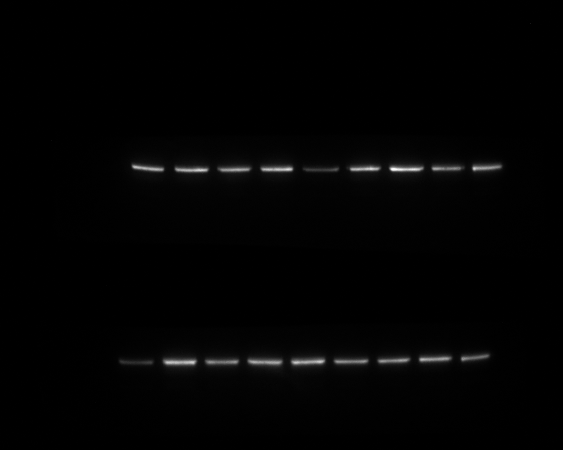

Supplement: Figure 5—source data 1. [file elife-84280-fig5-data1.zip › Figure 5 - Source data 1 - Unedited blots/n1-5 nuclear NFkB/CHEMI_07012022_182407/CHEMI_07012022_182407_(Chemi)_raw.tif]

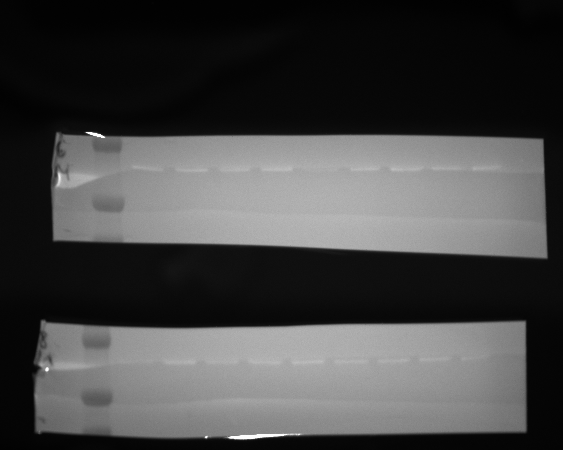

Supplement: Figure 5—source data 1. [file elife-84280-fig5-data1.zip › Figure 5 - Source data 1 - Unedited blots/n1-5 nuclear NFkB/CHEMI_07012022_182407/CHEMI_07012022_182407_(Membrane)_raw.tif]

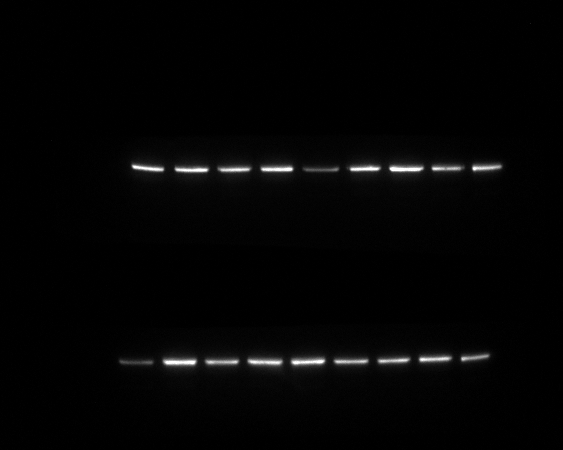

Supplement: Figure 5—source data 1. [file elife-84280-fig5-data1.zip › Figure 5 - Source data 1 - Unedited blots/n1-5 nuclear NFkB/CHEMI_07012022_182418/CHEMI_07012022_182418_(Chemi)_raw.tif]

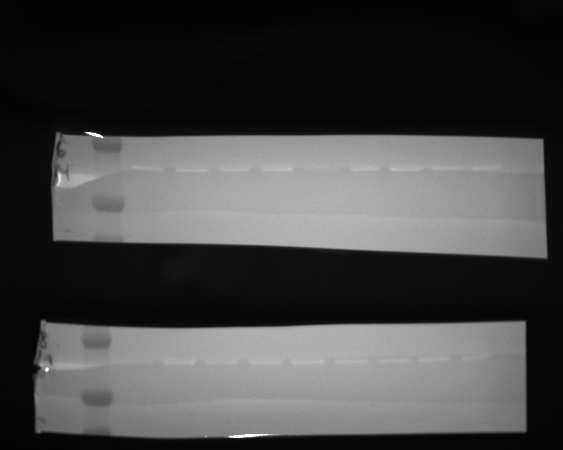

Supplement: Figure 5—source data 1. [file elife-84280-fig5-data1.zip › Figure 5 - Source data 1 - Unedited blots/n1-5 nuclear NFkB/CHEMI_07012022_182418/CHEMI_07012022_182418_(Membrane)_raw.tif]

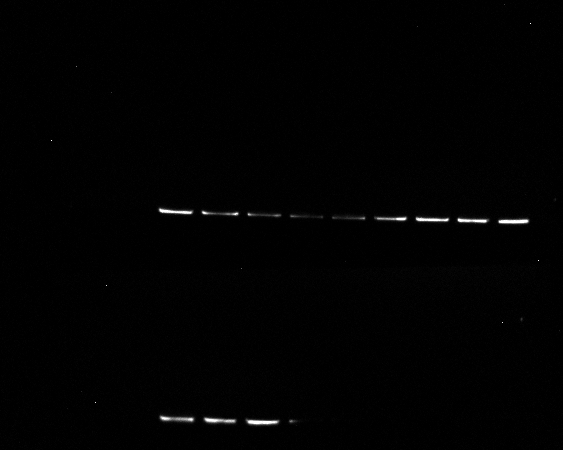

Supplement: Figure 5—source data 1. [file elife-84280-fig5-data1.zip › Figure 5 - Source data 1 - Unedited blots/n1-5 PGC1a/membrane 5 and 3 lanes on membrane 7/CHEMI_07012022_181412_(Chemi)_raw.tif]

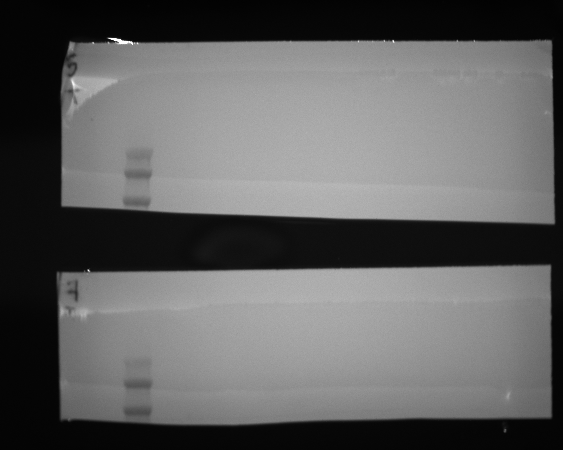

Supplement: Figure 5—source data 1. [file elife-84280-fig5-data1.zip › Figure 5 - Source data 1 - Unedited blots/n1-5 PGC1a/membrane 5 and 3 lanes on membrane 7/CHEMI_07012022_181412_(Membrane)_raw.tif]

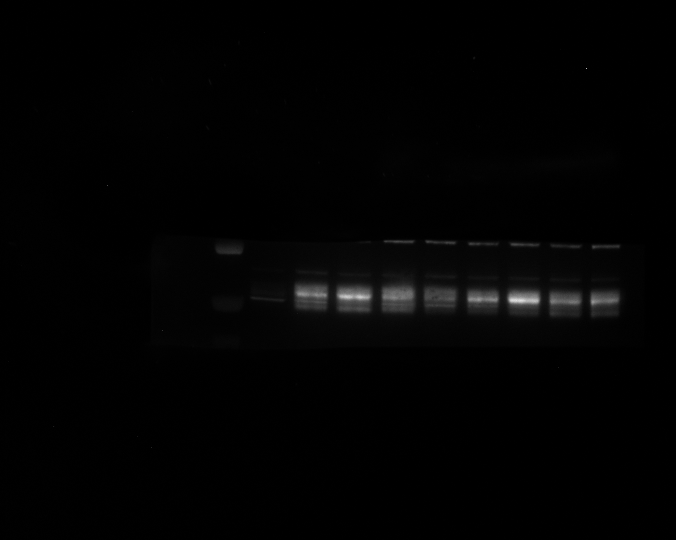

Supplement: Figure 5—source data 1. [file elife-84280-fig5-data1.zip › Figure 5 - Source data 1 - Unedited blots/n1-5 PGC1a/membrane 7 (2nd half)/CHEMI_07022022_023105_(Chemi)_raw.tif]

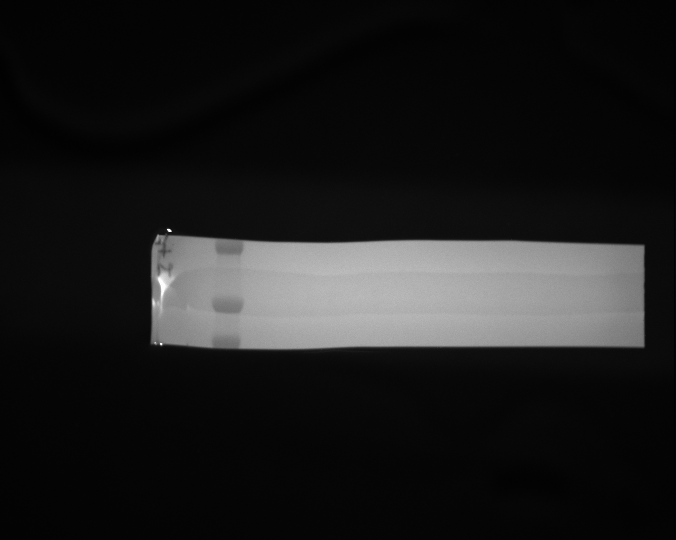

Supplement: Figure 5—source data 1. [file elife-84280-fig5-data1.zip › Figure 5 - Source data 1 - Unedited blots/n1-5 PGC1a/membrane 7 (2nd half)/CHEMI_07022022_023105_(Membrane)_raw.tif]

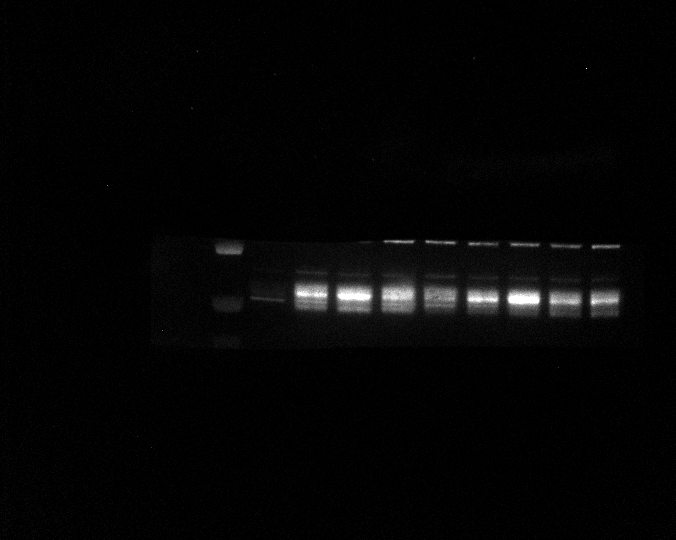

Supplement: Figure 5—source data 1. [file elife-84280-fig5-data1.zip › Figure 5 - Source data 1 - Unedited blots/n1-5 PGC1a/membrane 7 (2nd half)/CHEMI_07022022_023146_(Chemi)_raw.tif]

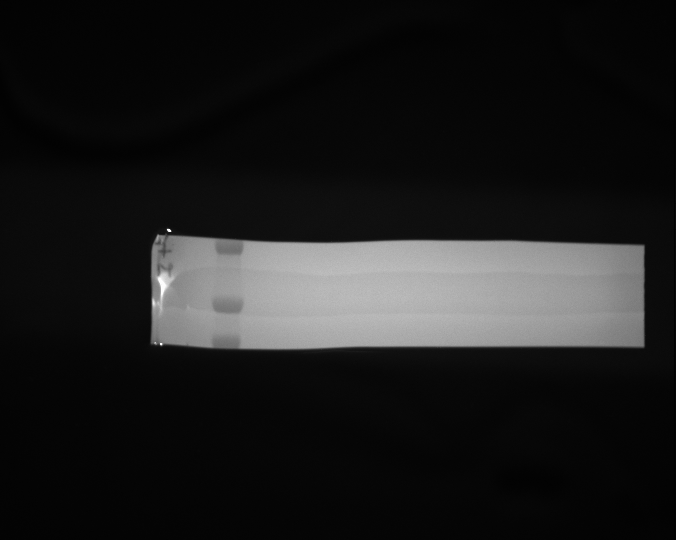

Supplement: Figure 5—source data 1. [file elife-84280-fig5-data1.zip › Figure 5 - Source data 1 - Unedited blots/n1-5 PGC1a/membrane 7 (2nd half)/CHEMI_07022022_023146_(Membrane)_raw.tif]

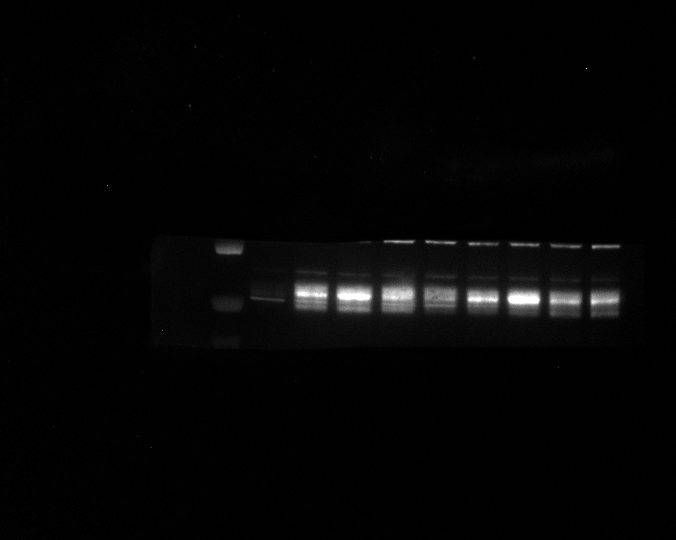

Supplement: Figure 5—source data 1. [file elife-84280-fig5-data1.zip › Figure 5 - Source data 1 - Unedited blots/n1-5 PGC1a/membrane 7 (2nd half)/CHEMI_07022022_023151_(Chemi)_raw.tif]

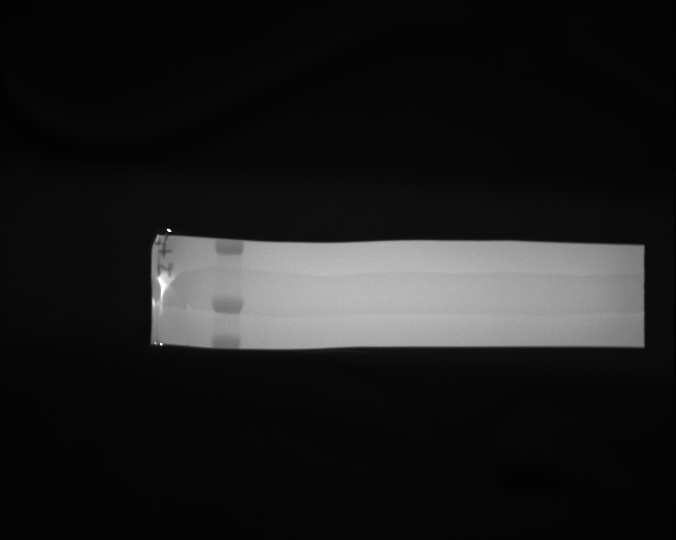

Supplement: Figure 5—source data 1. [file elife-84280-fig5-data1.zip › Figure 5 - Source data 1 - Unedited blots/n1-5 PGC1a/membrane 7 (2nd half)/CHEMI_07022022_023151_(Membrane)_raw.tif]

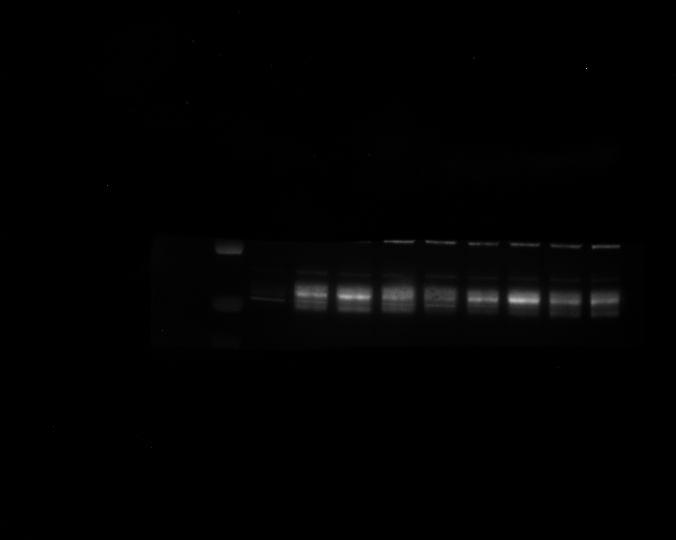

Supplement: Figure 5—source data 1. [file elife-84280-fig5-data1.zip › Figure 5 - Source data 1 - Unedited blots/n1-5 PGC1a/membrane 7 (2nd half)/CHEMI_07022022_023159_(Chemi)_raw.tif]

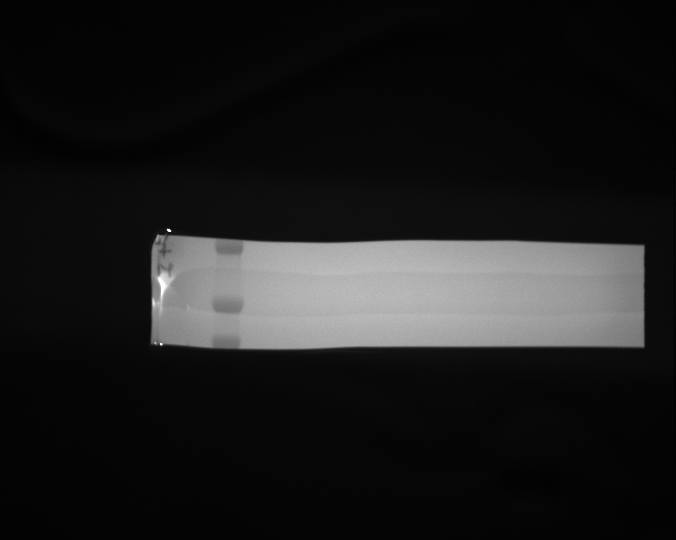

Supplement: Figure 5—source data 1. [file elife-84280-fig5-data1.zip › Figure 5 - Source data 1 - Unedited blots/n1-5 PGC1a/membrane 7 (2nd half)/CHEMI_07022022_023159_(Membrane)_raw.tif]

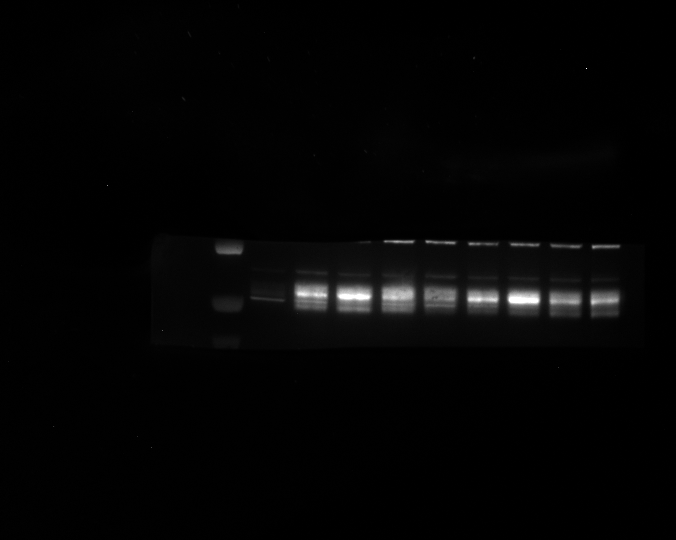

Supplement: Figure 5—source data 1. [file elife-84280-fig5-data1.zip › Figure 5 - Source data 1 - Unedited blots/n1-5 PGC1a/membrane 7 (2nd half)/CHEMI_07022022_023210_(Chemi)_raw.tif]

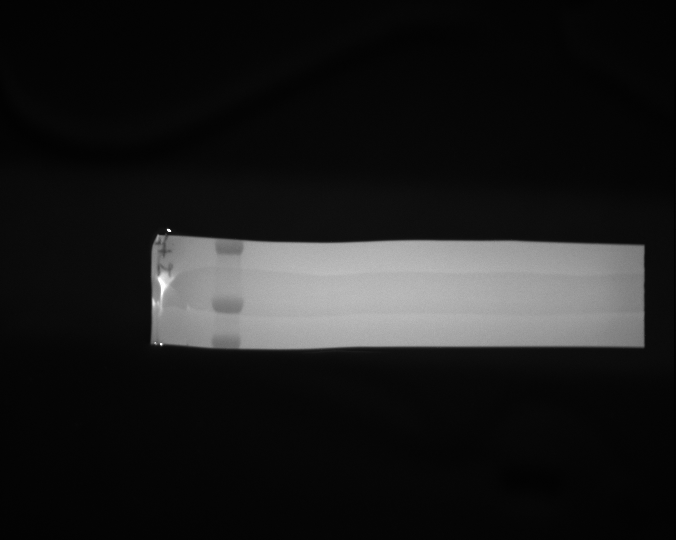

Supplement: Figure 5—source data 1. [file elife-84280-fig5-data1.zip › Figure 5 - Source data 1 - Unedited blots/n1-5 PGC1a/membrane 7 (2nd half)/CHEMI_07022022_023210_(Membrane)_raw.tif]

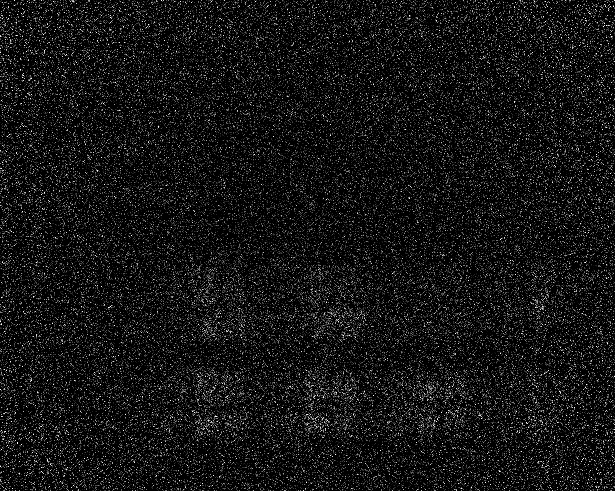

Supplement: Figure 5—source data 1. [file elife-84280-fig5-data1.zip › Figure 5 - Source data 1 - Unedited blots/n1-8 GLUT1/CHEMI_07022022_024309_(Chemi)_raw.tif]

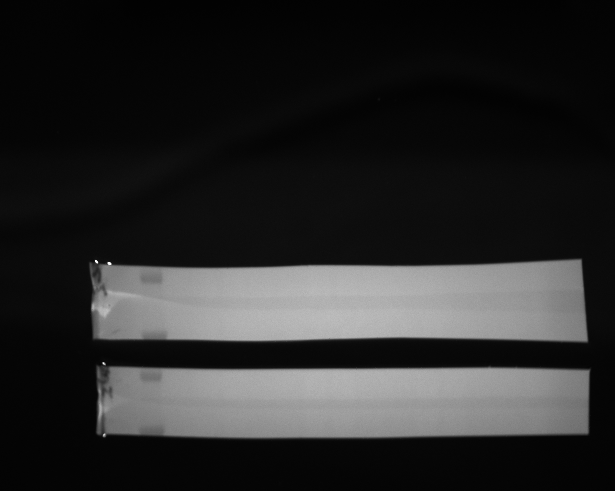

Supplement: Figure 5—source data 1. [file elife-84280-fig5-data1.zip › Figure 5 - Source data 1 - Unedited blots/n1-8 GLUT1/CHEMI_07022022_024309_(Membrane)_raw.tif]

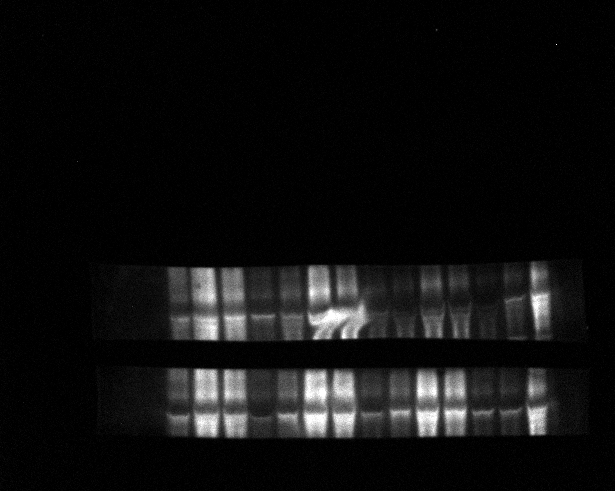

Supplement: Figure 5—source data 1. [file elife-84280-fig5-data1.zip › Figure 5 - Source data 1 - Unedited blots/n1-8 GLUT1/CHEMI_07022022_024314_(Chemi)_raw.tif]

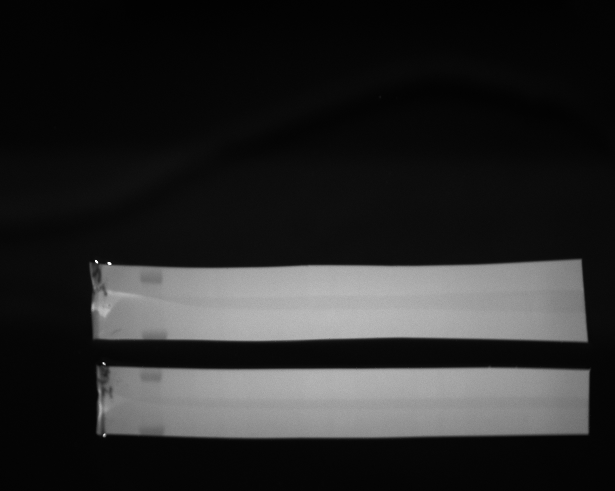

Supplement: Figure 5—source data 1. [file elife-84280-fig5-data1.zip › Figure 5 - Source data 1 - Unedited blots/n1-8 GLUT1/CHEMI_07022022_024314_(Membrane)_raw.tif]

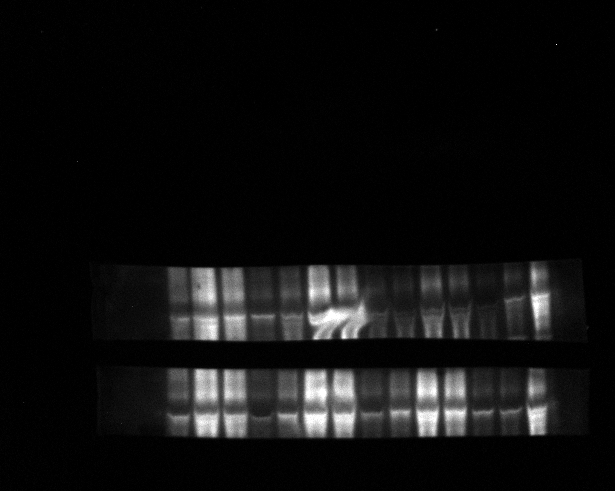

Supplement: Figure 5—source data 1. [file elife-84280-fig5-data1.zip › Figure 5 - Source data 1 - Unedited blots/n1-8 GLUT1/CHEMI_07022022_024319_(Chemi)_raw.tif]

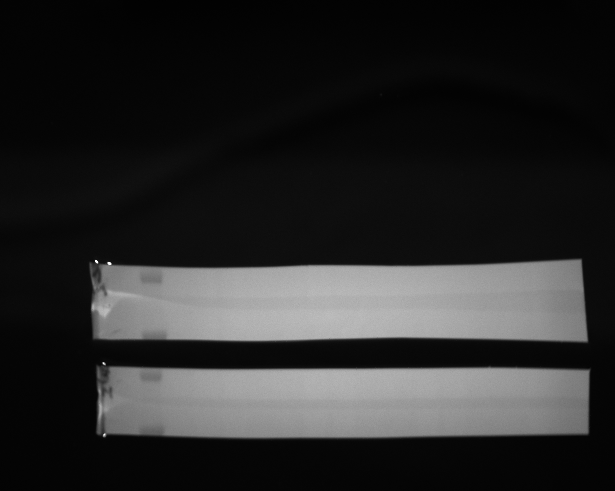

Supplement: Figure 5—source data 1. [file elife-84280-fig5-data1.zip › Figure 5 - Source data 1 - Unedited blots/n1-8 GLUT1/CHEMI_07022022_024319_(Membrane)_raw.tif]

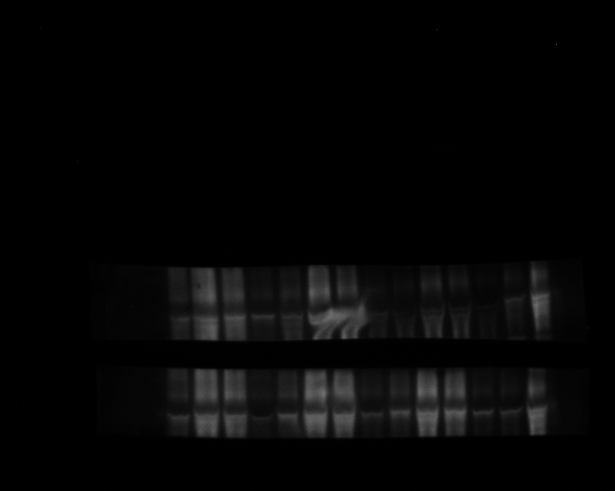

Supplement: Figure 5—source data 1. [file elife-84280-fig5-data1.zip › Figure 5 - Source data 1 - Unedited blots/n1-8 GLUT1/CHEMI_07022022_024325_(Chemi)_raw.tif]

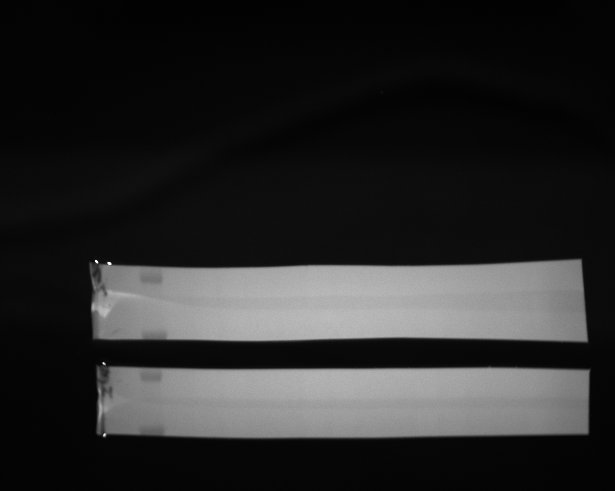

Supplement: Figure 5—source data 1. [file elife-84280-fig5-data1.zip › Figure 5 - Source data 1 - Unedited blots/n1-8 GLUT1/CHEMI_07022022_024325_(Membrane)_raw.tif]

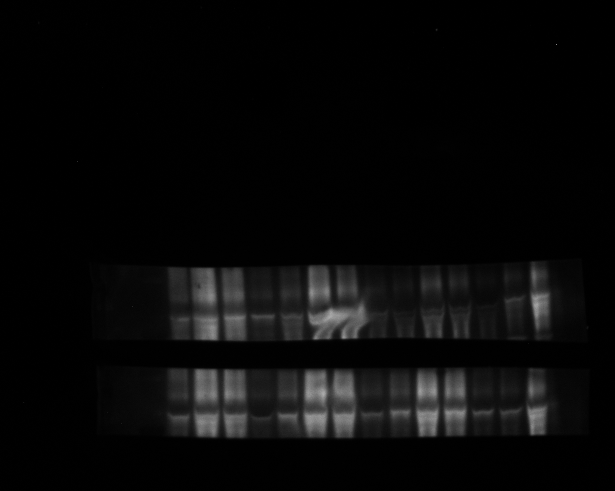

Supplement: Figure 5—source data 1. [file elife-84280-fig5-data1.zip › Figure 5 - Source data 1 - Unedited blots/n1-8 GLUT1/CHEMI_07022022_024333_(Chemi)_raw.tif]

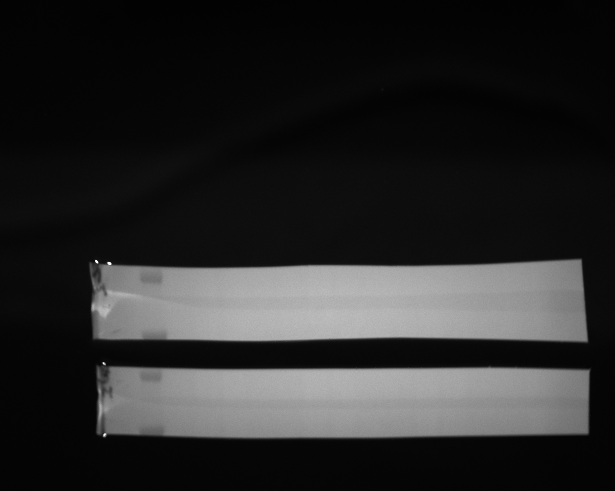

Supplement: Figure 5—source data 1. [file elife-84280-fig5-data1.zip › Figure 5 - Source data 1 - Unedited blots/n1-8 GLUT1/CHEMI_07022022_024333_(Membrane)_raw.tif]

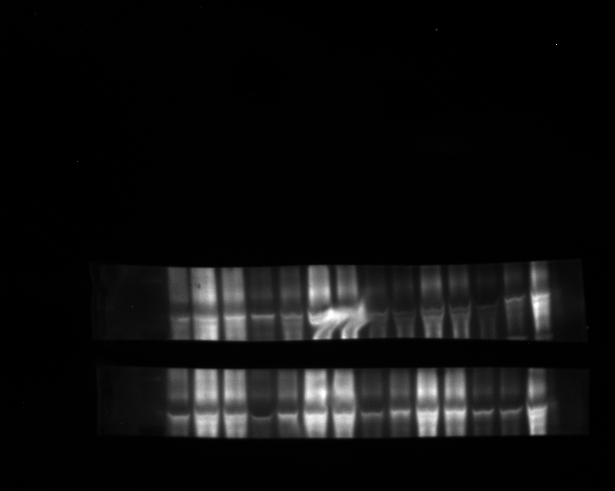

Supplement: Figure 5—source data 1. [file elife-84280-fig5-data1.zip › Figure 5 - Source data 1 - Unedited blots/n1-8 GLUT1/CHEMI_07022022_024348_(Chemi)_raw.tif]

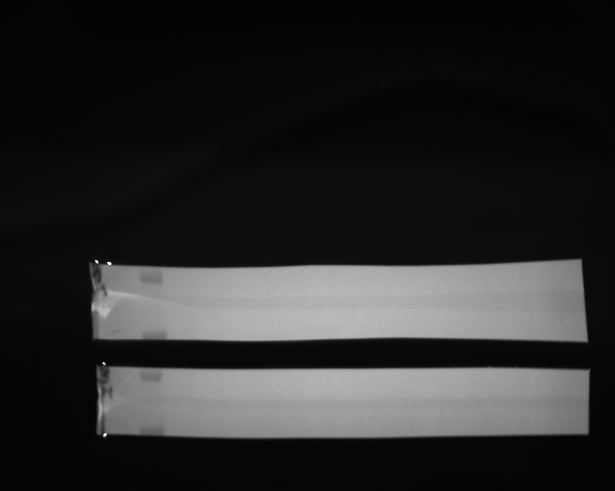

Supplement: Figure 5—source data 1. [file elife-84280-fig5-data1.zip › Figure 5 - Source data 1 - Unedited blots/n1-8 GLUT1/CHEMI_07022022_024348_(Membrane)_raw.tif]

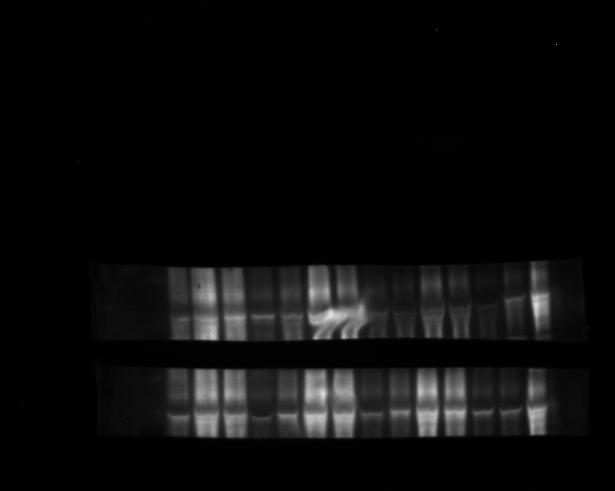

Supplement: Figure 5—source data 1. [file elife-84280-fig5-data1.zip › Figure 5 - Source data 1 - Unedited blots/n1-8 GLUT1/CHEMI_07022022_024425_(Chemi)_raw.tif]

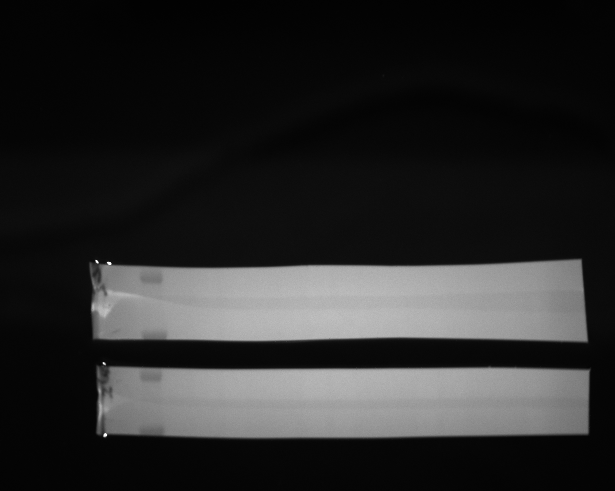

Supplement: Figure 5—source data 1. [file elife-84280-fig5-data1.zip › Figure 5 - Source data 1 - Unedited blots/n1-8 GLUT1/CHEMI_07022022_024425_(Membrane)_raw.tif]
