# Supplementary figures and images for: Oxygen levels at the time of activation determine T cell persistence and immunotherapeutic efficacy (part 2 of 2)
Source: eLife. 2023 May 11;12:e84280. doi: 10.7554/eLife.84280 (PMC10229120; doi:10.7554/eLife.84280)

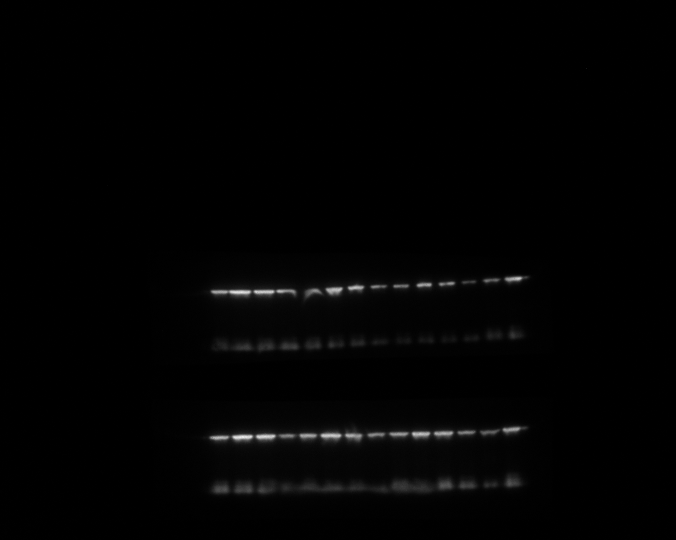

Supplement: Figure 5—source data 1. [file elife-84280-fig5-data1.zip › Figure 5 - Source data 1 - Unedited blots/n1-8 LDHA/CHEMI_07022022_025641_(Chemi)_raw.tif]

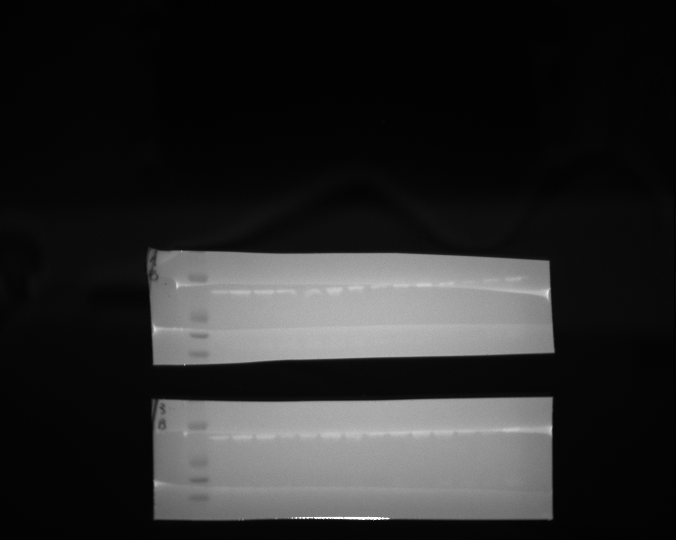

Supplement: Figure 5—source data 1. [file elife-84280-fig5-data1.zip › Figure 5 - Source data 1 - Unedited blots/n1-8 LDHA/CHEMI_07022022_025641_(Membrane)_raw.tif]

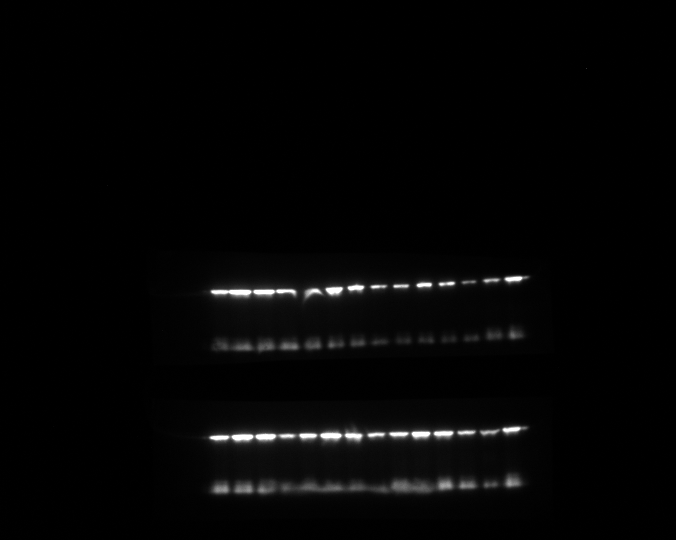

Supplement: Figure 5—source data 1. [file elife-84280-fig5-data1.zip › Figure 5 - Source data 1 - Unedited blots/n1-8 LDHA/CHEMI_07022022_025646_(Chemi)_raw.tif]

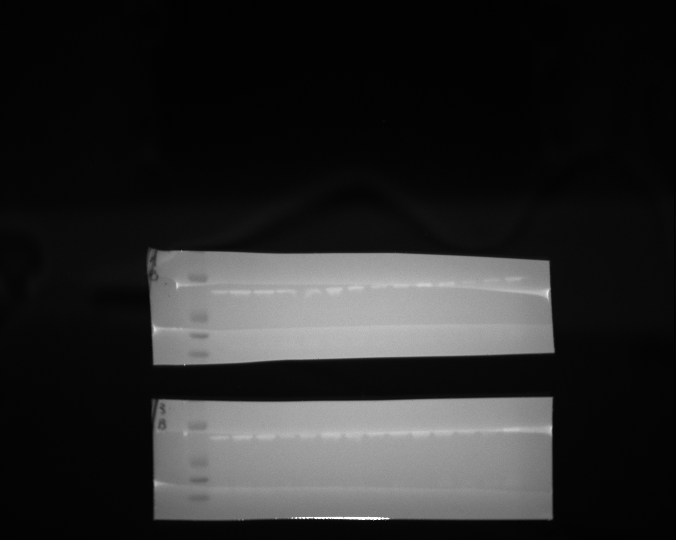

Supplement: Figure 5—source data 1. [file elife-84280-fig5-data1.zip › Figure 5 - Source data 1 - Unedited blots/n1-8 LDHA/CHEMI_07022022_025646_(Membrane)_raw.tif]

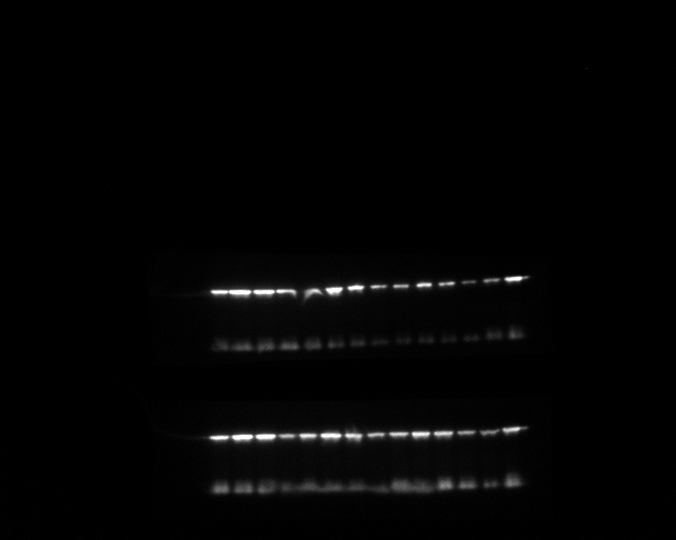

Supplement: Figure 5—source data 1. [file elife-84280-fig5-data1.zip › Figure 5 - Source data 1 - Unedited blots/n1-8 LDHA/CHEMI_07022022_025724_(Chemi)_raw.tif]

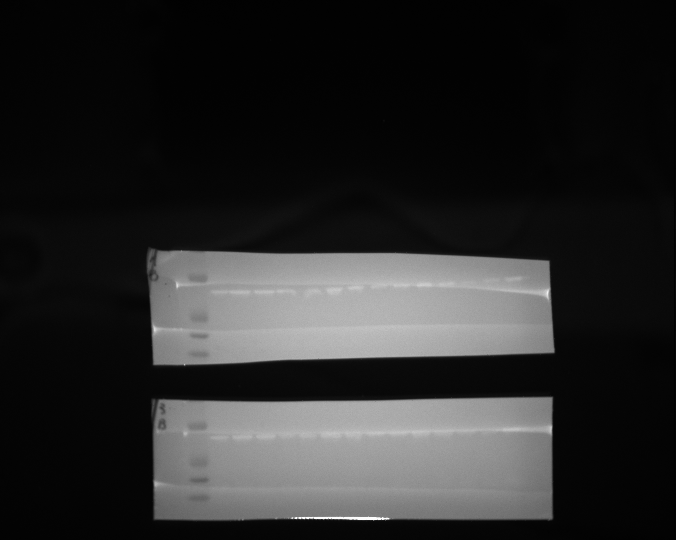

Supplement: Figure 5—source data 1. [file elife-84280-fig5-data1.zip › Figure 5 - Source data 1 - Unedited blots/n1-8 LDHA/CHEMI_07022022_025724_(Membrane)_raw.tif]

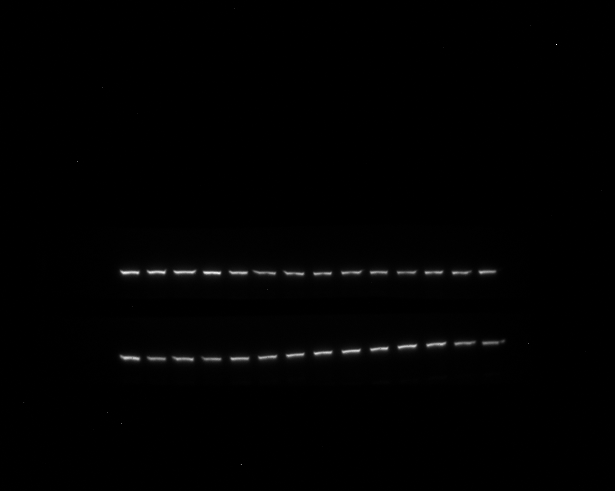

Supplement: Figure 5—source data 1. [file elife-84280-fig5-data1.zip › Figure 5 - Source data 1 - Unedited blots/n5-8 Vinculin (bottom)/CHEMI_07012022_204740_(Chemi)_raw.tif]

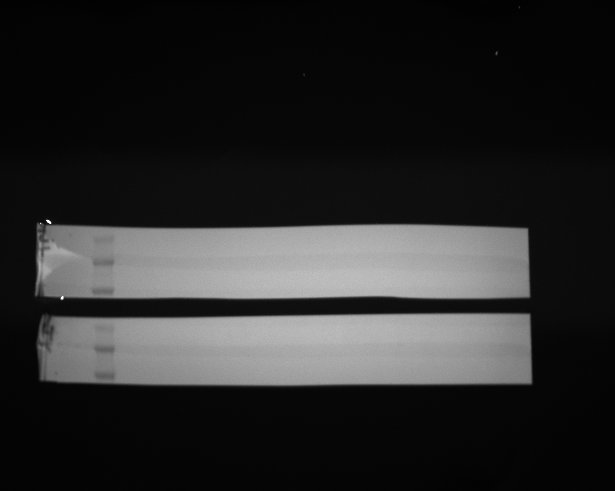

Supplement: Figure 5—source data 1. [file elife-84280-fig5-data1.zip › Figure 5 - Source data 1 - Unedited blots/n5-8 Vinculin (bottom)/CHEMI_07012022_204740_(Membrane)_raw.tif]

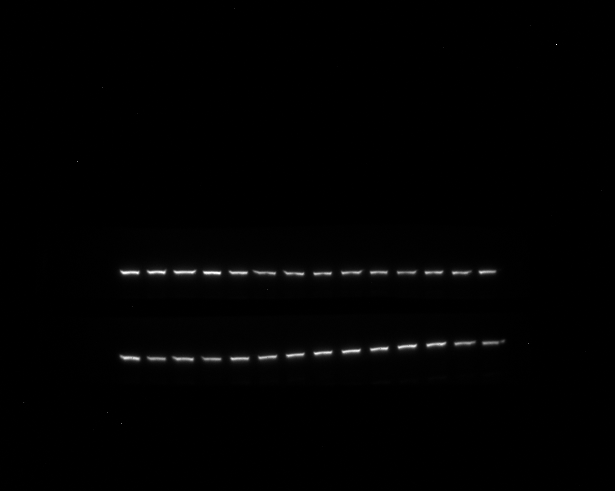

Supplement: Figure 5—source data 1. [file elife-84280-fig5-data1.zip › Figure 5 - Source data 1 - Unedited blots/n5-8 Vinculin (bottom)/CHEMI_07012022_204808_(Chemi)_raw.tif]

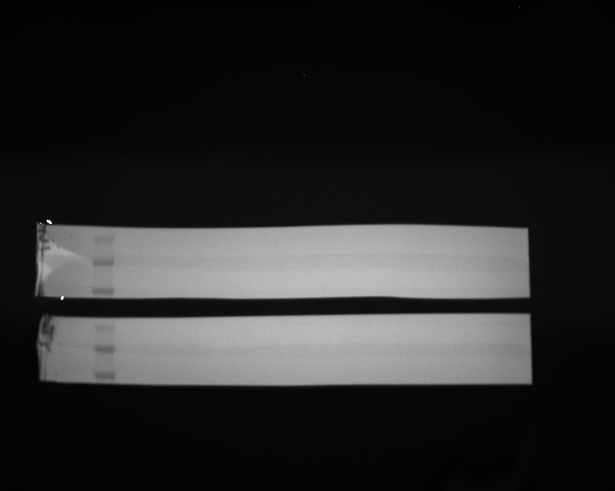

Supplement: Figure 5—source data 1. [file elife-84280-fig5-data1.zip › Figure 5 - Source data 1 - Unedited blots/n5-8 Vinculin (bottom)/CHEMI_07012022_204808_(Membrane)_raw.tif]

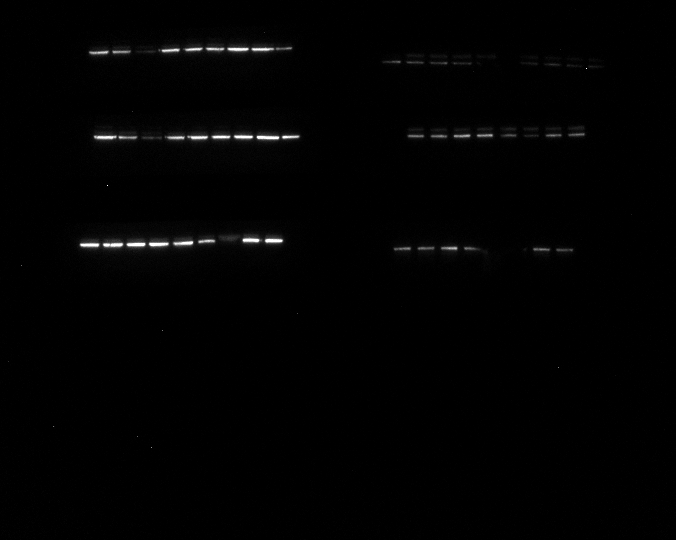

Supplement: Figure 5—source data 1. [file elife-84280-fig5-data1.zip › Figure 5 - Source data 1 - Unedited blots/n6 HDAC (top right)/CHEMI_07012022_002242/CHEMI_07012022_002242_(Chemi)_raw.tif]

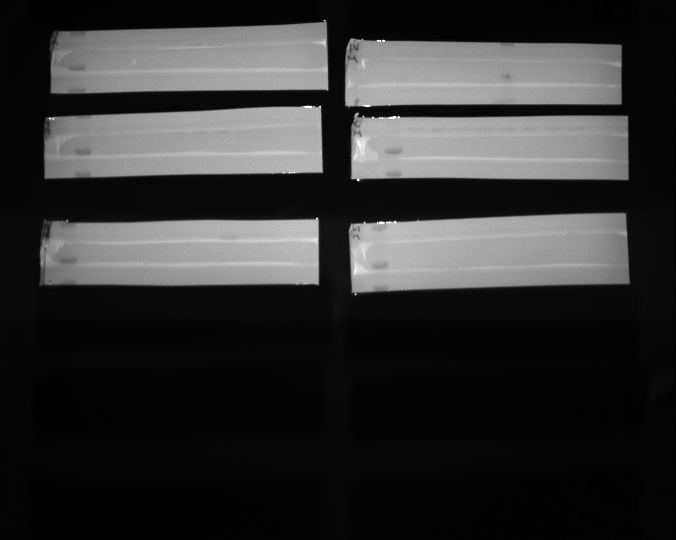

Supplement: Figure 5—source data 1. [file elife-84280-fig5-data1.zip › Figure 5 - Source data 1 - Unedited blots/n6 HDAC (top right)/CHEMI_07012022_002242/CHEMI_07012022_002242_(Membrane)_raw.tif]

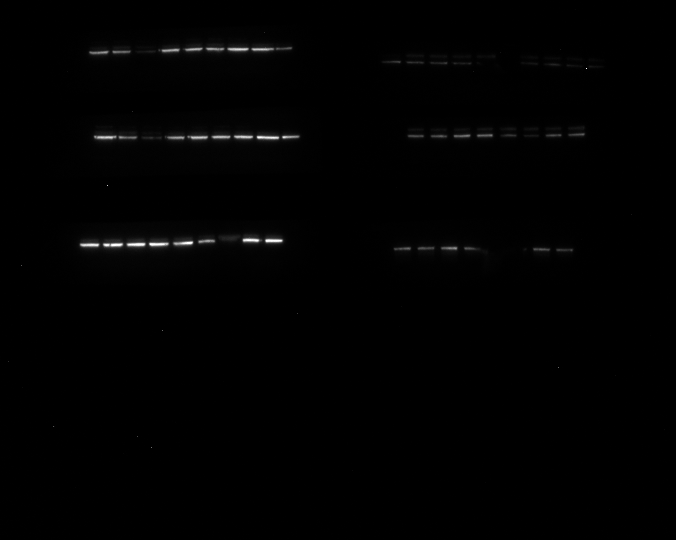

Supplement: Figure 5—source data 1. [file elife-84280-fig5-data1.zip › Figure 5 - Source data 1 - Unedited blots/n6 HDAC (top right)/CHEMI_07012022_002308/CHEMI_07012022_002308_(Chemi)_raw.tif]

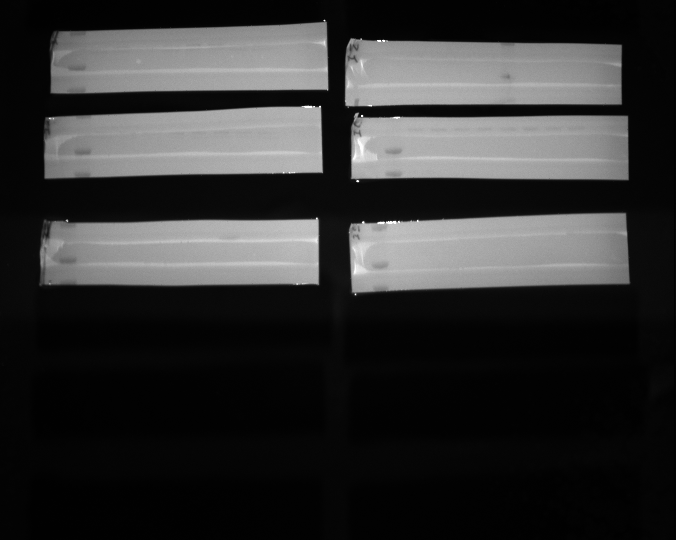

Supplement: Figure 5—source data 1. [file elife-84280-fig5-data1.zip › Figure 5 - Source data 1 - Unedited blots/n6 HDAC (top right)/CHEMI_07012022_002308/CHEMI_07012022_002308_(Membrane)_raw.tif]

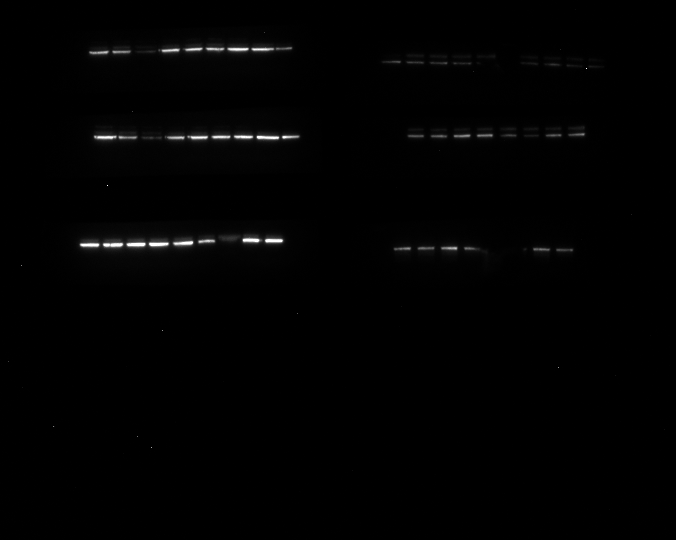

Supplement: Figure 5—source data 1. [file elife-84280-fig5-data1.zip › Figure 5 - Source data 1 - Unedited blots/n6 HDAC (top right)/CHEMI_07012022_002333/CHEMI_07012022_002333_(Chemi)_raw.tif]

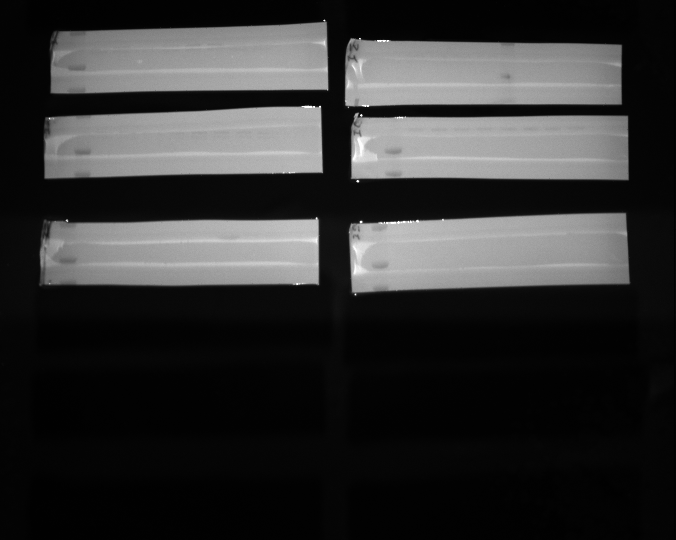

Supplement: Figure 5—source data 1. [file elife-84280-fig5-data1.zip › Figure 5 - Source data 1 - Unedited blots/n6 HDAC (top right)/CHEMI_07012022_002333/CHEMI_07012022_002333_(Membrane)_raw.tif]

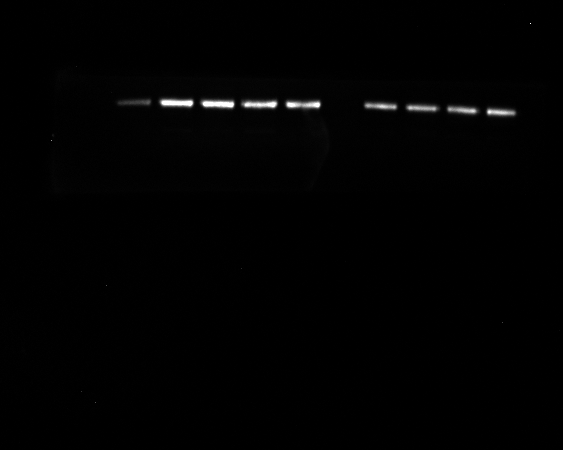

Supplement: Figure 5—source data 1. [file elife-84280-fig5-data1.zip › Figure 5 - Source data 1 - Unedited blots/n6 nuclear NFAT/CHEMI_06292022_203511_(Chemi)_raw.tif]

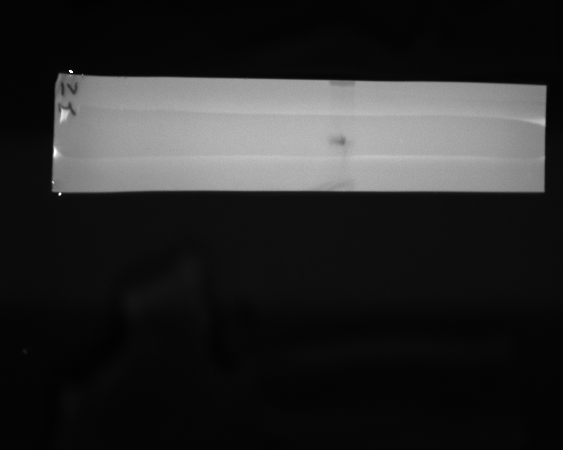

Supplement: Figure 5—source data 1. [file elife-84280-fig5-data1.zip › Figure 5 - Source data 1 - Unedited blots/n6 nuclear NFAT/CHEMI_06292022_203511_(Membrane)_raw.tif]

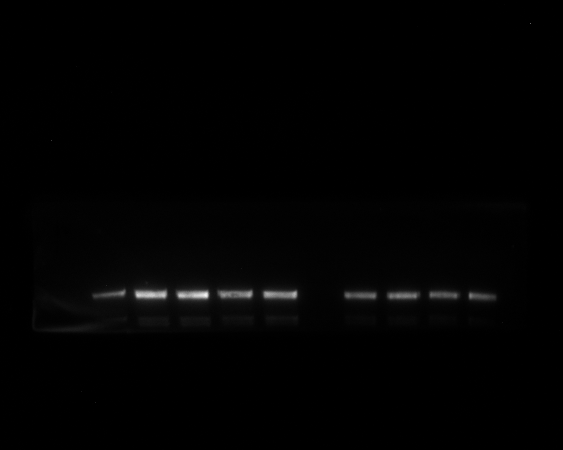

Supplement: Figure 5—source data 1. [file elife-84280-fig5-data1.zip › Figure 5 - Source data 1 - Unedited blots/n6 nuclear NFAT/CHEMI_06292022_205635_(Chemi)_raw.tif]

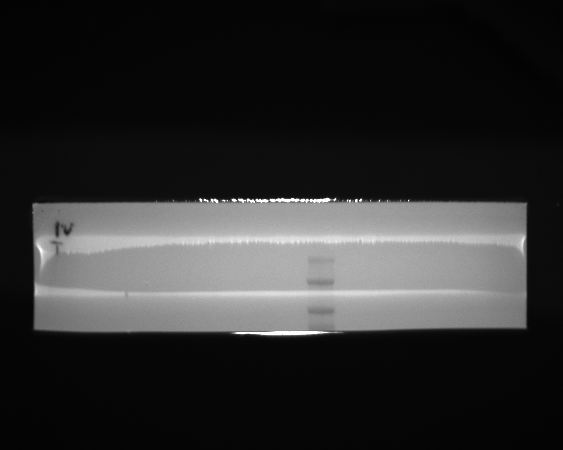

Supplement: Figure 5—source data 1. [file elife-84280-fig5-data1.zip › Figure 5 - Source data 1 - Unedited blots/n6 nuclear NFAT/CHEMI_06292022_205635_(Membrane)_raw.tif]

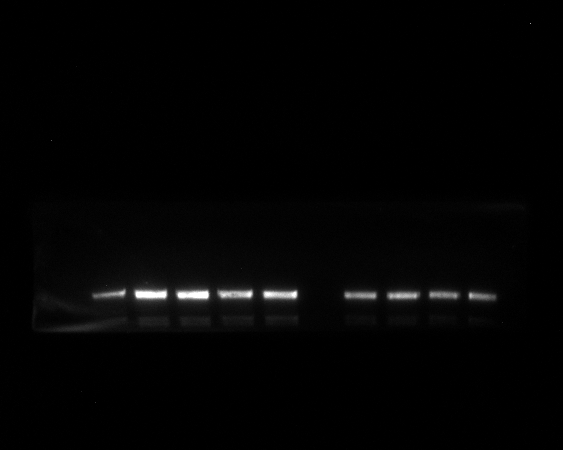

Supplement: Figure 5—source data 1. [file elife-84280-fig5-data1.zip › Figure 5 - Source data 1 - Unedited blots/n6 nuclear NFAT/CHEMI_06292022_205654_(Chemi)_raw.tif]

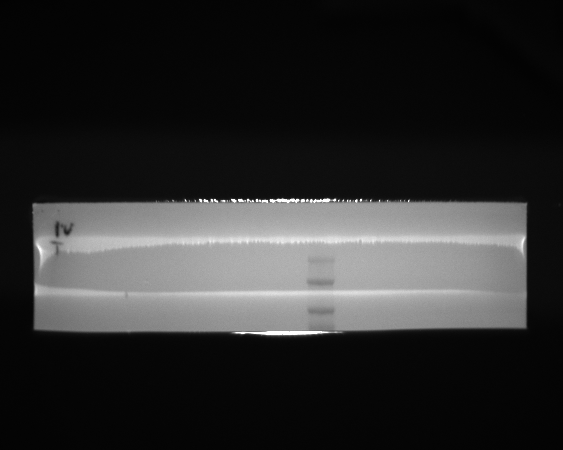

Supplement: Figure 5—source data 1. [file elife-84280-fig5-data1.zip › Figure 5 - Source data 1 - Unedited blots/n6 nuclear NFAT/CHEMI_06292022_205654_(Membrane)_raw.tif]

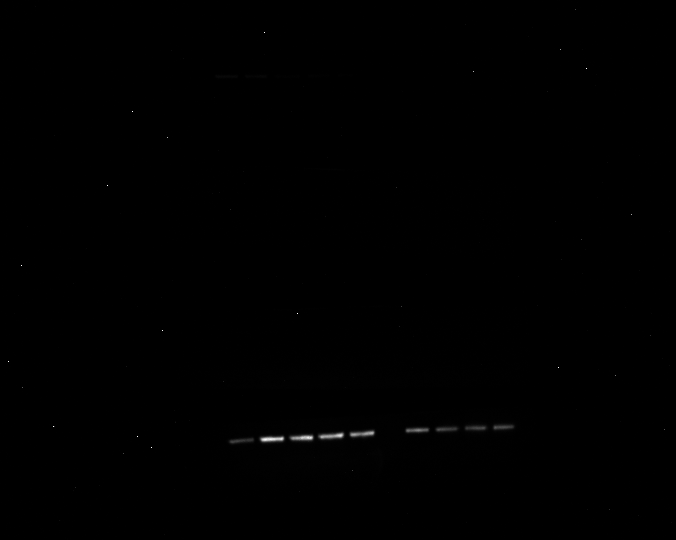

Supplement: Figure 5—source data 1. [file elife-84280-fig5-data1.zip › Figure 5 - Source data 1 - Unedited blots/n6 nuclear NFkB/CHEMI_06292022_202408_(Chemi)_raw.tif]

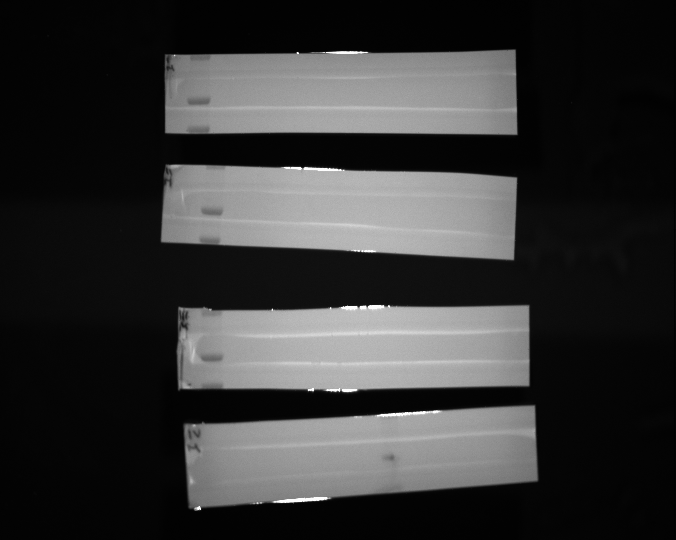

Supplement: Figure 5—source data 1. [file elife-84280-fig5-data1.zip › Figure 5 - Source data 1 - Unedited blots/n6 nuclear NFkB/CHEMI_06292022_202408_(Membrane)_raw.tif]

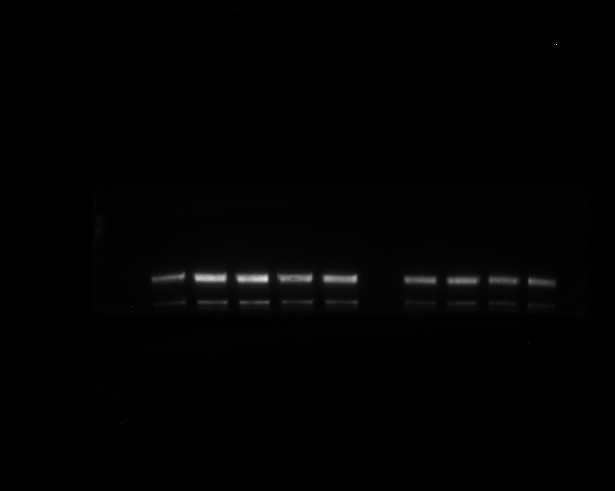

Supplement: Figure 5—source data 1. [file elife-84280-fig5-data1.zip › Figure 5 - Source data 1 - Unedited blots/n6 PGC1a/CHEMI_06302022_184746/CHEMI_06302022_184746_(Chemi)_raw.tif]

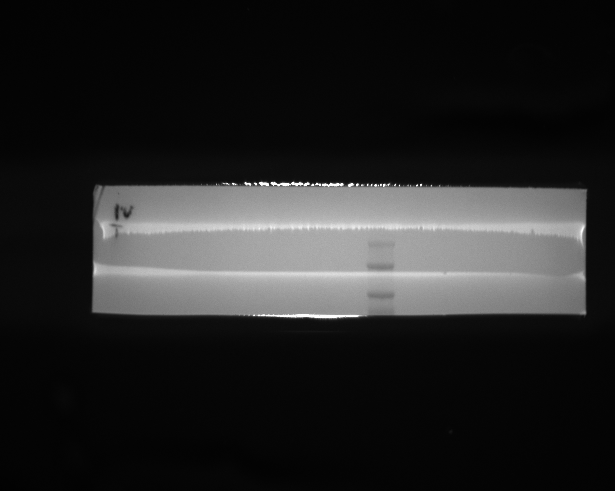

Supplement: Figure 5—source data 1. [file elife-84280-fig5-data1.zip › Figure 5 - Source data 1 - Unedited blots/n6 PGC1a/CHEMI_06302022_184746/CHEMI_06302022_184746_(Membrane)_raw.tif]

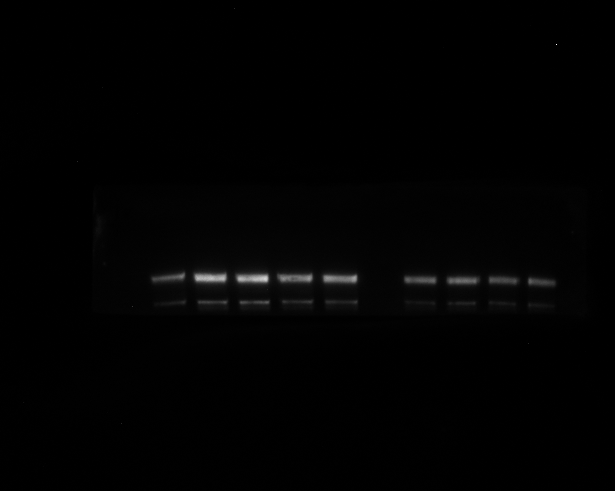

Supplement: Figure 5—source data 1. [file elife-84280-fig5-data1.zip › Figure 5 - Source data 1 - Unedited blots/n6 PGC1a/CHEMI_06302022_184815/CHEMI_06302022_184815_(Chemi)_raw.tif]

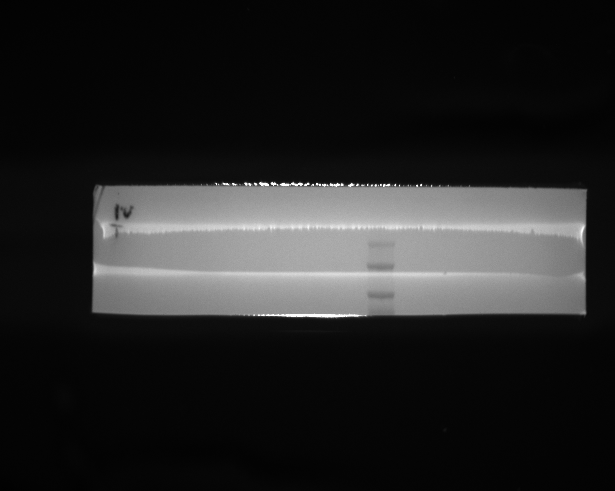

Supplement: Figure 5—source data 1. [file elife-84280-fig5-data1.zip › Figure 5 - Source data 1 - Unedited blots/n6 PGC1a/CHEMI_06302022_184815/CHEMI_06302022_184815_(Membrane)_raw.tif]

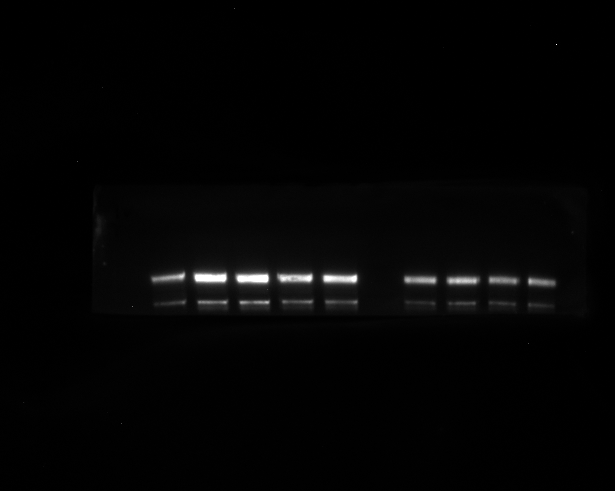

Supplement: Figure 5—source data 1. [file elife-84280-fig5-data1.zip › Figure 5 - Source data 1 - Unedited blots/n6 PGC1a/CHEMI_06302022_184825/CHEMI_06302022_184825_(Chemi)_raw.tif]

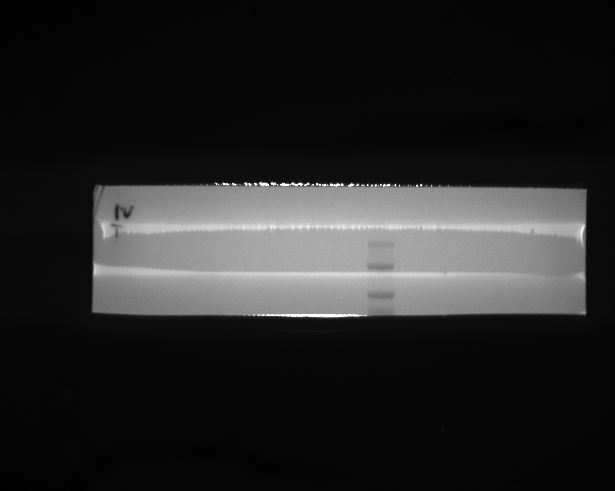

Supplement: Figure 5—source data 1. [file elife-84280-fig5-data1.zip › Figure 5 - Source data 1 - Unedited blots/n6 PGC1a/CHEMI_06302022_184825/CHEMI_06302022_184825_(Membrane)_raw.tif]

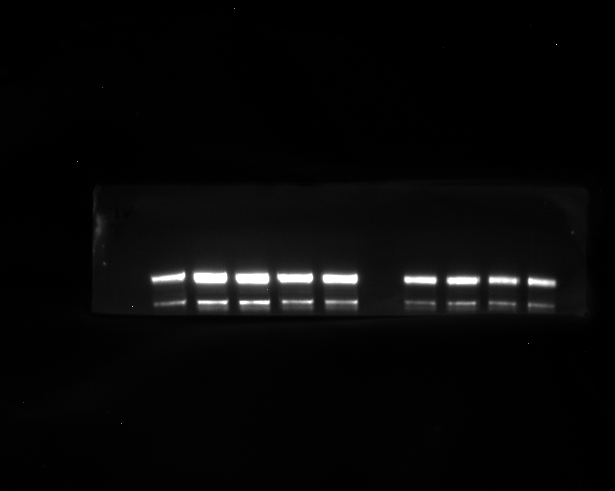

Supplement: Figure 5—source data 1. [file elife-84280-fig5-data1.zip › Figure 5 - Source data 1 - Unedited blots/n6 PGC1a/CHEMI_06302022_184840/CHEMI_06302022_184840_(Chemi)_raw.tif]

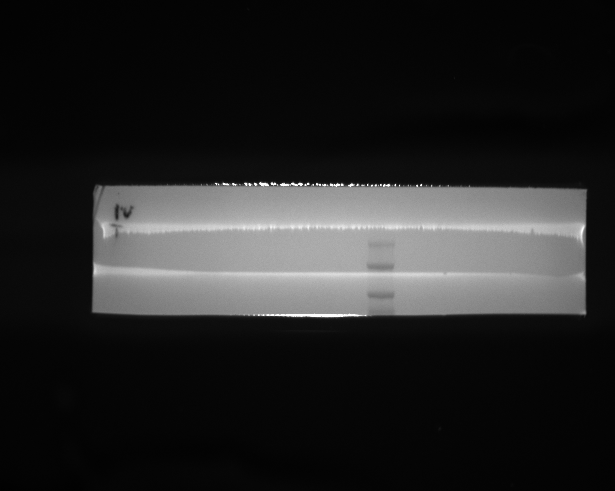

Supplement: Figure 5—source data 1. [file elife-84280-fig5-data1.zip › Figure 5 - Source data 1 - Unedited blots/n6 PGC1a/CHEMI_06302022_184840/CHEMI_06302022_184840_(Membrane)_raw.tif]

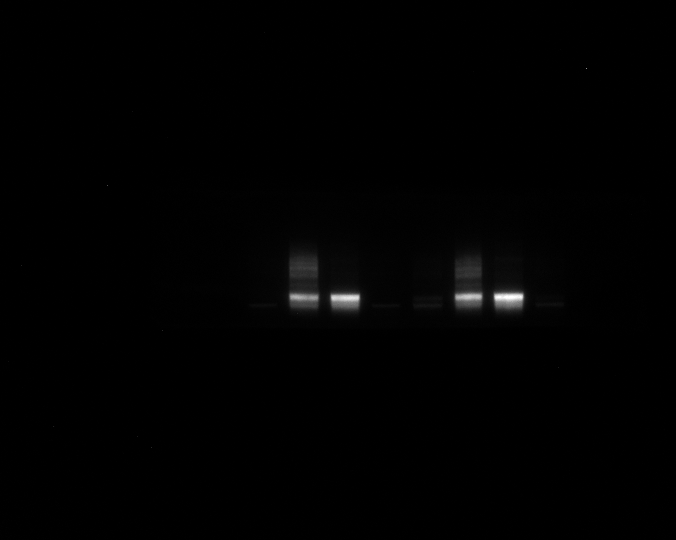

Supplement: Figure 5—source data 1. [file elife-84280-fig5-data1.zip › Figure 5 - Source data 1 - Unedited blots/n7-8 HIF1/CHEMI_06292022_201318_(Chemi)_raw.tif]

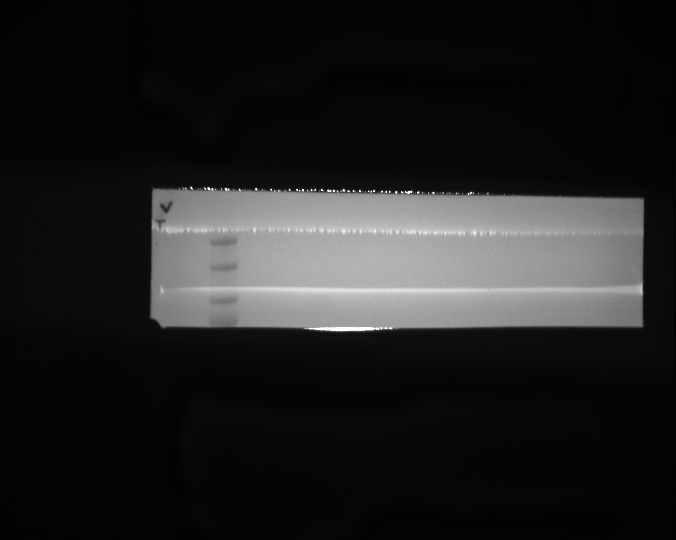

Supplement: Figure 5—source data 1. [file elife-84280-fig5-data1.zip › Figure 5 - Source data 1 - Unedited blots/n7-8 HIF1/CHEMI_06292022_201318_(Membrane)_raw.tif]

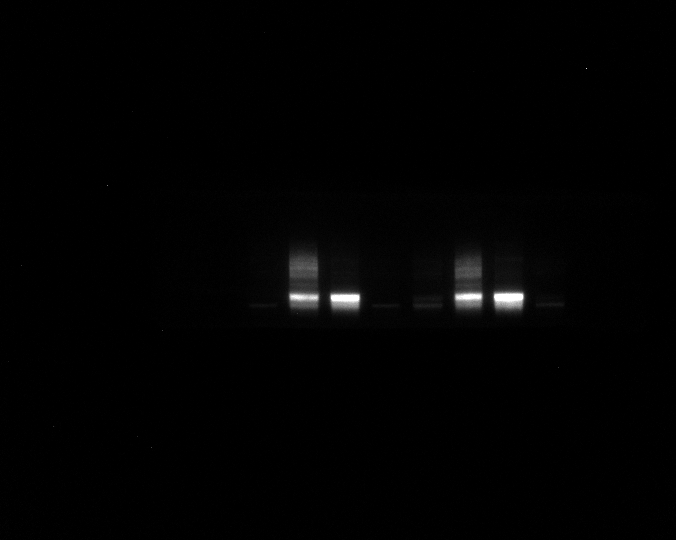

Supplement: Figure 5—source data 1. [file elife-84280-fig5-data1.zip › Figure 5 - Source data 1 - Unedited blots/n7-8 HIF1/CHEMI_06292022_201340_(Chemi)_raw.tif]

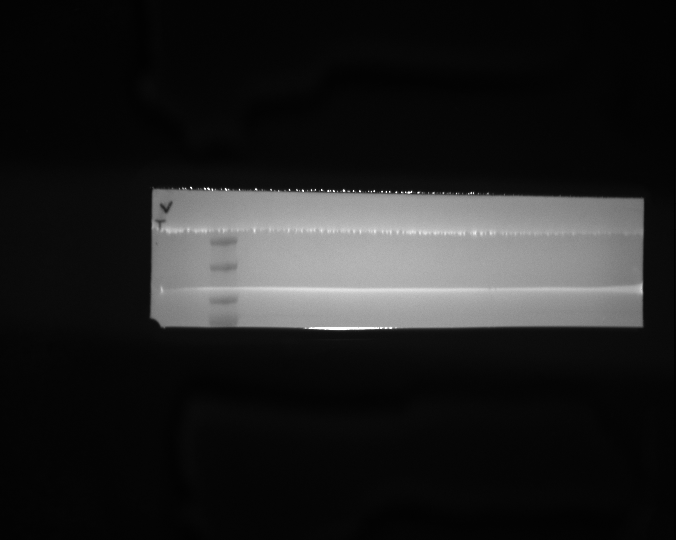

Supplement: Figure 5—source data 1. [file elife-84280-fig5-data1.zip › Figure 5 - Source data 1 - Unedited blots/n7-8 HIF1/CHEMI_06292022_201340_(Membrane)_raw.tif]

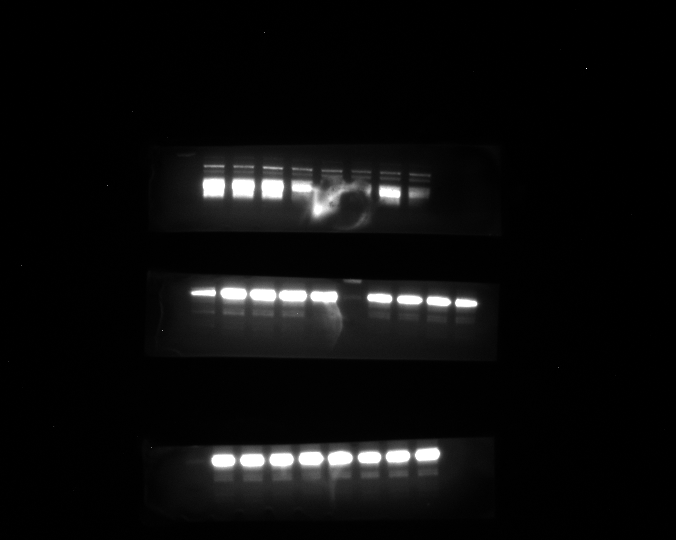

Supplement: Figure 5—source data 1. [file elife-84280-fig5-data1.zip › Figure 5 - Source data 1 - Unedited blots/n7-8 LMNB1/CHEMI_06302022_182914/CHEMI_06302022_182914_(Chemi)_raw.tif]

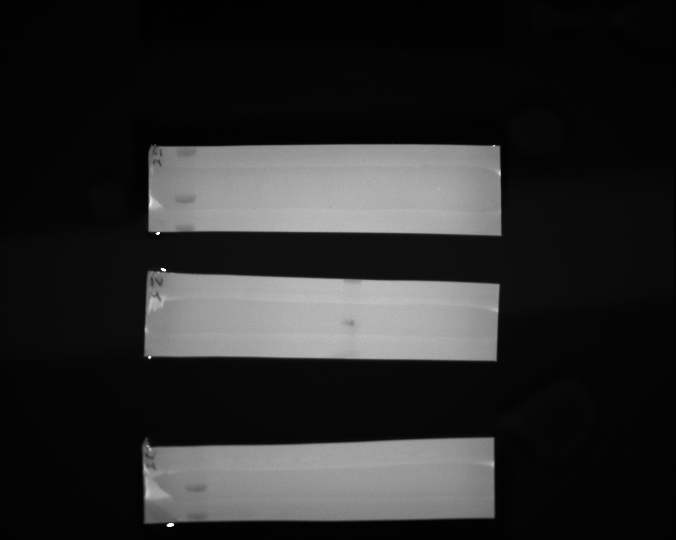

Supplement: Figure 5—source data 1. [file elife-84280-fig5-data1.zip › Figure 5 - Source data 1 - Unedited blots/n7-8 LMNB1/CHEMI_06302022_182914/CHEMI_06302022_182914_(Membrane)_raw.tif]

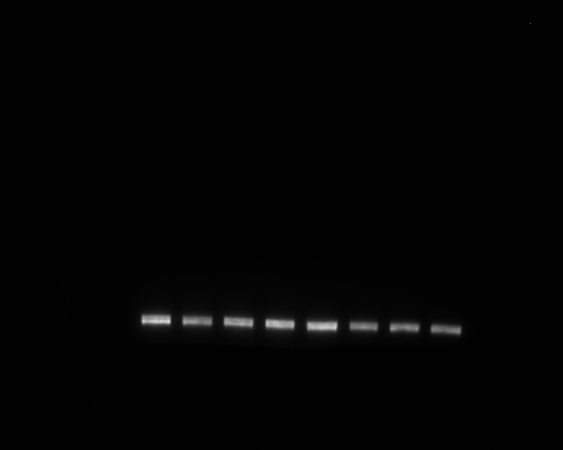

Supplement: Figure 5—source data 1. [file elife-84280-fig5-data1.zip › Figure 5 - Source data 1 - Unedited blots/n7-8 nuclear NFAT/CHEMI_06302022_183807/CHEMI_06302022_183807_(Chemi)_raw.tif]

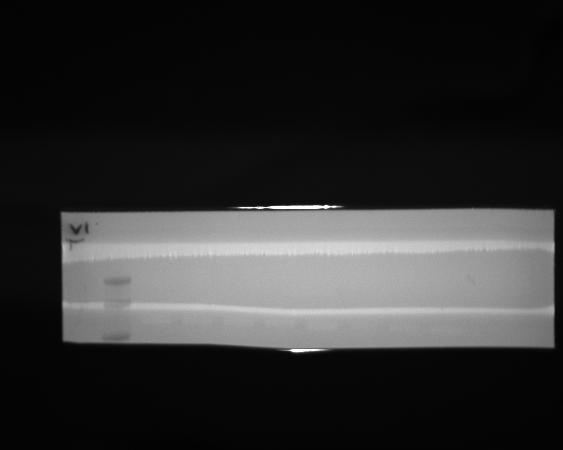

Supplement: Figure 5—source data 1. [file elife-84280-fig5-data1.zip › Figure 5 - Source data 1 - Unedited blots/n7-8 nuclear NFAT/CHEMI_06302022_183807/CHEMI_06302022_183807_(Membrane)_raw.tif]

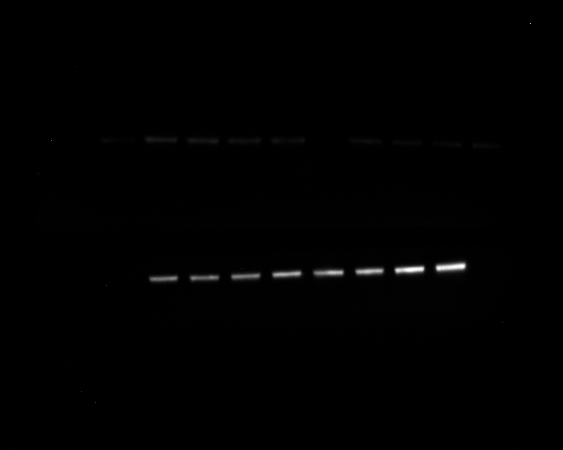

Supplement: Figure 5—source data 1. [file elife-84280-fig5-data1.zip › Figure 5 - Source data 1 - Unedited blots/n7-8 nuclear NFkB/CHEMI_06292022_202045_(Chemi)_raw.tif]

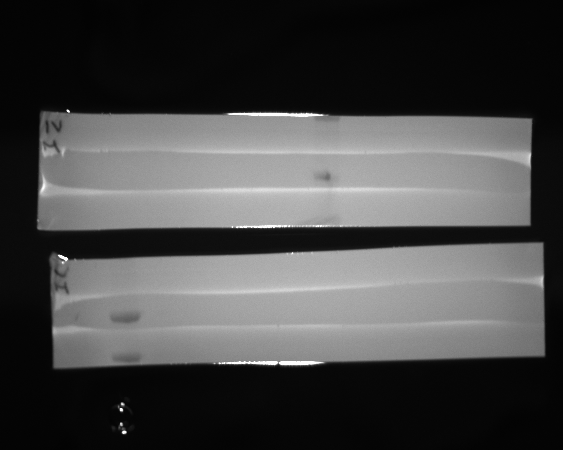

Supplement: Figure 5—source data 1. [file elife-84280-fig5-data1.zip › Figure 5 - Source data 1 - Unedited blots/n7-8 nuclear NFkB/CHEMI_06292022_202045_(Membrane)_raw.tif]

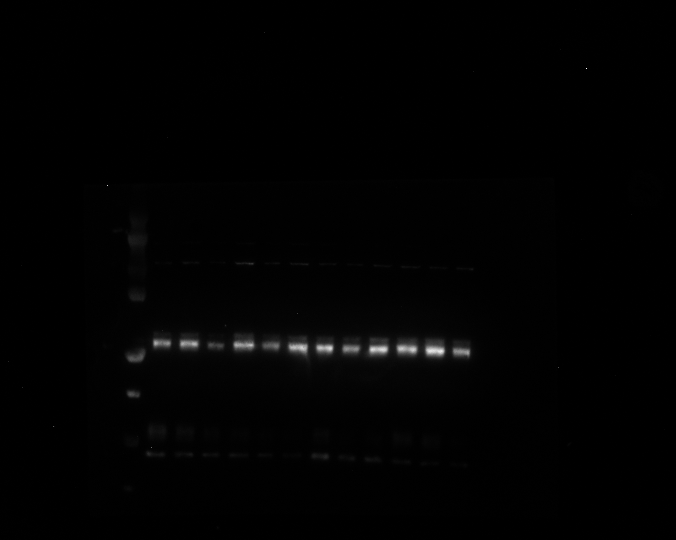

Supplement: Figure 5—source data 1. [file elife-84280-fig5-data1.zip › Figure 5 - Source data 1 - Unedited blots/n9-12 cMyc/nc_2_cmyc/nc_2_cmyc_(Chemi)_raw.tif]

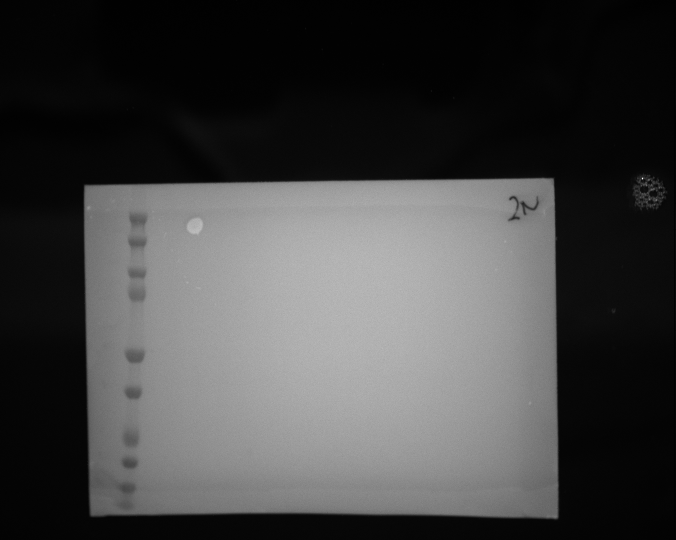

Supplement: Figure 5—source data 1. [file elife-84280-fig5-data1.zip › Figure 5 - Source data 1 - Unedited blots/n9-12 cMyc/nc_2_cmyc/nc_2_cmyc_(Membrane)_raw.tif]

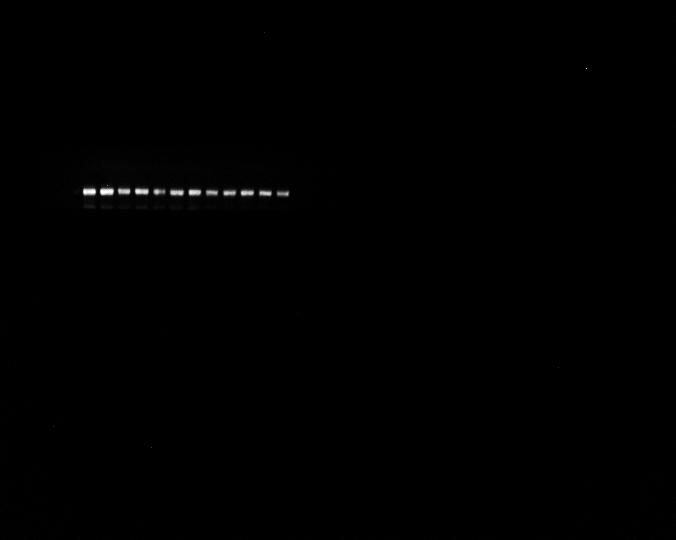

Supplement: Figure 5—source data 1. [file elife-84280-fig5-data1.zip › Figure 5 - Source data 1 - Unedited blots/n9-12 nuclear NFAT/nc_1a_nfat_imag2/nc_1a_nfat_imag2_(Chemi)_raw.tif]

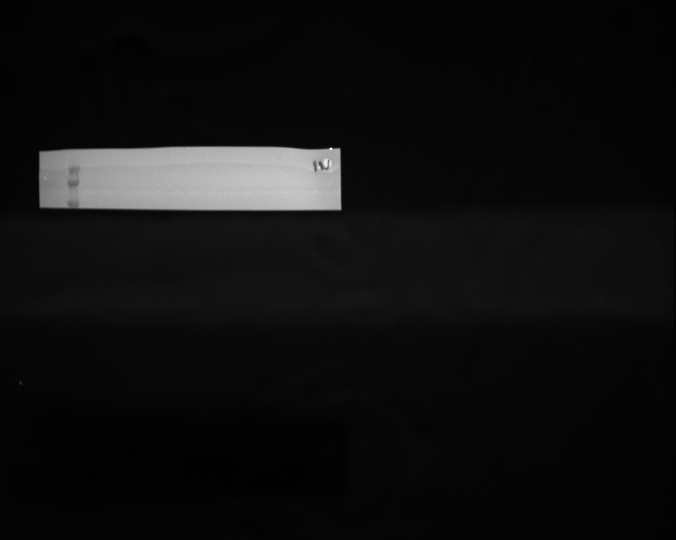

Supplement: Figure 5—source data 1. [file elife-84280-fig5-data1.zip › Figure 5 - Source data 1 - Unedited blots/n9-12 nuclear NFAT/nc_1a_nfat_imag2/nc_1a_nfat_imag2_(Membrane)_raw.tif]

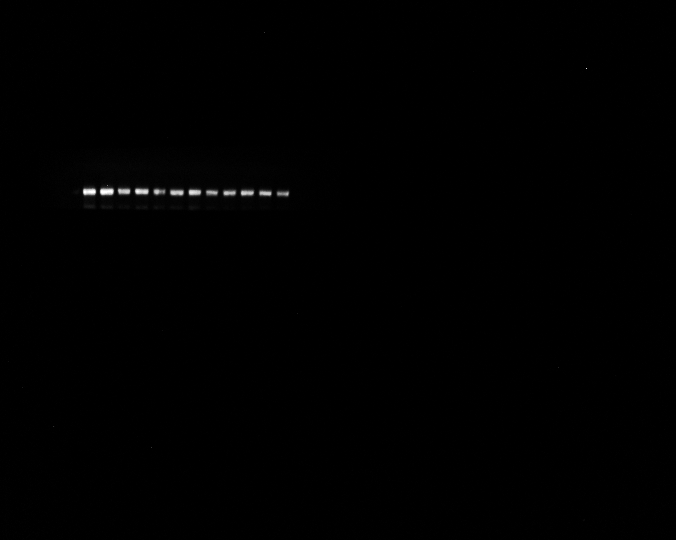

Supplement: Figure 5—source data 1. [file elife-84280-fig5-data1.zip › Figure 5 - Source data 1 - Unedited blots/n9-12 nuclear NFAT/nc_1a_nfat_image1/nc_1a_nfat_image1_(Chemi)_raw.tif]

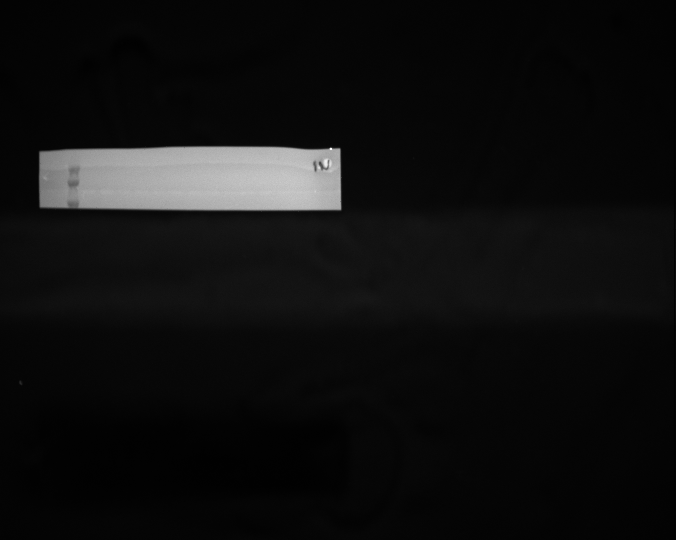

Supplement: Figure 5—source data 1. [file elife-84280-fig5-data1.zip › Figure 5 - Source data 1 - Unedited blots/n9-12 nuclear NFAT/nc_1a_nfat_image1/nc_1a_nfat_image1_(Membrane)_raw.tif]

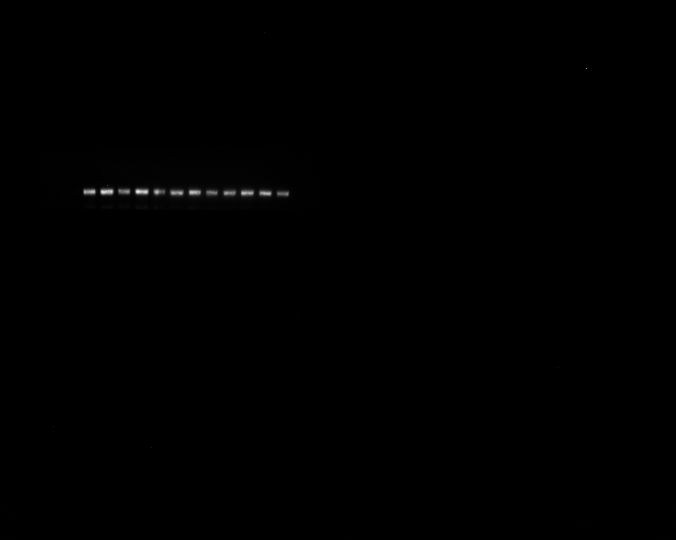

Supplement: Figure 5—source data 1. [file elife-84280-fig5-data1.zip › Figure 5 - Source data 1 - Unedited blots/n9-12 nuclear NFAT/nc_1a_nfat_image4/nc_1a_nfat_image4_(Chemi)_raw.tif]

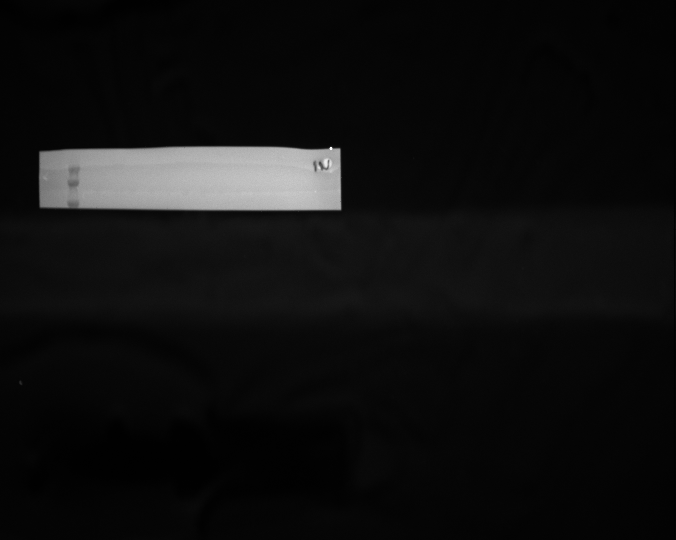

Supplement: Figure 5—source data 1. [file elife-84280-fig5-data1.zip › Figure 5 - Source data 1 - Unedited blots/n9-12 nuclear NFAT/nc_1a_nfat_image4/nc_1a_nfat_image4_(Membrane)_raw.tif]

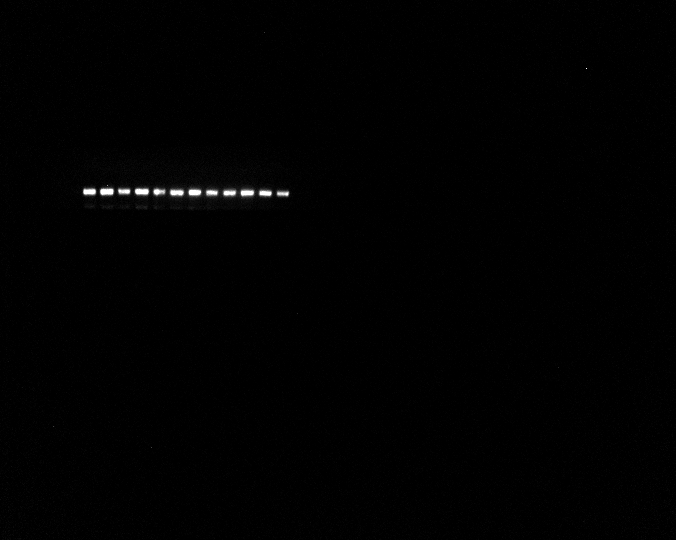

Supplement: Figure 5—source data 1. [file elife-84280-fig5-data1.zip › Figure 5 - Source data 1 - Unedited blots/n9-12 nuclear NFAT/nc_1a_nfat_image5/nc_1a_nfat_image5_(Chemi)_raw.tif]

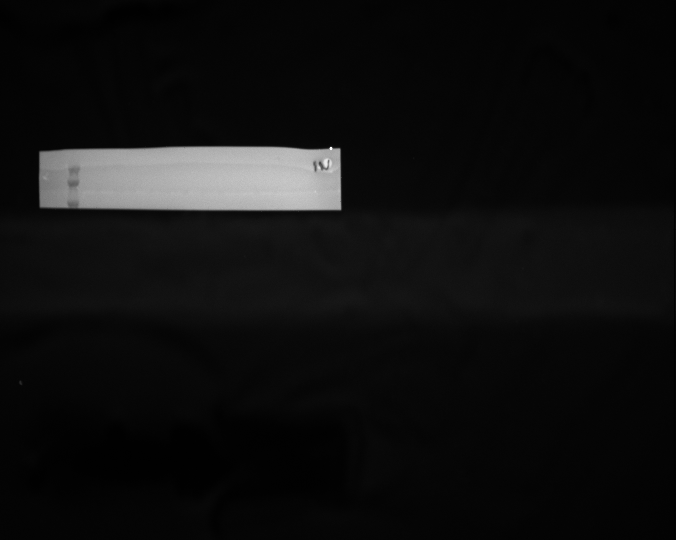

Supplement: Figure 5—source data 1. [file elife-84280-fig5-data1.zip › Figure 5 - Source data 1 - Unedited blots/n9-12 nuclear NFAT/nc_1a_nfat_image5/nc_1a_nfat_image5_(Membrane)_raw.tif]

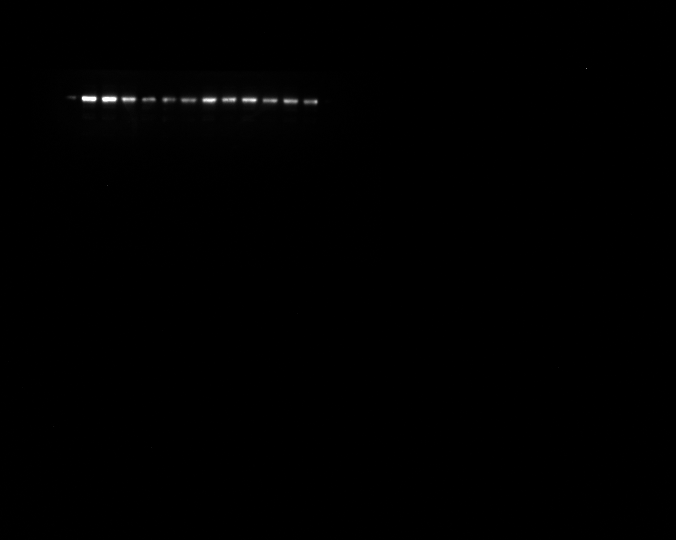

Supplement: Figure 5—source data 1. [file elife-84280-fig5-data1.zip › Figure 5 - Source data 1 - Unedited blots/n9-12 nuclear NFkB/nc_1b_nfkb_image1/nc_1b_nfkb_image1_(Chemi)_raw.tif]

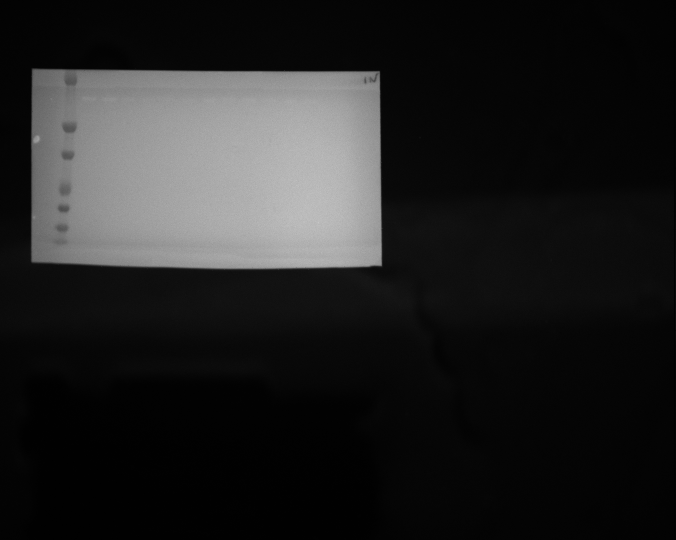

Supplement: Figure 5—source data 1. [file elife-84280-fig5-data1.zip › Figure 5 - Source data 1 - Unedited blots/n9-12 nuclear NFkB/nc_1b_nfkb_image1/nc_1b_nfkb_image1_(Membrane)_raw.tif]

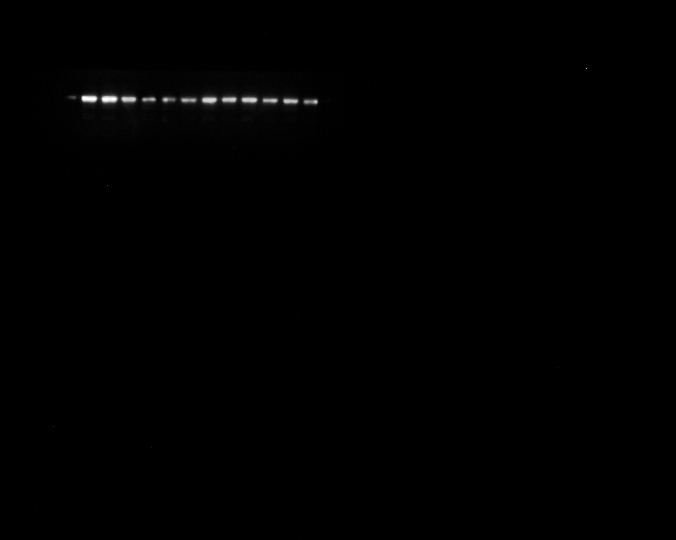

Supplement: Figure 5—source data 1. [file elife-84280-fig5-data1.zip › Figure 5 - Source data 1 - Unedited blots/n9-12 nuclear NFkB/nc_1b_nfkb_image2/nc_1b_nfkb_image2_(Chemi)_raw.tif]

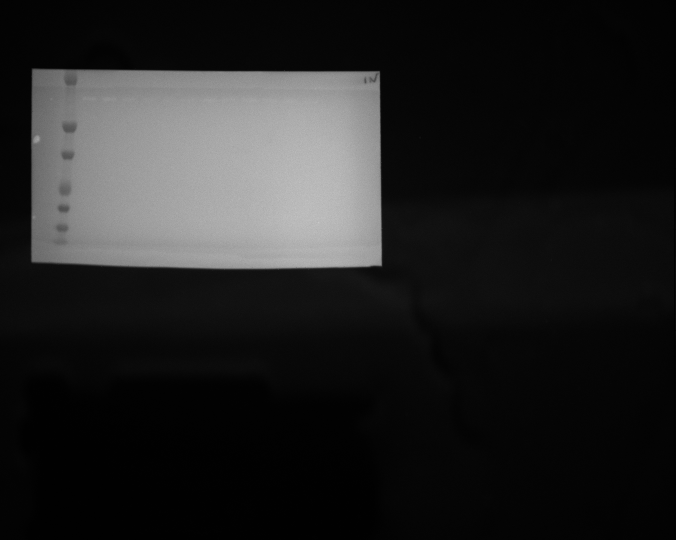

Supplement: Figure 5—source data 1. [file elife-84280-fig5-data1.zip › Figure 5 - Source data 1 - Unedited blots/n9-12 nuclear NFkB/nc_1b_nfkb_image2/nc_1b_nfkb_image2_(Membrane)_raw.tif]

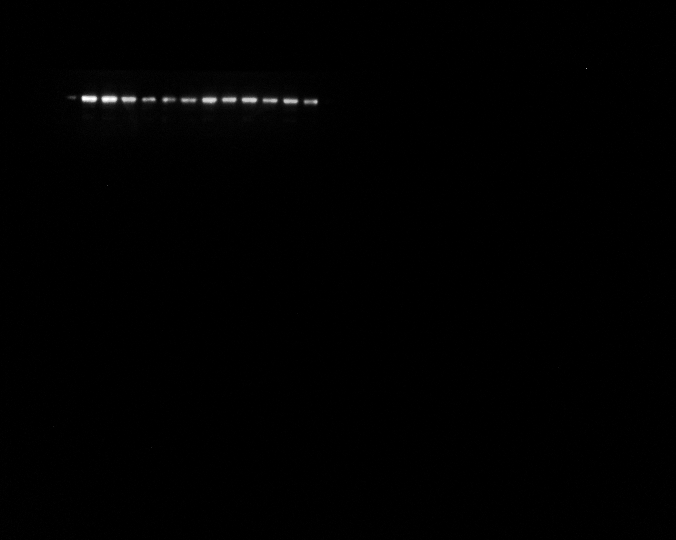

Supplement: Figure 5—source data 1. [file elife-84280-fig5-data1.zip › Figure 5 - Source data 1 - Unedited blots/n9-12 nuclear NFkB/nc_1b_nfkb_image3/nc_1b_nfkb_image3_(Chemi)_raw.tif]

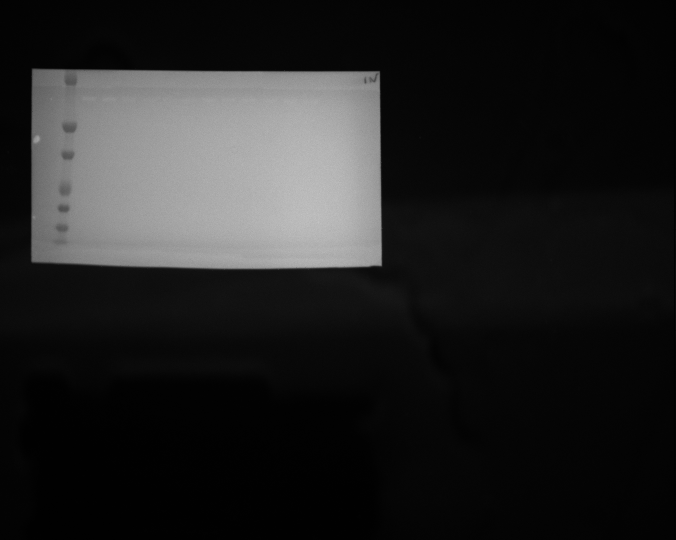

Supplement: Figure 5—source data 1. [file elife-84280-fig5-data1.zip › Figure 5 - Source data 1 - Unedited blots/n9-12 nuclear NFkB/nc_1b_nfkb_image3/nc_1b_nfkb_image3_(Membrane)_raw.tif]

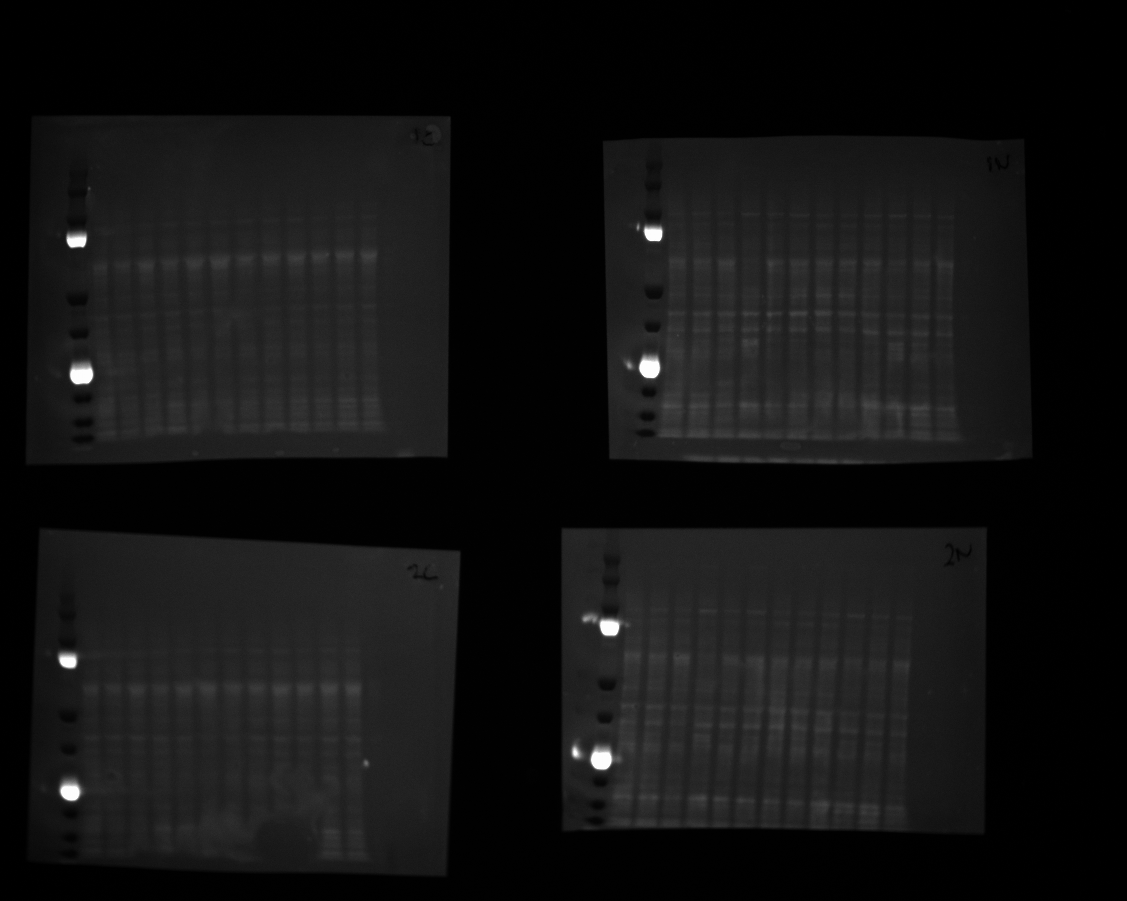

Supplement: Figure 5—source data 1. [file elife-84280-fig5-data1.zip › Figure 5 - Source data 1 - Unedited blots/n9-12 TPS (top right NFAT and NFkb, bottom right for cMyc)/UNIVERSAL_06012022_193956_(1_No-Stain Labeled Membrane)_raw.tif]

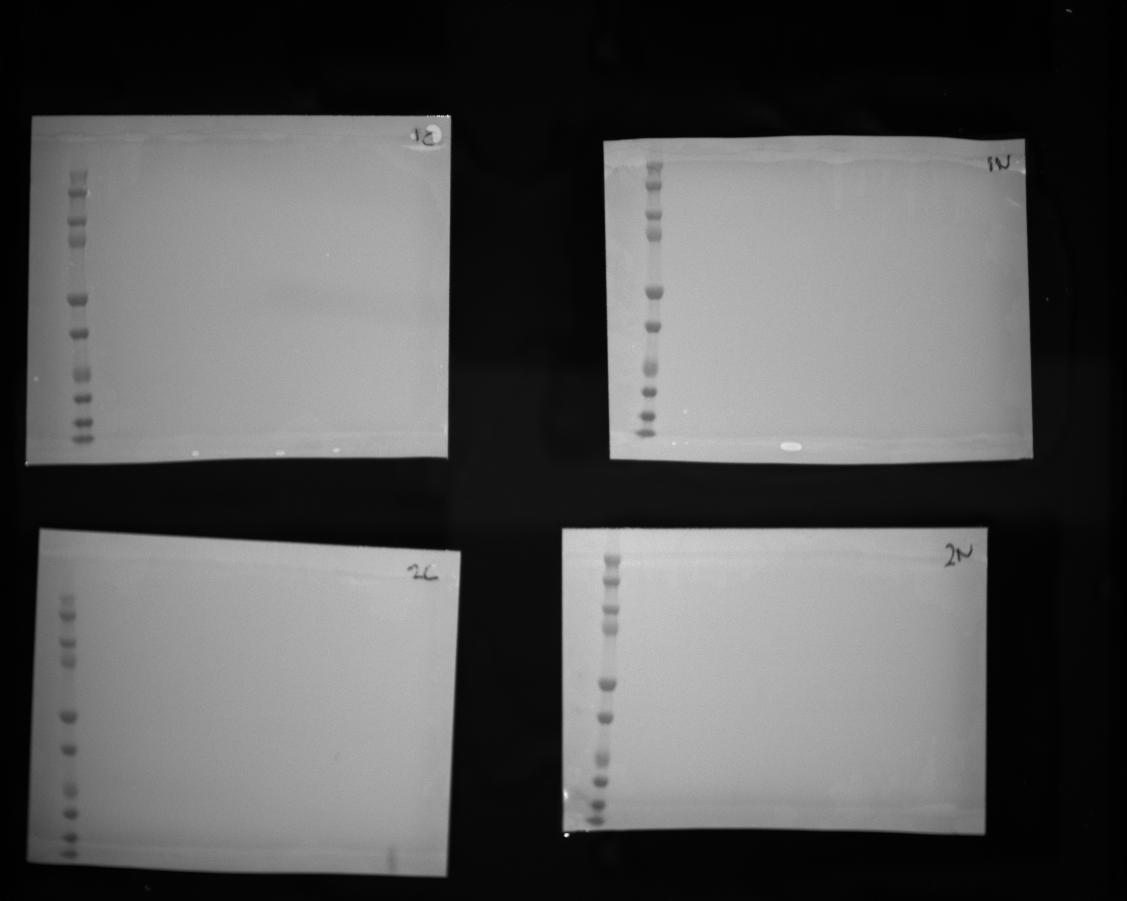

Supplement: Figure 5—source data 1. [file elife-84280-fig5-data1.zip › Figure 5 - Source data 1 - Unedited blots/n9-12 TPS (top right NFAT and NFkb, bottom right for cMyc)/UNIVERSAL_06012022_193956_(Membrane)_raw.tif]

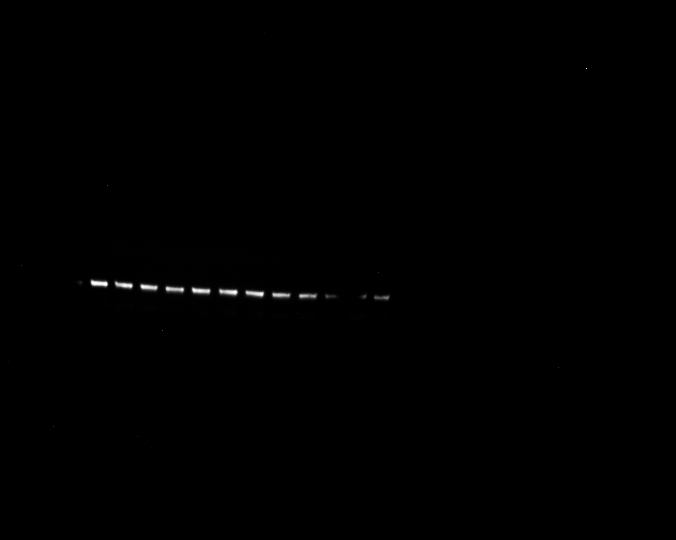

Supplement: Figure 5—figure supplement 1—source data 1. [file elife-84280-fig5-figsupp1-data1.zip › Fig 5 - fig S1 - Source data - Unedited blots/Figure 5 - figure supplement 1E/Cytoplasmic NFAT/a1_cyt_nfat_2_(Chemi)_raw.tif]

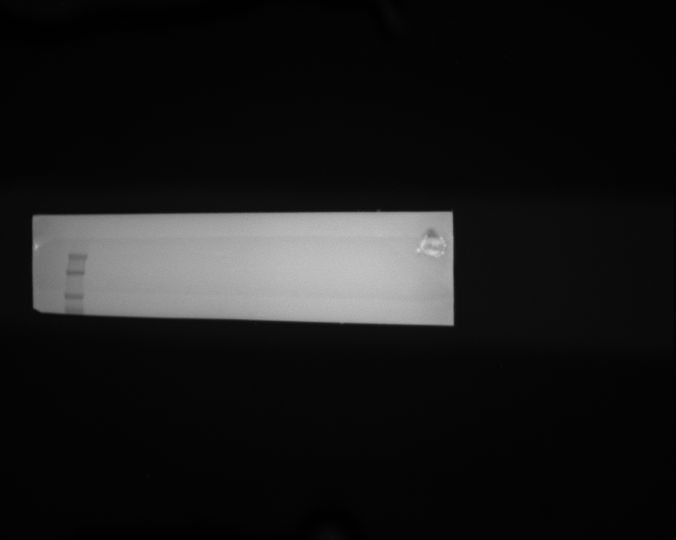

Supplement: Figure 5—figure supplement 1—source data 1. [file elife-84280-fig5-figsupp1-data1.zip › Fig 5 - fig S1 - Source data - Unedited blots/Figure 5 - figure supplement 1E/Cytoplasmic NFAT/a1_cyt_nfat_2_(Membrane)_raw.tif]

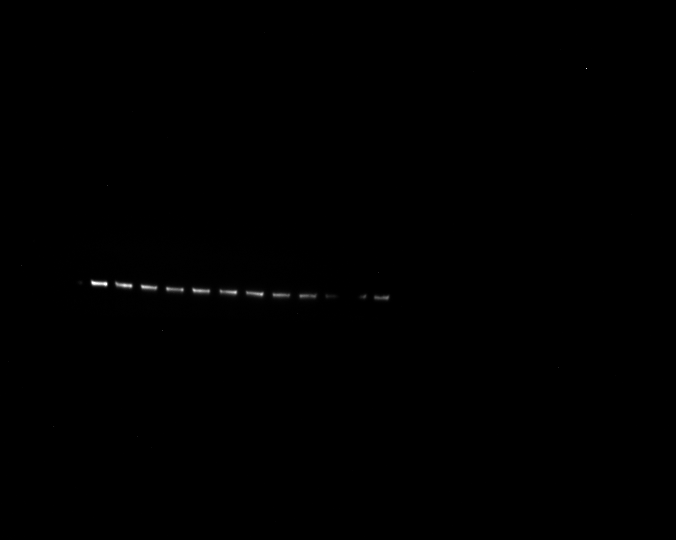

Supplement: Figure 5—figure supplement 1—source data 1. [file elife-84280-fig5-figsupp1-data1.zip › Fig 5 - fig S1 - Source data - Unedited blots/Figure 5 - figure supplement 1E/Cytoplasmic NFAT/a1_cyti_nfat_4_(Chemi)_raw.tif]

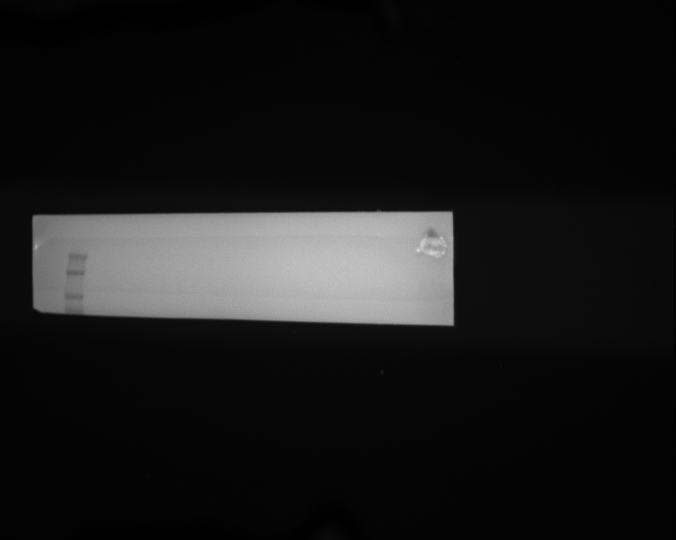

Supplement: Figure 5—figure supplement 1—source data 1. [file elife-84280-fig5-figsupp1-data1.zip › Fig 5 - fig S1 - Source data - Unedited blots/Figure 5 - figure supplement 1E/Cytoplasmic NFAT/a1_cyti_nfat_4_(Membrane)_raw.tif]

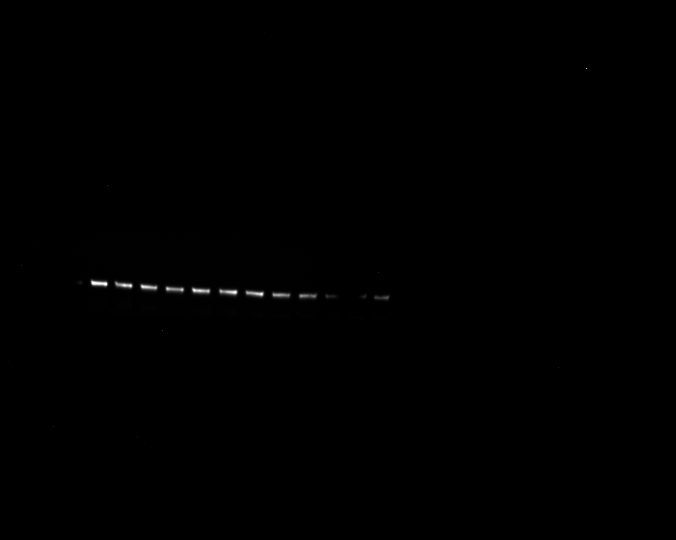

Supplement: Figure 5—figure supplement 1—source data 1. [file elife-84280-fig5-figsupp1-data1.zip › Fig 5 - fig S1 - Source data - Unedited blots/Figure 5 - figure supplement 1E/Cytoplasmic NFAT/a1_cyto_nfat_1_(Chemi)_raw.tif]

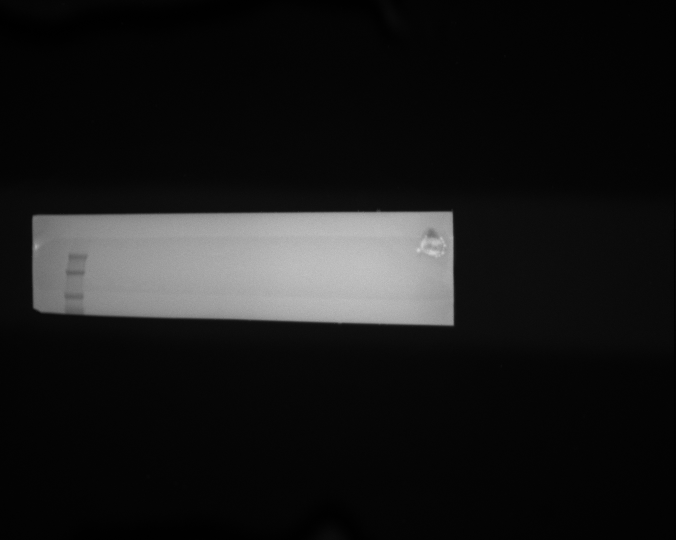

Supplement: Figure 5—figure supplement 1—source data 1. [file elife-84280-fig5-figsupp1-data1.zip › Fig 5 - fig S1 - Source data - Unedited blots/Figure 5 - figure supplement 1E/Cytoplasmic NFAT/a1_cyto_nfat_1_(Membrane)_raw.tif]

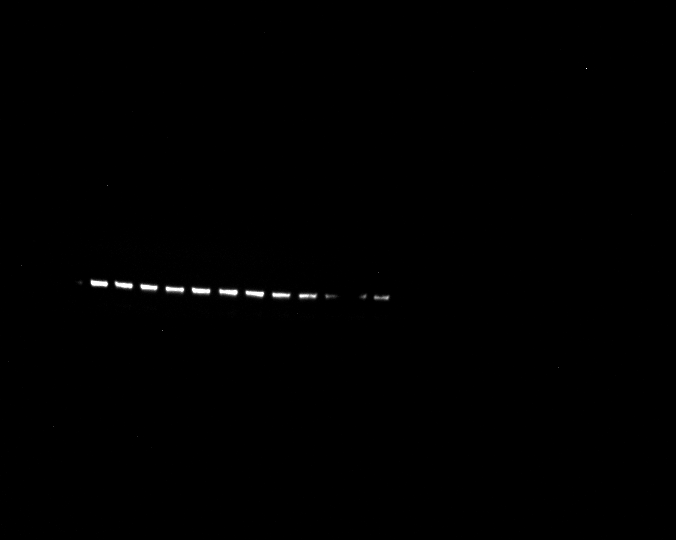

Supplement: Figure 5—figure supplement 1—source data 1. [file elife-84280-fig5-figsupp1-data1.zip › Fig 5 - fig S1 - Source data - Unedited blots/Figure 5 - figure supplement 1E/Cytoplasmic NFAT/a1_cyto_nfat_3_(Chemi)_raw.tif]

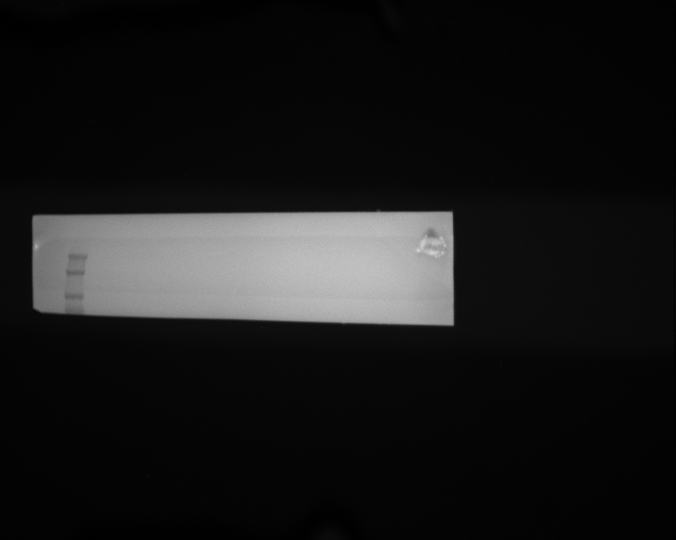

Supplement: Figure 5—figure supplement 1—source data 1. [file elife-84280-fig5-figsupp1-data1.zip › Fig 5 - fig S1 - Source data - Unedited blots/Figure 5 - figure supplement 1E/Cytoplasmic NFAT/a1_cyto_nfat_3_(Membrane)_raw.tif]

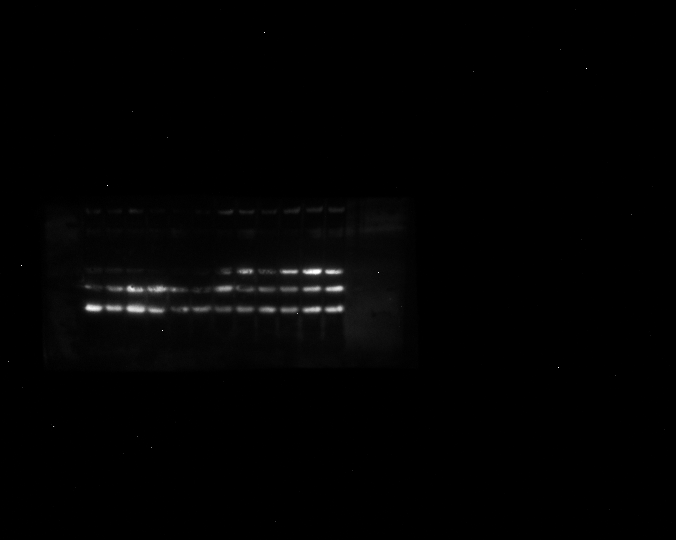

Supplement: Figure 5—figure supplement 1—source data 1. [file elife-84280-fig5-figsupp1-data1.zip › Fig 5 - fig S1 - Source data - Unedited blots/Figure 5 - figure supplement 1E/Histone 3/b1_h3(Chemi)_raw.tif]

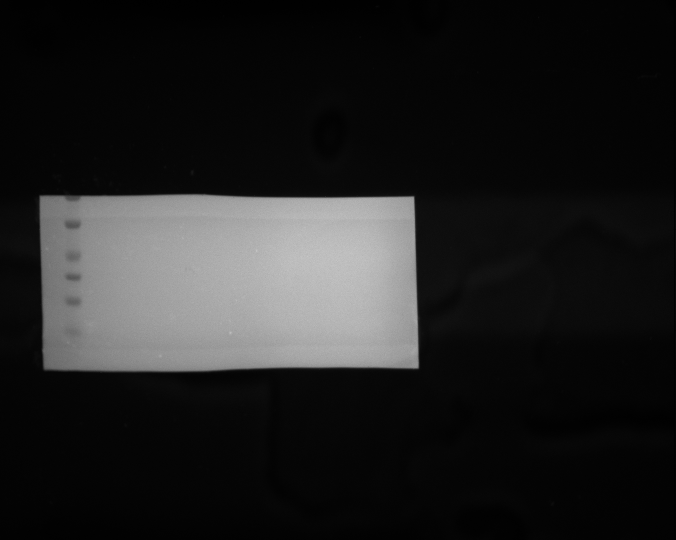

Supplement: Figure 5—figure supplement 1—source data 1. [file elife-84280-fig5-figsupp1-data1.zip › Fig 5 - fig S1 - Source data - Unedited blots/Figure 5 - figure supplement 1E/Histone 3/b1_h3(Membrane)_raw.tif]

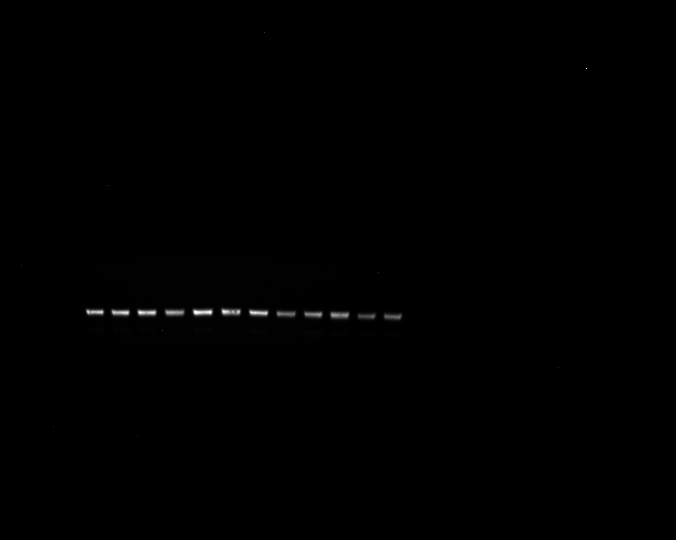

Supplement: Figure 5—figure supplement 1—source data 1. [file elife-84280-fig5-figsupp1-data1.zip › Fig 5 - fig S1 - Source data - Unedited blots/Figure 5 - figure supplement 1E/Nuclear NFAT/b1_nx_nfat_1_(Chemi)_raw.tif]

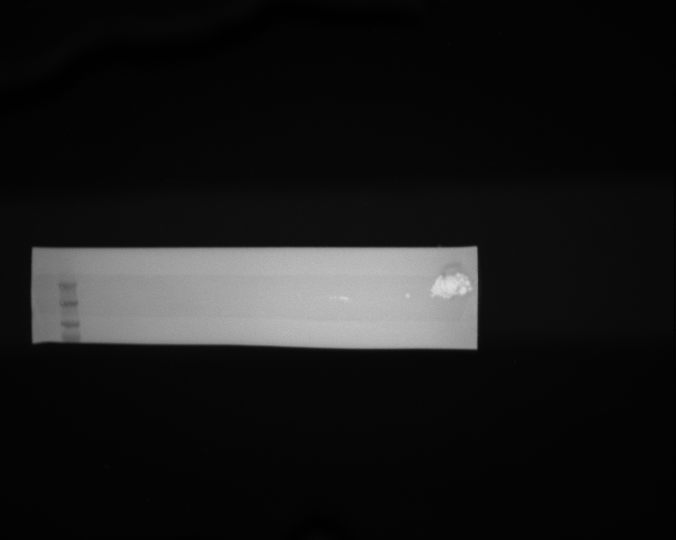

Supplement: Figure 5—figure supplement 1—source data 1. [file elife-84280-fig5-figsupp1-data1.zip › Fig 5 - fig S1 - Source data - Unedited blots/Figure 5 - figure supplement 1E/Nuclear NFAT/b1_nx_nfat_1_(Membrane)_raw.tif]

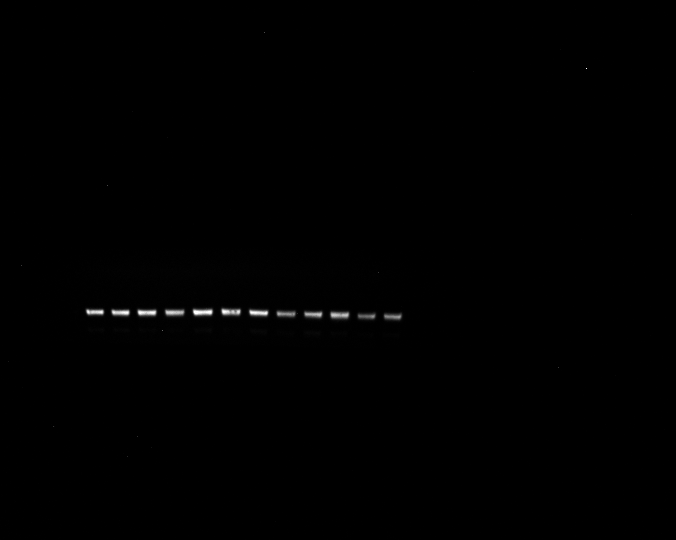

Supplement: Figure 5—figure supplement 1—source data 1. [file elife-84280-fig5-figsupp1-data1.zip › Fig 5 - fig S1 - Source data - Unedited blots/Figure 5 - figure supplement 1E/Nuclear NFAT/b1_nx_nfat_2_(Chemi)_raw.tif]

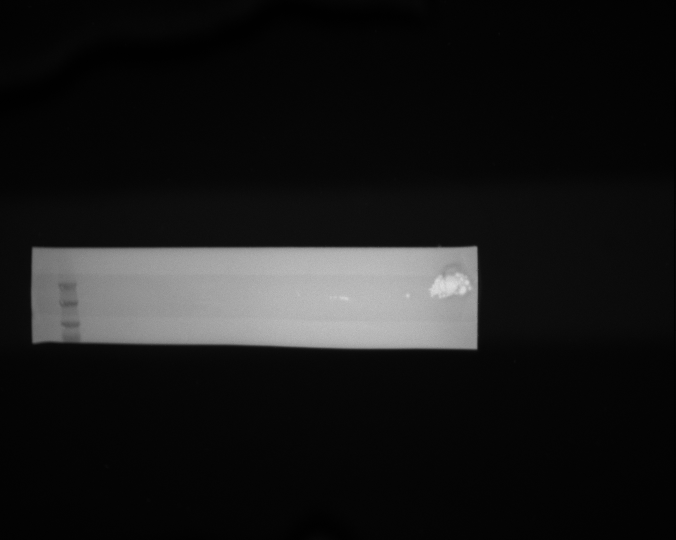

Supplement: Figure 5—figure supplement 1—source data 1. [file elife-84280-fig5-figsupp1-data1.zip › Fig 5 - fig S1 - Source data - Unedited blots/Figure 5 - figure supplement 1E/Nuclear NFAT/b1_nx_nfat_2_(Membrane)_raw.tif]

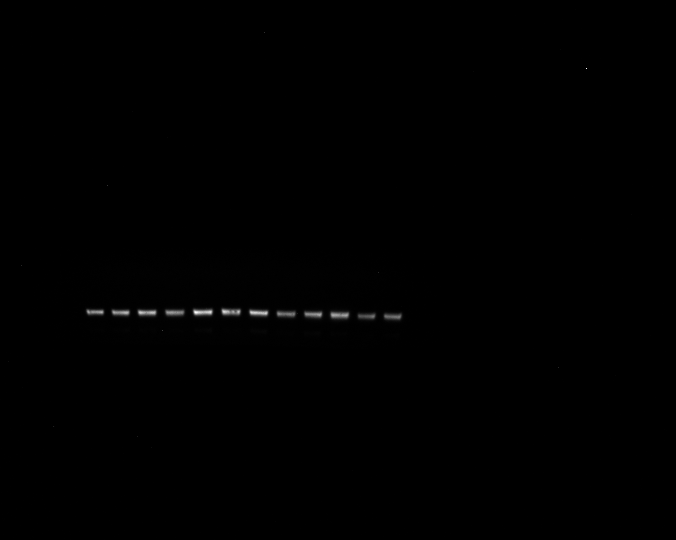

Supplement: Figure 5—figure supplement 1—source data 1. [file elife-84280-fig5-figsupp1-data1.zip › Fig 5 - fig S1 - Source data - Unedited blots/Figure 5 - figure supplement 1E/Nuclear NFAT/b1_nx_nfat_3_(Chemi)_raw.tif]

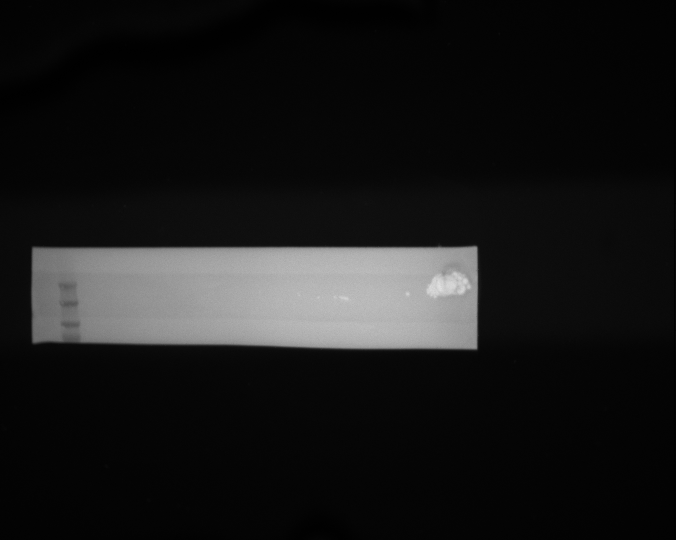

Supplement: Figure 5—figure supplement 1—source data 1. [file elife-84280-fig5-figsupp1-data1.zip › Fig 5 - fig S1 - Source data - Unedited blots/Figure 5 - figure supplement 1E/Nuclear NFAT/b1_nx_nfat_3_(Membrane)_raw.tif]

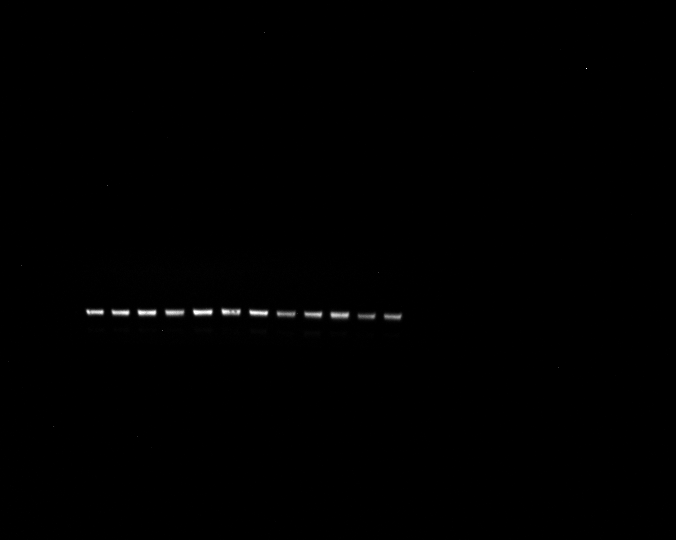

Supplement: Figure 5—figure supplement 1—source data 1. [file elife-84280-fig5-figsupp1-data1.zip › Fig 5 - fig S1 - Source data - Unedited blots/Figure 5 - figure supplement 1E/Nuclear NFAT/b1_nx_nfat_4_(Chemi)_raw.tif]

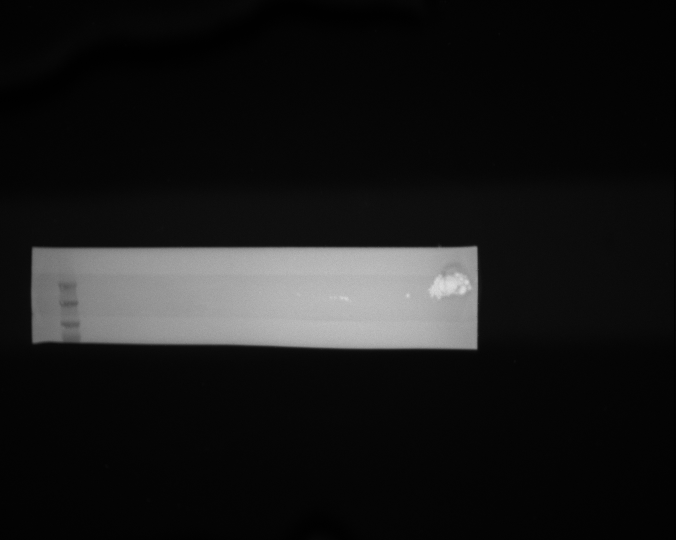

Supplement: Figure 5—figure supplement 1—source data 1. [file elife-84280-fig5-figsupp1-data1.zip › Fig 5 - fig S1 - Source data - Unedited blots/Figure 5 - figure supplement 1E/Nuclear NFAT/b1_nx_nfat_4_(Membrane)_raw.tif]

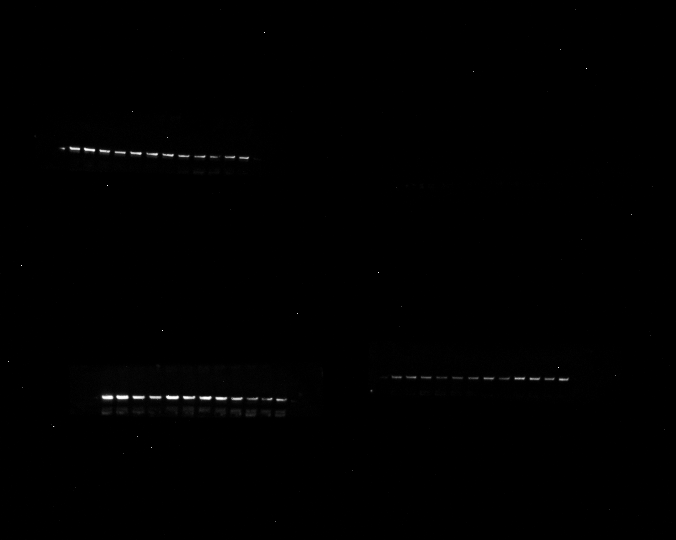

Supplement: Figure 5—figure supplement 1—source data 1. [file elife-84280-fig5-figsupp1-data1.zip › Fig 5 - fig S1 - Source data - Unedited blots/Figure 5 - figure supplement 1E/Vinculin (top left)/vis_1_(Chemi)_raw.tif]

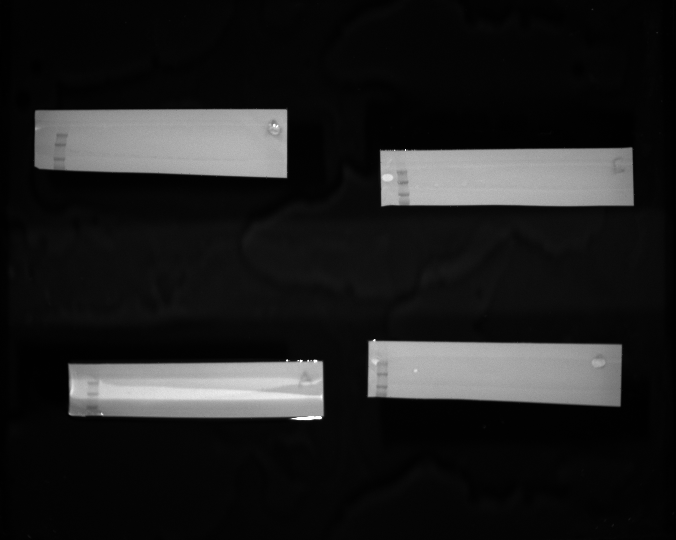

Supplement: Figure 5—figure supplement 1—source data 1. [file elife-84280-fig5-figsupp1-data1.zip › Fig 5 - fig S1 - Source data - Unedited blots/Figure 5 - figure supplement 1E/Vinculin (top left)/vis_1_(Membrane)_raw.tif]

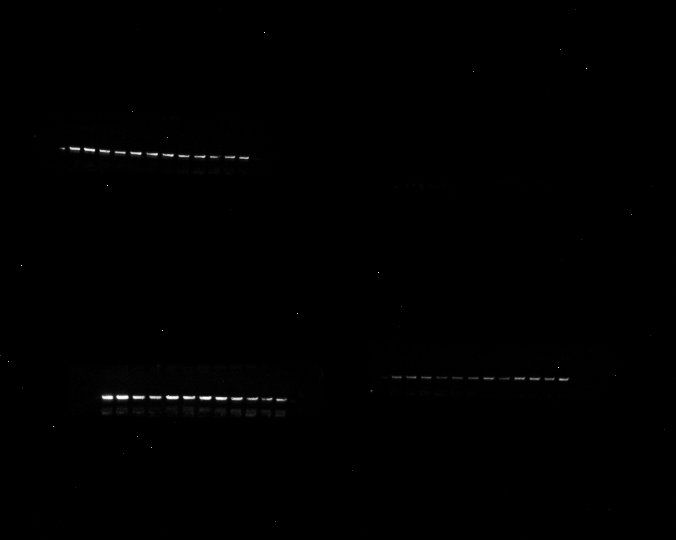

Supplement: Figure 5—figure supplement 1—source data 1. [file elife-84280-fig5-figsupp1-data1.zip › Fig 5 - fig S1 - Source data - Unedited blots/Figure 5 - figure supplement 1E/Vinculin (top left)/vis_2_(Chemi)_raw.tif]

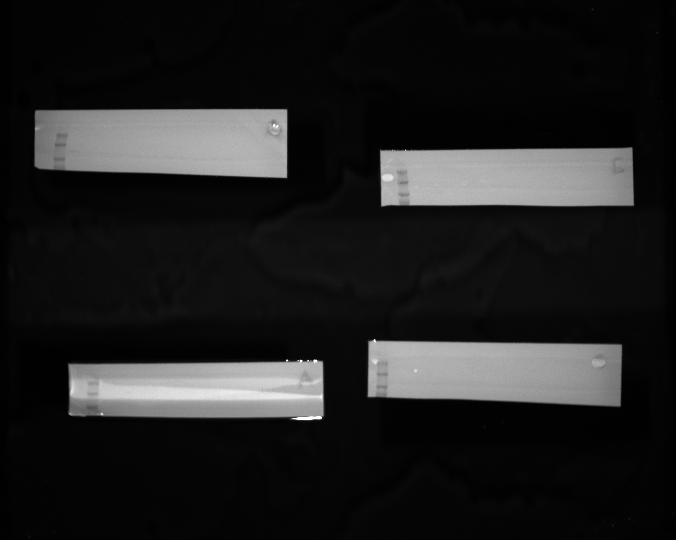

Supplement: Figure 5—figure supplement 1—source data 1. [file elife-84280-fig5-figsupp1-data1.zip › Fig 5 - fig S1 - Source data - Unedited blots/Figure 5 - figure supplement 1E/Vinculin (top left)/vis_2_(Membrane)_raw.tif]

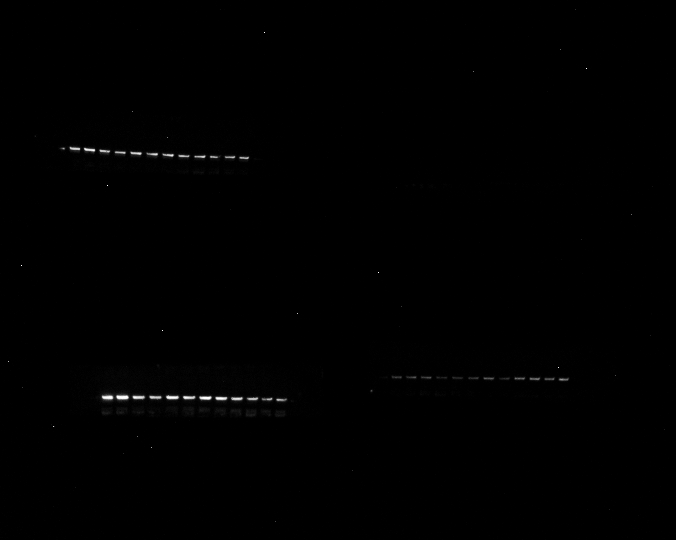

Supplement: Figure 5—figure supplement 1—source data 1. [file elife-84280-fig5-figsupp1-data1.zip › Fig 5 - fig S1 - Source data - Unedited blots/Figure 5 - figure supplement 1E/Vinculin (top left)/vis_3_(Chemi)_raw.tif]

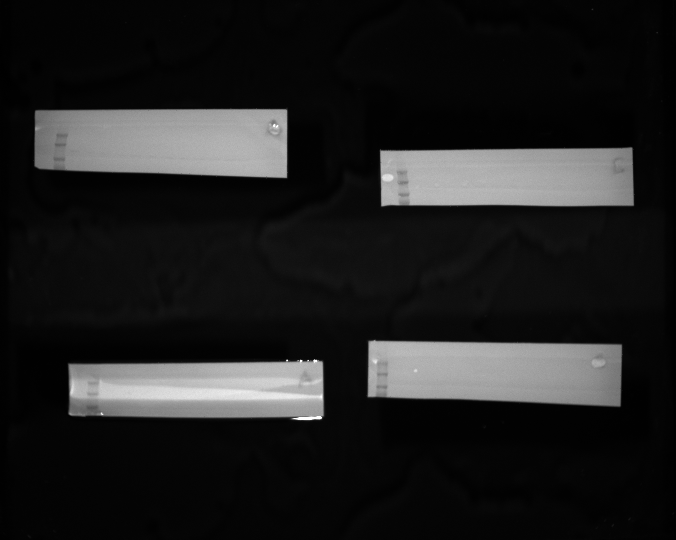

Supplement: Figure 5—figure supplement 1—source data 1. [file elife-84280-fig5-figsupp1-data1.zip › Fig 5 - fig S1 - Source data - Unedited blots/Figure 5 - figure supplement 1E/Vinculin (top left)/vis_3_(Membrane)_raw.tif]

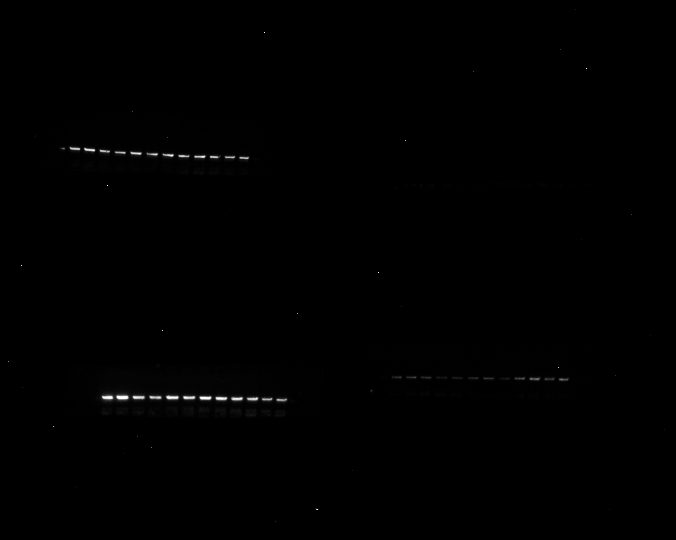

Supplement: Figure 5—figure supplement 1—source data 1. [file elife-84280-fig5-figsupp1-data1.zip › Fig 5 - fig S1 - Source data - Unedited blots/Figure 5 - figure supplement 1E/Vinculin (top left)/vis_4_(Chemi)_raw.tif]

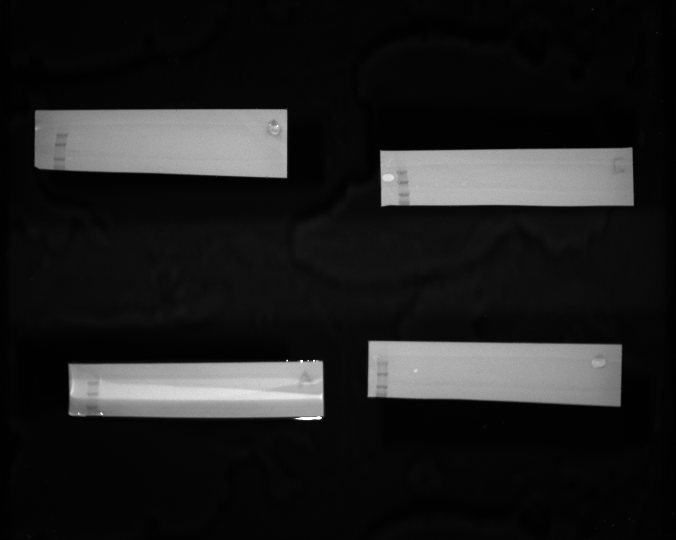

Supplement: Figure 5—figure supplement 1—source data 1. [file elife-84280-fig5-figsupp1-data1.zip › Fig 5 - fig S1 - Source data - Unedited blots/Figure 5 - figure supplement 1E/Vinculin (top left)/vis_4_(Membrane)_raw.tif]
