# Supplementary material for: Oxygen levels at the time of activation determine T cell persistence and immunotherapeutic efficacy
Source: eLife. 2023 May 11;12:e84280. doi: 10.7554/eLife.84280 (PMC10229120; doi:10.7554/eLife.84280)
Supplement: Supplementary file 1. — Species reactivity: mouse (M), human (H), or both (M/H). [file elife-84280-supp1.docx]

##### **Supplementary file 1**. List of antibodies used for flow cytometry. Species reactivity: mouse (M), human (H) or both (M/H).

| **Species** | Target | **Fluorochrome** | **Clone** | **Supplier** | **Catalog #** |
| --- | --- | --- | --- | --- | --- |
| M | **Bcl-2** | **PE/Cy7** | BCL/10C4 | BioLegend | 633511 |
| M/H | **Blimp-1** | **AF647** | 6D3 (RUO) | BD | 565002 |
| H | **CA9** | **PE** | 303123 | R&D | FAB2188P |
| H | **CCR7** | **BUV737** | 3D12 | BD | 741786 |
| H | **CCR7** | **PE** | 3D12 | BD | [552176](https://www.bdbiosciences.com/us/p/552176) |
| H | **CCR7** | **PE/Cy7** | 3D12 | BD | [557648](https://www.bdbiosciences.com/us/applications/research/t-cell-immunology/th-2-cells/surface-markers/human/pe-cy7-rat-anti-human-ccr7-cd197-3d12/p/560922) |
| M | **CD11b** | **AF700** | M1/70 | eBiosciences | 56-0112-80 |
| M | **CD11c** | **PerCP/Cy5.5** | N418 | eBiosciences | 45-0114-80 |
| M | **CD127** | **AF647** | A7R34 | Biolegend | 135020 |
| H | **CD127** | **APC** | A019D5 | Biolegend | 351342 |
| H | **CD127** | **BV421** | A019D5 | Biolegend | 351310 |
| H | **CD127** | **PE** | A019D5 | Biolegend | 351303 |
| M | **CD137** | **APC** | 17B5 | Biolegend | 106110 |
| H | **CD137** | **PE/Cy7** | 4B4-1 | BioLegend | [309817](https://www.biolegend.com/en-gb/products/pe-cyanine7-anti-human-cd137-4-1bb-antibody-6540) |
| M | **CD19** | **APC** | eBio1D3 | eBiosciences | 11-0193-82 |
| H | **CD19** | **APC** | 4G7 | Biolegend | 392504 |
| H | **CD19** | **PE** | 4G7 | Biolegend | 392505 |
| H | **CD25** | **BUV737** | BC96 | Therno | 367-0259-41 |
| H | **CD25** | **BV510** | BC96 | Biolegend | 302639 |
| M | **CD25** | **eFluor 450** | PC61.5 | Thermo | [48-0251](https://www.thermofisher.com/antibody/product/CD25-Antibody-clone-PC61-5-Monoclonal/48-0251-82) |
| M/H | **CD27** | **AF700** | LG.3A10 | Biolegend | 124239 |
| H | **CD27** | **PerCP/Cy5.5** | M-T271 | Biolegend | 356408 |
| M | **CD3** | **PerCP/Cy5.5** | 17A2 | BioLegend | 100218 |
| H | **CD34 (RQR8)** | **AF488** | QBEnd10 | R&D | [FAB7227G](https://www.rndsystems.com/products/human-cd34-alexa-fluor-488-conjugated-antibody-qbend10_fab7227g) |
| H | **CD34 (RQR8)** | **PE** | QBEnd10 | Thermo | MA1-10205 |
| M | **CD4** | **BV421** | GK1.5 | BioLegend | [100437](https://www.biolegend.com/brilliant-violet-421-anti-mouse-cd4-antibody-7142.html) |
| M/H | **CD44** | **PE/Cy7** | IM7 | BioLegend | [103030](https://www.biolegend.com/en-us/products/pe-cy7-anti-mouse-human-cd44-antibody-3932) |
| H | **CD45** | **PE** | HI30 | BioLegend | 304007 |
| M | **CD45.1** | **AF647** | A20 | BioLegend | 110720 |
| M | **CD45.1** | **BV421** | A20 | BD | [563983](https://www.bdbiosciences.com/us/p/563983) |
| M | **CD45.2** | **AF647** | 104 | BioLegend | 109818 |
| M | **CD45.2** | **BV421** | 104 | BD | [562895](https://www.bdbiosciences.com/us/p/562895) |
| H | **CD45RA** | **APC** | HI100 | Biolegend | 304112 |
| H | **CD45RA** | **APC** | HI100 | BD | [561884](http://www.bdbiosciences.com/us/p/561884) |
| H | **CD45RA** | **BV650** | HI101 | Biolegend | 304135 |
| H | **CD45RO** | **APC** | UCHL1 | BioLegend | [304210](https://www.biolegend.com/en-us/search-results/apc-anti-human-cd45ro-antibody-856) |
| H | **CD45RO** | **BUV496** | UCHL1 | BD | [749888](https://www.bdbiosciences.com/us/reagents/research/antibodies-buffers/buv496-mouse-anti-human-cd45ro-uchl1/p/749888) |
| H | **CD45RO** | **BV605** | UCHL1 | Biolegend | 304238 |
| H | **CD45RO** | **Pacific Blue** | UCHL1 | BioLegend | [304216](https://www.biolegend.com/en-us/search-results/pacific-blue-anti-human-cd45ro-antibody-3342) |
| H | **CD45RO** | **PE** | UCHL1 | Biolegend | 304205 |
| H | **CD45RO** | **PerCP/Cy5.5** | UCHL1 | BD | [560607](http://www.bdbiosciences.com/eu/applications/research/b-cell-research/surface-markers/human/percp-cy55-mouse-anti-human-cd45ro-uchl1/p/560607) |
| H | **CD62L** | **AF488** | DREG-56 | Biolegend | 304816 |
| M | **CD62L** | **APC** | MEL-14 | Biolegend | 104411 |
| H | **CD62L** | **APC** | DREG-56 | Biolegend | 204809 |
| H | **CD62L** | **FITC** | DREG-56 | BD | [561914](http://www.bdbiosciences.com/us/applications/research/t-cell-immunology/regulatory-t-cells/surface-markers/human/fitc-mouse-anti-human-cd62l-dreg-56/p/561914) |
| H | **CD62L** | **PerCP/Cy5.5** | DREG-56 | Biolegend | 304824 |
| H | **CD73** | **BV421** | AD2 | BioLegend | 344007 |
| M | **CD73** | **Horizon V450** | TY/23 | BD | [561544](http://www.bdbiosciences.com/us/applications/research/stem-cell-research/mesenchymal-stem-cell-markers-bone-marrow/mouse/positive-markers/v450-rat-anti-mouse-cd73-ty23/p/561544) |
| H | **CD73** | **PerCP/Cy5.5** | AD2 | Biolegend | 344013 |
| M | **CD8** | **BV510** | 53-6.7 | BioLegend | [100751](https://www.biolegend.com/en-gb/products/brilliant-violet-510-anti-mouse-cd8a-antibody-7992) |
| H | **CD8a** | **AF700** | HIT8a | Biolegend | 300920 |
| M | **CD8a** | **BUV395** | 53-6.7 | BD | 565968 |
| H | **CD8a** | **BUV395** | RPA-T8 | BD | [563796](https://www.bdbiosciences.com/us/reagents/research/antibodies-buffers/immunology-reagents/anti-human-antibodies/cell-surface-antigens/buv395-mouse-anti-human-cd8-rpa-t8/p/563796) |
| H | **CD8a** | **BV510** | SK1 | BD | [563919](https://www.bdbiosciences.com/eu/p/563919) |
| H | **CD95** | **PerCP/Cy5.5** | DX2 | Biolegend | 305630 |
| H | **CD95** | **BV421** | DX2 | BD | [566258](http://www.bdbiosciences.com/us/applications/research/t-cell-immunology/regulatory-t-cells/surface-markers/human/bv421-mouse-anti-human-cd95-dx2/p/566258) |
| M | **CD95 (Fas)** | **AF488** | 15A7 | Thermo | [53-0951](https://www.thermofisher.com/antibody/product/CD95-APO-1-Fas-Antibody-clone-15A7-Monoclonal/53-0951-82) |
| M | **CTLA-4** | **APC** | UC10-4F10-11 | BD | [564331](http://www.bdbiosciences.com/us/reagents/research/antibodies-buffers/immunology-reagents/anti-mouse-antibodies/cell-surface-antigens/apc-hamster-anti-mouse-cd152-uc10-4f10-11/p/564331) |
| M | **CXCR4** | **PE** | L276F12 | BioLegend | [146506](https://www.biolegend.com/en-us/products/pe-anti-mouse-cd184-cxcr4-antibody-9057) |
| M | **EOMES** | **AF488** | DAN11MAG | eBiosciences | 53-4875-82 |
| M/H | **Eomes** | **PE** | Dan11mag | Thermo | [1086933](https://www.thermofisher.com/antibody/product/EOMES-Antibody-clone-Dan11mag-Monoclonal/12-4875-80) |
| H | **EOMES** | **PerCP/eFluor710** | WD1928 | Thermo | 46-4877-42 |
| M | **F(ab')2** | **PE** |  | Invitrogen | [A10543](https://www.thermofisher.com/antibody/product/Goat-anti-Mouse-IgG-H-L-Cross-Adsorbed-Secondary-Antibody-Polyclonal/A10543) |
| M | **Fc Block** | **non-conjugated** | 93 | BioLegend | [101320](https://www.biolegend.com/trustain-fcx-anti-mouse-cd16-32-antibody-5683.html) |
| H | **Fc Block** | **non-conjugated** |  | BioLegend | [422302](https://www.biolegend.com/en-us/products/human-trustain-fcx-fc-receptor-blocking-solution-6462) |
| M/H | **FoxP3** | AF488 | 150D | BioLegend | 32001 |
| M | **FoxP3** | **APC** | FJK-16s | Thermo | [17-5773-80](https://www.thermofisher.com/antibody/product/FOXP3-Antibody-clone-FJK-16s-Monoclonal/17-5773-80) |
| M | **Granzyme C** | **FITC** | SFC1D8 | BioLegend | [150805](https://www.biolegend.com/en-us/products/fitc-anti-mouse-granzyme-c-antibody-15319) |
| M/H | **GzmB** | **Pacific Blue** | QA16A02 | Biolegend | 372217 |
| M/H | **GzmB** | **PE** | QA16A02 | Biolegend | [372208](https://www.biolegend.com/en-us/products/pe-anti-human-mouse-granzyme-b-recombinant-antibody-14431) |
| M/H | **H3K27Ac** | **PE** | D5E4 | CST | 15562S |
| M/H | **H3K27Me3** | **AF647** | C36B11 | CST | 12158S |
| M/H | **H3K4Me3** | **PE** | C42D8 | CST | 62255S |
| M/H | **H3K9Ac** | **AF488** | C5B11 | CST | 9683S |
| M/H | **H3K9me2** | **AF647** | D85B4 | CST | 66070S |
| M/H | **ICOS** | **PerCP/Cy5.5** | C398.4A | BioLegend | 313518 |
| M | **IFN-γ** | **APC** | XMG1.2 | BioLegend | [505810](https://www.biolegend.com/apc-anti-mouse-ifn-gamma-antibody-993.html) |
| M | **IFN-γ** | **PE** | XMG1.2 | Biolegend | 505808 |
| M | **IL-17a** | **AF488** | TC11-18H10 | BioLegend | 506910 |
| M | **LAG3** | **PE** | C9B7W | BD | [552380](http://www.bdbiosciences.com/us/p/552380) |
| H | **LAG3** | **AF647** | 11C3C65 | Biolegend | 369304 |
| M | **Ly-6C** | **PE/Cy7** | AL-21 | BD | [560593](https://www.bdbiosciences.com/us/reagents/research/antibodies-buffers/immunology-reagents/anti-mouse-antibodies/cell-surface-antigens/pe-cy7-rat-anti-mouse-ly-6c-al-21/p/560593) |
| M | **LY6G** | **BV605** | 1A8 | Biolegend | 127639 |
| M | **MHC Class II** | **BV650** | M5/114.15.2 | BD | 562010 |
| M | **NK1.1** | **PE** | PK136 | Biolegend | 108726 |
| H | **PD1** | **PE/Dazzle594** | EH12.2H7 | Biolegend | 329939 |
| M | **PD-1** | **PE/Cy7** | 29F.1A12 | Biolegend | 135215 |
| H | **PD1** | **AF488** | EH12.2H7 | Biolegend | 329936 |
| M | **Perforin** | **APC** | eBioOMAK-D | Biolegend | 17-9392-80 |
| H | **Perforin** | **BV510** | dG9 | Biolegend | 308119 |
| H | **Perforin** | **Pacific Blue** | dG9 | Biolegend | [308117](https://www.biolegend.com/en-us/search-results/pacific-blue-anti-human-perforin-antibody-7265) |
| H | **Perforin** | **PerCP/Cy5.5** | dG9 | Biolegend | 308114 |
| M/H | **p-S6** | **AF488** | D57.2.2E | CST | [4803S](https://www.cellsignal.com/products/antibody-conjugates/phospho-s6-ribosomal-protein-ser235-236-d57-2-2e-xp-rabbit-mab-alexa-fluor-488-conjugate/4803) |
| M | **RORɣt** | **PE** | Q31-378 | BD | [562607](http://www.bdbiosciences.com/us/applications/research/t-cell-immunology/th17-cells/intracellular-markers/cell-signalling-and-transcription-factors/mouse/pe-mouse-anti-mouse-rort-q31-378/p/562607) |
| M/H | **STAT5** | **PE** | pY694 | BD | 612567 |
| M/H | **T-bet** | **PE/Dazzle594** | 4B10 | Biolegend | [644828](https://www.labome.com/product/BioLegend/644828.html) |
| M/H | **T-bet** | **PerCP/Cy5.5** | 4B10 | Biolegend | 644805 |
| H | **TCF1** | **BV421** | S33-966 | BD | S33-966 |
| M/H | **TCF1** | **PE** | S33-966 | BD | [564217](https://www.bdbiosciences.com/eu/reagents/research/antibodies-buffers/immunology-reagents/anti-mouse-antibodies/intracellular-antigens/pe-mouse-anti-tcf-7tcf-1-s33-966/p/564217) |
| M | **Thy-1.1** | **PE/Cy7** | HIS51 | eBiosciences | [25-0900-82](https://www.thermofisher.com/antibody/product/CD90-1-Thy-1-1-Antibody-clone-HIS51-Monoclonal/25-0900-82) |
| H | **TIGIT** | **PE/Cy7** | A15153G | Biolegend | 372713 |
| H | **TIM3** | **BV605** | F38-2E2 | Biolegend | 345017 |
| M | **TIM3** | **BV605** | RMT3-23 | Biolegend | 119721 |
| M | **TNF-α** | **PE/Cy7** | MP6-XT22 | BD | 561041 |
| M/H | **TOX** | **eFluor660** | TXRX10 | eBiosciences | 50-6502-82 |
| M | **Vα2 TCR** | **FITC** | B20.1 | BD | [553288](http://www.bdbiosciences.com/us/reagents/research/antibodies-buffers/immunology-reagents/anti-mouse-antibodies/cell-surface-antigens/fitc-rat-anti-mouse-v2-tcr-b201/p/553288) |
| M | **Vβ5.1/5.2 TCR** | **APC** | MR9-4 | Thermo | 17-5796 |
