# Supplementary material for: Oxygen levels at the time of activation determine T cell persistence and immunotherapeutic efficacy
Source: eLife. 2023 May 11;12:e84280. doi: 10.7554/eLife.84280 (PMC10229120; doi:10.7554/eLife.84280)
Supplement: Supplementary file 2. [file elife-84280-supp2.docx]

##### **Supplementary file 2**. List of antibodies used for western blot analysis.

| **Target** | **Catalog Number** | **Supplier** |
| --- | --- | --- |
| GLUT1 | ab115730 | abcam |
| Vinculin | ab219649 | abcam |
| HDAC | ab156064 | abcam |
| HIF-1α | 610958 | BD Biosciences |
| PPIB | 43603 | Cell Signaling Techonology |
| PPIB | 43603 | Cell Signaling Techonology |
| PGC1α | 4259 | Cell Signaling Techonology |
| NFIL3 | 14312 | Cell Signaling Techonology |
| NFAT1 | 4389 | Cell Signaling Techonology |
| NF-κB | 8242 | Cell Signaling Techonology |
| cMYC | 5605 | Cell Signaling Techonology |
| LDHA | 3582 | Cell Signaling Techonology |
| Lamin b1 | 13435 | Cell Signaling Techonology |
| HIF-1α | NB-100-449 | Novus |
| HIF-2α | AF2997 | R&D Systems |
