# Supplementary material for: Oxygen levels at the time of activation determine T cell persistence and immunotherapeutic efficacy
Source: eLife. 2023 May 11;12:e84280. doi: 10.7554/eLife.84280 (PMC10229120; doi:10.7554/eLife.84280)
Supplement: Supplementary file 3. [file elife-84280-supp3.docx]

**Supplementary file 3**. List of protein sequences and DNA sequences corresponding to miRNA-embedded shRNAs used in viral vectors.

|  | **Aminoacid sequence** |
| --- | --- |
| **RQR8** | MGTSLLCWMALCLLGADHADACPYSNPSLCSGGGGSELPTQGTFSNVSTNVSPAKPTTTACPYSNPSLCSGGGGSPAPRPPTPAPTIASQPLSLRPEACRPAAGGAVHTRGLDFACDIYIWAPLAGTCGVLLLSLVITLYCNHRNRRRVCKCPRPVV |
| **T2A self-cleaving** | GSGEGRGSLLTCGDVEENPGP |
| **CD19 BBz CAR** | MALPVTALLLPLALLLHAARPDIQMTQTTSSLSASLGDRVTISCRASQDISKYLNWYQQKPDGTVKLLIYHTSRLHSGVPSRFSGSGSGTDYSLTISNLEQEDIATYFCQQGNTLPYTFGGGTKLEITGGGGSGGGGSGGGGSEVKLQESGPGLVAPSQSLSVTCTVSGVSLPDYGVSWIRQPPRKGLEWLGVIWGSETTYYNSALKSRLTIIKDNSKSQVFLKMNSLQTDDTAIYYCAKHYYYGGSYAMDYWGQGTSVTVSSTTTPAPRPPTPAPTIASQPLSLRPEACRPAAGGAVHTRGLDFACDIYIWAPLAGTCGVLLLSLVITLYCKRGRKKLLYIFKQPFMRPVQTTQEEDGCSCRFPEEEEGGCELRVKFSRSADAPAYKQGQNQLYNELNLGRREEYDVLDKRRGRDPEMGGKPRRKNPQEGLYNELQKDKMAEAYSEIGMKGERRRGKGHDGLYQGLSTATKDTYDALHMQALPPR |
| **HER2 (4D5) BBz CAR** | MALPVTALLLPLALLLHAARPMDFQVQIFSFLLISASVIMSRGDIQMTQSPSSLSASVGDRVTITCRASQDVNTAVAWYQQKPGKAPKLLIYSASFLYSGVPSRFSGSRSGTDFTLTISSLQPEDFATYYCQQHYTTPPTFGQGTKVEIKRTGSTSGSGKPGSGEGSEVQLVESGGGLVQPGGSLRLSCAASGFNIKDTYIHWVRQAPGKGLEWVARIYPTNGYTRYADSVKGRFTISADTSKNTAYLQMNSLRAEDTAVYYCSRWGGDGFYAMDVWGQGTLVTVSSTTTPAPRPPTPAPTIASQPLSLRPEACRPAAGGAVHTRGLDFACDIYIWAPLAGTCGVLLLSLVITLYCKRGRKKLLYIFKQPFMRPVQTTQEEDGCSCRFPEEEEGGCELRVKFSRSADAPAYKQGQNQLYNELNLGRREEYDVLDKRRGRDPEMGGKPRRKNPQEGLYNELQKDKMAEAYSEIGMKGERRRGKGHDGLYQGLSTATKDTYDALHMQALPPR |
|  | **DNA sequence** |
| **miRNA-embedded shNTC** | TGACGTACGGCGCGCGCCTCGACTAGGGATAACAGGGTAATTGTTTGAATGAGGCTTCAGTACTTTACAGAATCGTTGCCTGCACATCTTGGAAACACTTGCTGGGATTACTTCGACTTCTTAACCCAACAGAAGGCTCGAGAAGGTATATTGCTGTTGACAGTGAGCGCAGGAATTATAATGCTTATCTATAGTGAAGCCACAGATGTATAGATAAGCATTATAATTCCTATGCCTACTGCCTCGGACTTCAAGGGGCTAGAATTCGAGCAATTATCTTGTTTACTAAAACTGAATACCTTGCTATCTCTTTGATACATTTTTACAAAGCTGAATTAAAATGGTATAAATTAAATCACTTTTTTCAATTGACGCGTAATTCTACCTGA |
| **miRNA-embedded shVHL** | TGACGTACGGCGCGCGCCTCGACTAGGGATAACAGGGTAATTGTTTGAATGAGGCTTCAGTACTTTACAGAATCGTTGCCTGCACATCTTGGAAACACTTGCTGGGATTACTTCGACTTCTTAACCCAACAGAAGGCTCGAGAAGGTATATTGCTGTTGACAGTGAGCGCGATCTGGAAGACCACCCAAATTAGTGAAGCCACAGATGTAATTTGGGTGGTCTTCCAGATCATGCCTACTGCCTCGGACTTCAAGGGGCTAGAATTCGAGCAATTATCTTGTTTACTAAAACTGAATACCTTGCTATCTCTTTGATACATTTTTACAAAGCTGAATTAAAATGGTATAAATTAAATCACTTTTTTCAATTGACGCGTAATTCTACCTGA |
